# Novel CDK-independent function of CDC25 phosphatases in mRNA translation

**DOI:** 10.1038/s44319-026-00852-y

**Published:** 2026-07-14

**Authors:** Sauyeun Shin, Marie Cargnello, Lisa Pinchedé, Claire Medale Giamarchi, Cléa Villette, Alexandre Gay, Virginie Salnot, Emilie-Fleur Gautier, Erik Dassi, Stefania Millevoi, Anne Cammas, Stéphane Manenti

**Affiliations:** 1 University of Toulouse, INSERM, CNRS, CRCT, Toulouse, France; 2 Equipe labellisée 2016-2020, La Ligue contre le Cancer, Toulouse, France; 3 Proteom’IC facility, Université Paris Cité, CNRS, INSERM, Institut Cochin, Paris, France; 4 Laboratory of RNA Regulatory Networks, Department of Cellular, Computational and Integrative Biology (CIBIO), University of Trento, Trento, Italy

**Keywords:** Translation & Protein Quality

## Abstract

Molecular and functional networks driving coordination between cell cycle and mRNA translation remain to be explored. Here, we use mass spectrometry-based proteomics to comprehensively investigate the interactome and phosphoproteome of the cell cycle regulator CDC25A. We identify actors of mRNA regulation, such as RNA-binding proteins and translation factors, as interacting partners of CDC25A. CDC25A overexpression increases global translation, whereas catalytic inactivation or pharmacological inhibition decreases protein synthesis. A Cyclin-Dependent Kinase (CDK) interaction-deficient mutant of CDC25A also enhances translation, indicating a CDK-independent role. Our results further reveal an interplay between CDC25A and CDC25B whereby downregulation of CDC25A leads to compensatory overexpression of CDC25B. The roles of CDC25A and CDC25B in mRNA translation are independent of their roles in the cell cycle, with CDC25A possibly regulating translation elongation and CDC25B rather involved in initiation. In acute myeloid leukemia cells, CDC25A depletion also inhibits translation, suggesting its potential relevance as a therapeutic target. We propose that CDC25 phosphatases might be signaling platforms coordinating cell cycle progression with protein synthesis.

## Synopsis

CDC25A and CDC25B phosphatases regulate mRNA translation elongation and initiation, respectively. This occurs independently of their function of Cyclin-Dependent Kinases regulation during the different phases of the cell cycle, revealing a moonlighting role for CDC25 proteins in RNA expression regulation.

- CDC25A protein interactome underscores its role in RNA regulation
- CDC25A and CDC25B regulate mRNA translation elongation and initiation by modulating elongation factors and signaling pathways, respectively.
- Translational regulation by CDC25A and CDC25B is intertwined, CDK-independent, and occurs across the cell cycle.

## Abstract (web summary)

CDC25A and CDC25B phosphatases regulate mRNA translation elongation and initiation, respectively. This occurs independently of their function of Cyclin-Dependent Kinases regulation during the different phases of the cell cycle, revealing a moonlighting role for CDC25 proteins in RNA expression regulation.

## Graphical abstract

**Figure:**
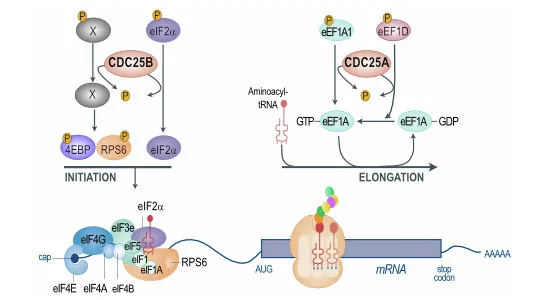

**Figure:**
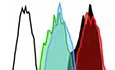

## Introduction

Eukaryotic cells tightly regulate cell cycle progression in order to avoid cell death or deleterious defects transmitted to daughter cells. This implicates a finely tuned coordination between key biological functions, like gene expression, DNA replication, energetic and oxidative metabolism, mRNA translation and protein synthesis, or cell growth. mRNA translation is a high-energy-consuming process that needs to be optimized and tightly adapted to the cell context. Although molecular and functional relationships between mRNA translation and cell cycle progression have been described (Aramayo and Polymenis, 2017), they still remain to be completed. Several studies have described changes in mRNA translation during the cell cycle, in particular a decrease in translation during mitosis (Kim et al, 2014), but recent works performed on asynchronous cells rather argue for no major variation in global translation and protein synthesis during the different phases of the cell cycle (Stonyte et al, 2018). At the molecular level, cell cycle actors have been involved in mRNA translation, like Cyclin B1/CDK1, for instance, for the regulation of cap-dependent translation through phosphorylation of 4EBP1 (Sun et al, 2019). Conversely, translational regulations of cell cycle actors like cyclins have been described (Tarn and Lai, 2011; Stumpf et al, 2013), and CDC25A translation is positively regulated by Boll protein during meiosis (Lin et al, 2009).

Sequential activation of cyclin-dependent kinases is crucial for cell cycle progression (Malumbres and Barbacid, 2005). Removal of Wee1-mediated inhibitory phosphorylations on CDKs is mediated by dual-specificity phosphatases of the CDC25 family, composed of the three isoforms CDC25A, CDC25B and CDC25C in mammals (Boutros et al, 2007; Kiyokawa and Ray, 2008). These isoforms cooperate to induce mitosis, but CDC25A also regulates progression in G1, G1/S transition, and S phase completion, by sequentially activating Cyclin D/CDK4 and cyclinE/A-CDK2 (Boutros et al, 2007). As a major actor of G1, CDC25A is the only CDC25 to be regulated by extracellular mitogenic signals provided by growth factors, cytokines, and extracellular matrix such as fibronectin (Bhowmick et al, 2003; Fernandez-Vidal et al, 2006; Kittipatarin et al, 2006). Murine knockout models revealed some functional redundancy between CDC25 phosphatases. Indeed, double knockout CDC25B^–/–^- CDC25C^–/–^ mice develop normally, and cells from these mice display normal cell cycle profiles (Ferguson et al, 2005). On the contrary, CDC25A knockout is lethal at a very early stage during embryogenesis (Ray et al, 2007b), suggesting non-redundant functions during development. CDC25A therefore appears to fulfill the most important functions compared to the other CDC25 isoforms. Nonetheless, CDC25A overexpression leads to genomic instability and cooperates with the Neu-Ras oncogenic pathway in mammary tumorigenesis (Ray et al, 2007a), while hemizygous disruption of *Cdc25A* inhibits cellular transformation and mammary tumorigenesis in mice (Ray et al, 2007b). Overexpression of CDC25A mRNA and protein has been reported in various human cancers, often associated with high-grade tumors and poor prognosis (Boutros et al, 2007). These deregulations of CDC25A expression in cancers originate from increased transcription, deregulation of miRNAs, or protein stabilization (Shen and Huang, 2012).

Although the main molecular function of CDC25A, which has been extensively studied during the past decades, remains the dephosphorylation and activation of CDKs, sparse evidence in the literature suggests additional roles in apoptosis, invasion, glycolytic metabolism, or quiescence. CDC25A was reported to ensure both anti- and pro-apoptotic functions. Indeed, CDC25A can directly interact with the ASK1 protein kinase to inhibit its activity and its apoptotic function, through a mechanism that seems independent of its catalytic activity (Zou et al, 2001), and inversely, in response to pro-apoptotic stress, a caspase-dependent cleavage of CDC25A generates a C-terminal active fragment with pro-apoptotic function (Mazars et al, 2009). CDC25A was also reported to be implicated in promoting metastasis through the CDK2/FOXO1/MMP1 cascade (Feng et al, 2011). In a more recent work, Liu et al demonstrate that EGFR activation results in dephosphorylation of PKM2 at S37 by CDC25A, promoting PKM2-dependent β-catenin transactivation and c-Myc-dependent upregulated expression of the glycolytic genes GLUT1, PKM2, and LDHA. Consequently, CDC25A-mediated PKM2 dephosphorylation promotes the Warburg effect, cell proliferation, and brain tumorigenesis (Liang et al, 2016). This work represents one of the very few examples of CDC25A substrates other than CDKs. Taken together, these studies suggest that CDC25A is able to play secondary functions different from cell cycle regulation.

Based on these different studies, and through analysis of recent data obtained by us and others on CDC25A regulations and functions in different pathophysiological models, we were concerned by the fact that simple regulation of CDK activity could not easily account for the diversity of effects observed upon CDC25A inhibition or overexpression. Consequently, the aim of this work was to investigate and identify new functions of CDC25A. Proteomic studies performed in HEK293 and in acute myeloid leukemia (AML) cells, in which we had previously demonstrated the importance of CDC25A (Fernandez-Vidal et al, 2006; Bertoli et al, 2015; Sueur et al, 2020), revealed the impact of CDC25A inhibition on mRNA-regulating proteins, including RNA-binding proteins and translation-regulating factors. Inhibition or overexpression experiments suggest direct involvement of CDC25A and CDC25B in mRNA translation, independently of their well-characterized function as CDK activators. These data identify CDC25A and CDC25B as important candidates to coordinate cell cycle progression with mRNA translation.

## Results

### CDC25A interacts with the translation machinery

**Figure EV1:**
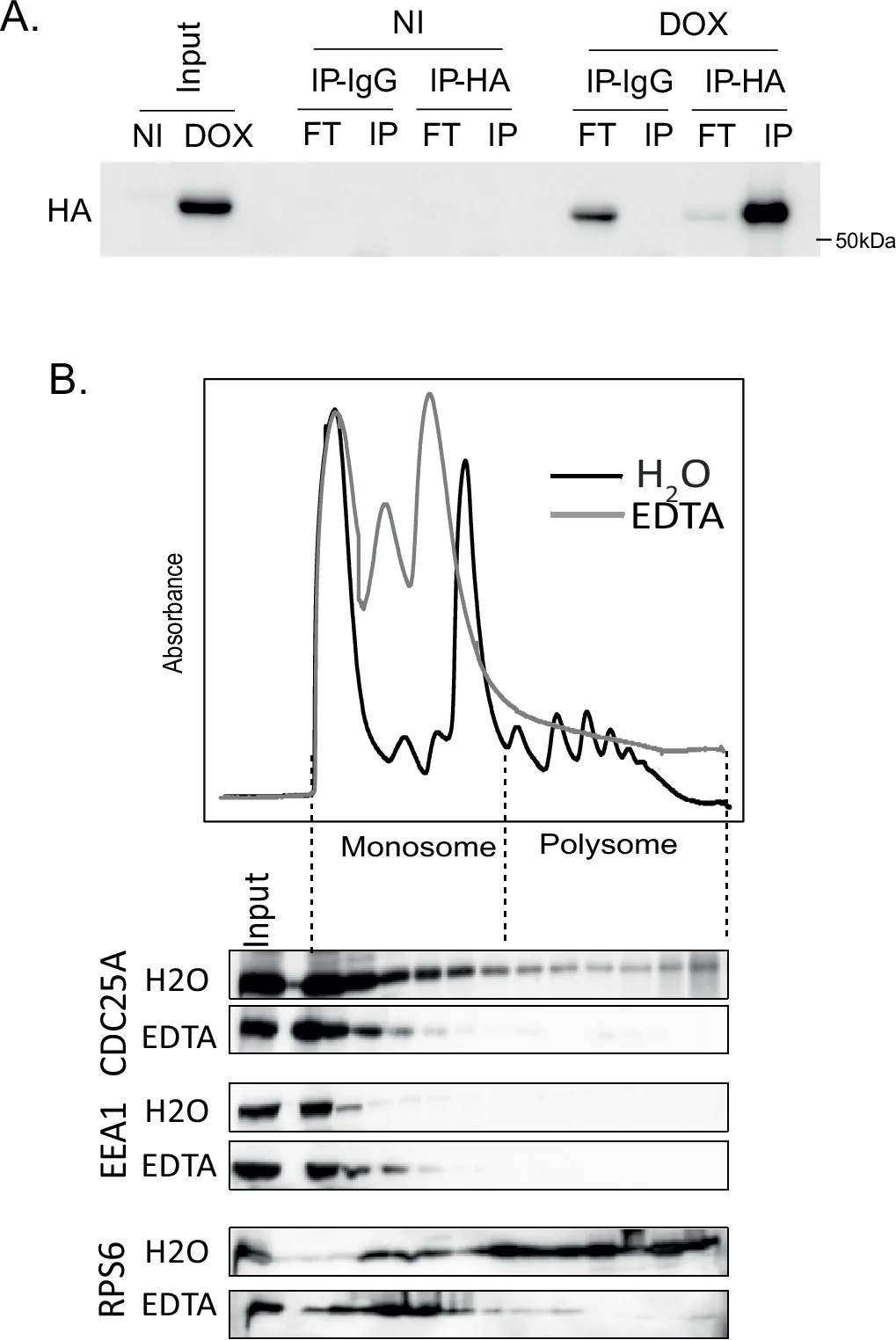
CDC25A immunoprecipitation for its interactome analysis. (**A**) HEK293 cells were induced for HA-CDC25A expression for 24 h with doxycycline 1μg/ml (DOX) or left non-induced (NI). Immunoprecipitation of HA-CDC25A from cell extracts was performed with an anti-HA antibody. Aliquots of the inputs, flow through (FT), and immunoprecipitates (IP) were analyzed by western blot with the anti-HA antibody to check the efficiency of the immunoprecipitation. (**B**) Polysome sedimentation profile of HEK293 cells induced for HA-CDC25A expression for 24 h (DOX; upper panel), and western blot analysis of HA-CDC25A in the monosomal and polysomal fractions (lower panel), in the absence (H2O) or the presence of EDTA. Western blots of EEA1 and RPS6 were used as negative and positive controls, respectively. Shown is a representative result from *n* = 2 independent experiments. Source data are available online for this figure.

**Figure 1:**
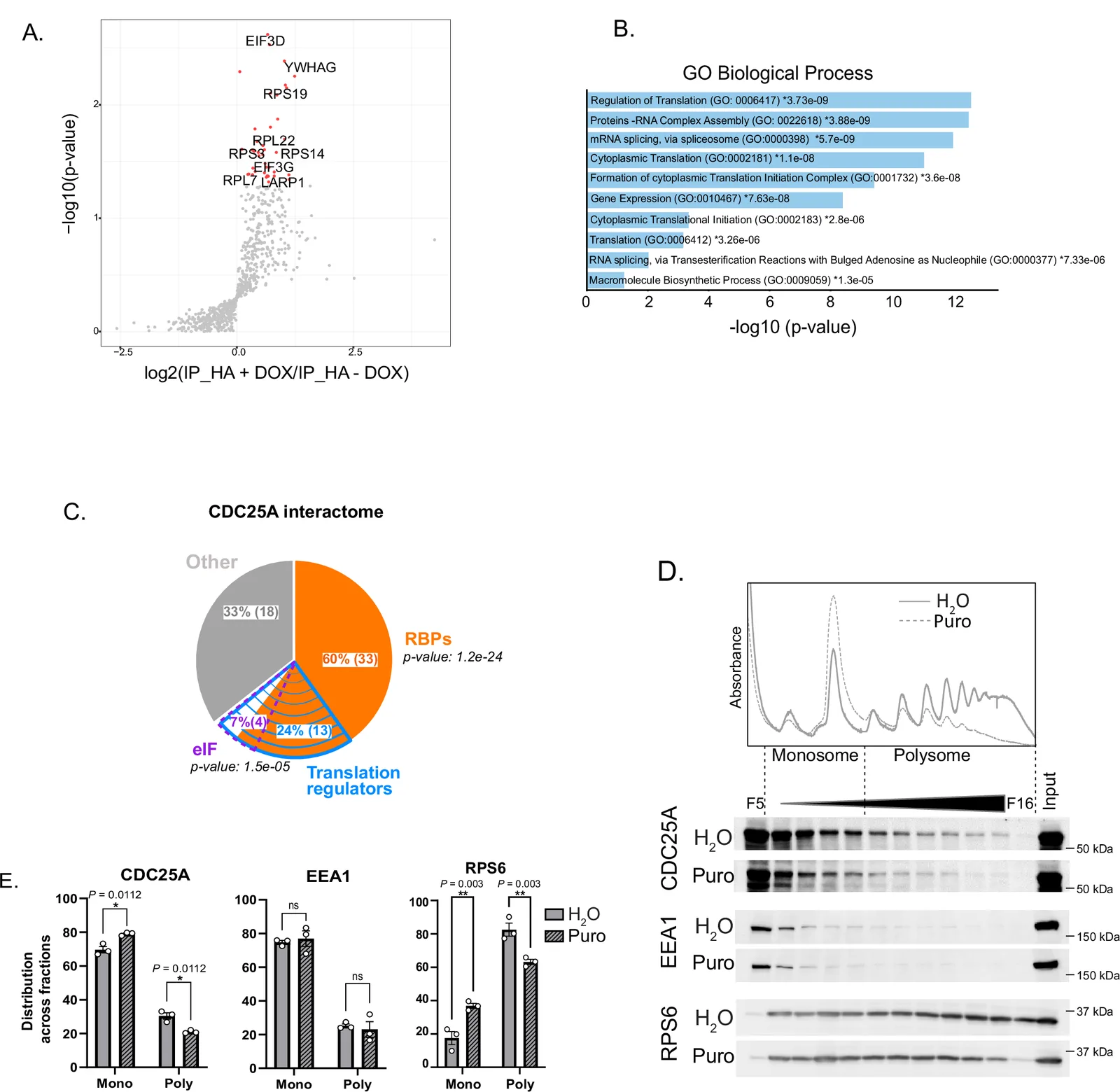
Translation factors interact with CDC25A in HEK293 cells. (**A**) Volcano plot showing differentially immunoprecipitated (IP) proteins using HA antibody from extracts of doxycycline-induced HEK293 cells expressing HA-tagged CDC25A ( + DOX) versus non-induced HEK293 cells (-DOX) and identified by mass spectrometry (IP-MS, *n* = 3 independent experiments). Proteins significantly immunoprecipitated (*P* value < 0.05) with HA-CDC25A are highlighted with red dots. The indicated proteins belong to the Gene Ontology term cytoplasmic translation (GO: 0002181) and translation (GO:0006412). The *x* axis displays the log2 fold change of the LFQ intensities, and the *P* values are represented on −log10 scale on the *y* axis. A paired one-tailed Welch *t* test was performed on proteins with at least 60% paired valid values using PROXIMA R shiny application. (**B**) −log10(*P* value) of the enrichment analysis of CDC25A interactome using Enrichr (Kuleshov et al, 2016). *P* value computed from the Fisher's exact test. (**C**) Distribution of CDC25A partners identified in (**A**). The number, percentage, and *P* values (Fisher test) for the RNA-binding proteins (RBPs) and translation initiation factors are indicated. (**D**) Polysome sedimentation profile of HEK293 cells induced for HA-CDC25A expression for 24 h (DOX; upper panel), and western blot analysis of HA-CDC25A in the monosomal and polysomal fractions (lower panel), in the absence (H_2_O) or the presence of puromycin (Puro). Western blots of EEA1 and RPS6 were used as negative and positive controls, respectively. Shown is a representative result from *n* = 3 independent experiments. (**E**) Quantification of CDC25A, EEA1, and RPS6 western blots in monosomal (Mono: fraction 6–9) and polysomal (Poly: fraction 10–16) fractions in the presence (Puro) or the absence (H_2_O) of puromycin. Data are presented as mean values ± SEM of *n* = 3 independent experiments, *P < 0.05, **<0.01; ns: non-significant (repeated measures two-way ANOVA). Source data are available online for this figure.

To explore novel functions of CDC25A phosphatase, we analyzed the CDC25A protein interactome by performing immunoprecipitation of a doxycycline-inducible HA-tagged CDC25A expressed in HEK293 cells (Mazzolini et al, 2016) (Fig. EV1A). Proteins co-immunoprecipitated with HA-tagged CDC25A were subjected to tryptic digestion followed by nano-LC-MS/MS analysis, thus allowing DIA-PASEF quantitative label-free proteomic analysis of protein-protein interaction data. This quantitative analysis with three replicates enabled the identification of 55 proteins that specifically and significantly (*P* value ≤ 0.05) interact with HA-CDC25A (Fig. 1A; Dataset EV1). Pathway enrichment analysis showed that these CDC25A protein partners were significantly assigned not only to CDC25A canonical functions, but also to specific post-transcriptional pathways related to mRNA translation and RNA-binding (Fig. 1A,B). Among these proteins, we found 33 RBPs, 13 translation regulators, among which four subunits of the eIF3 complex that are involved in both translation initiation (Jackson et al, 2010) and elongation (Lin et al, 2020) (Fig. 1C; Dataset EV1). The observation that CDC25A interacts with RBPs and translational initiation factors prompted us to investigate its physical interaction with the translational machinery. To this end, we performed a sucrose gradient-based polysome purification, combined with immunoblotting to monitor the distribution of HA-tagged CDC25A in translationally inactive and active fractions in the presence or absence of puromycin, a drug inducing ribosome dissociation (Fig. 1D,E). We found that HA-tagged CDC25A co-sedimented with translating polyribosomes and that its association depended on polysome integrity (Fig. 1D,E), similarly to the positive control RPS6, and contrary to EEA1, an endosomal protein that we already used as a negative control in previous publications (Herviou et al, 2020). This conclusion was further strengthened by using EDTA, a Mg2+ chelator that globally dissociates ribosomal subunits and destabilizes a broad range of RNA-protein interactions, which produced stronger effects on CDC25A polysomal association (Fig. EV1B). Altogether, these results revealed an association of CDC25A with the translational machinery that might underline new functions of this phosphatase in mRNA translation.

### CDC25A regulates mRNA translation independently of its interaction with CDKs

**Figure 2:**
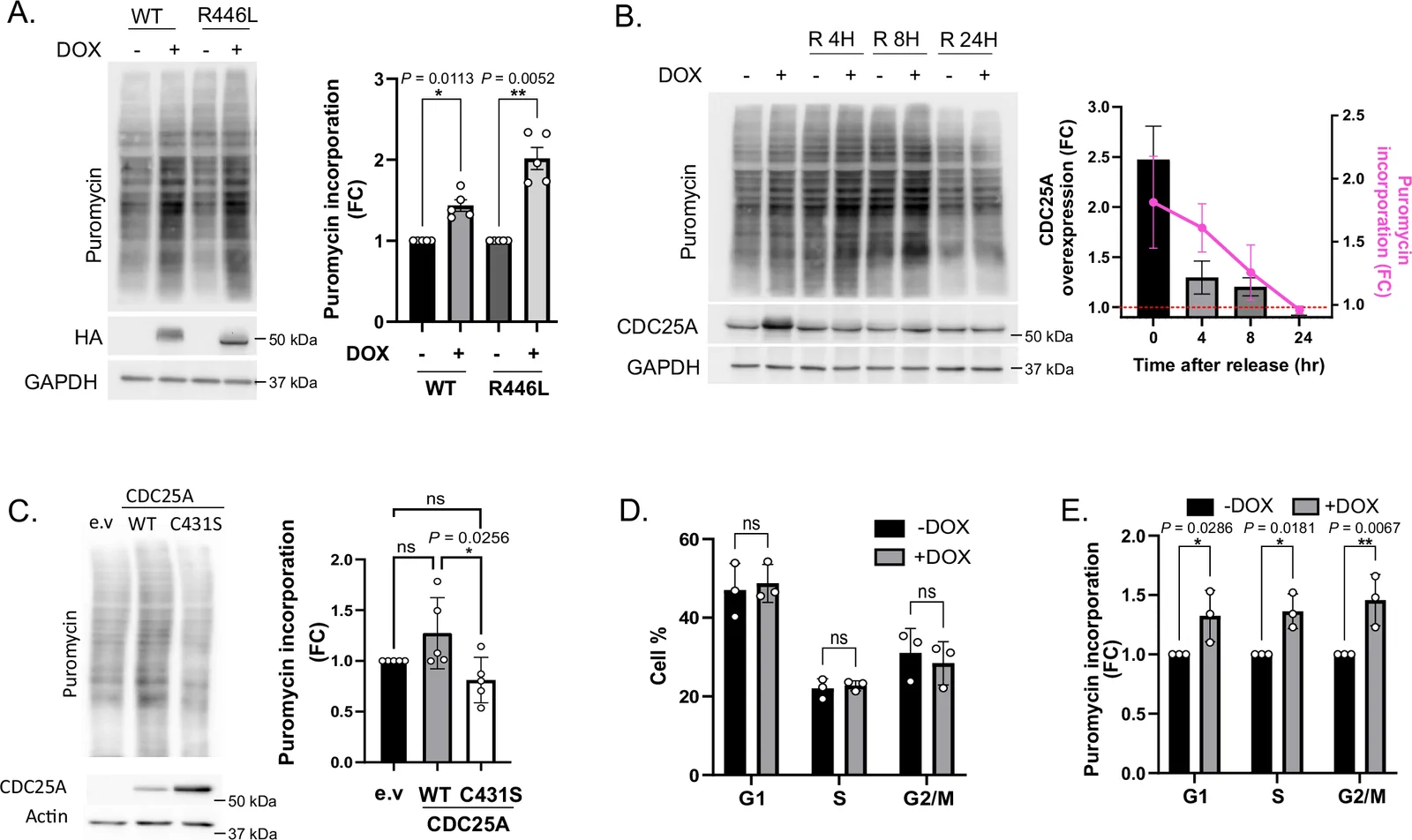
CDC25A overexpression promotes global translation independently of its interaction with CDK. (**A**) Western blot SUnSET analysis (left panel) and quantification (right panel) of de novo protein synthesis (global translation) in response to wt-CDC25A (WT) or R446L-CDC25A (R446L) mutant induction (DOX) in HEK293 cells. Data are presented as mean values ± SEM of *n* = 5 independent experiments, *P < 0.05; **P < 0.01; ns: non-significant (one-way ANOVA). (**B**) Western blot SUnSET analysis (left panel) and quantification (right panel) of the impact of doxycyclin removal (R) for 4, 8, and 24 h. Data are presented as mean values ± SEM of *n* = 3 independent experiments. (**C**) Western blot SUnSET analysis (left panel) and quantification (right panel) of de novo protein synthesis (global translation) in response to the transfection of empty vector (e.v), wt-CDC25A (WT), or Mut-CDC25A (C431S) in HEK293 cells. Data are presented as mean values ± SEM of *n* = 3 independent experiments, *P < 0.05; ns: non-significant (one-way ANOVA). (**D**) Flow cytometry cell cycle analysis (DAPI labeling) of HEK293 cells in response to CDC25A induction (DOX). Data are presented as mean values ± SEM of *n* = 3 independent experiments, ns: non-significant (two-way ANOVA). (**E**) Flow cytometry analysis of puromycin incorporation in response to CDC25A overexpression (DOX) during G1, S, and G2/M phases of the cell cycle. Data are presented as mean values ± SEM of *n* = 3 independent experiments, *P < 0.05, **P < 0.01; ns: non-significant (two-way ANOVA). Source data are available online for this figure.

**Figure EV2:**
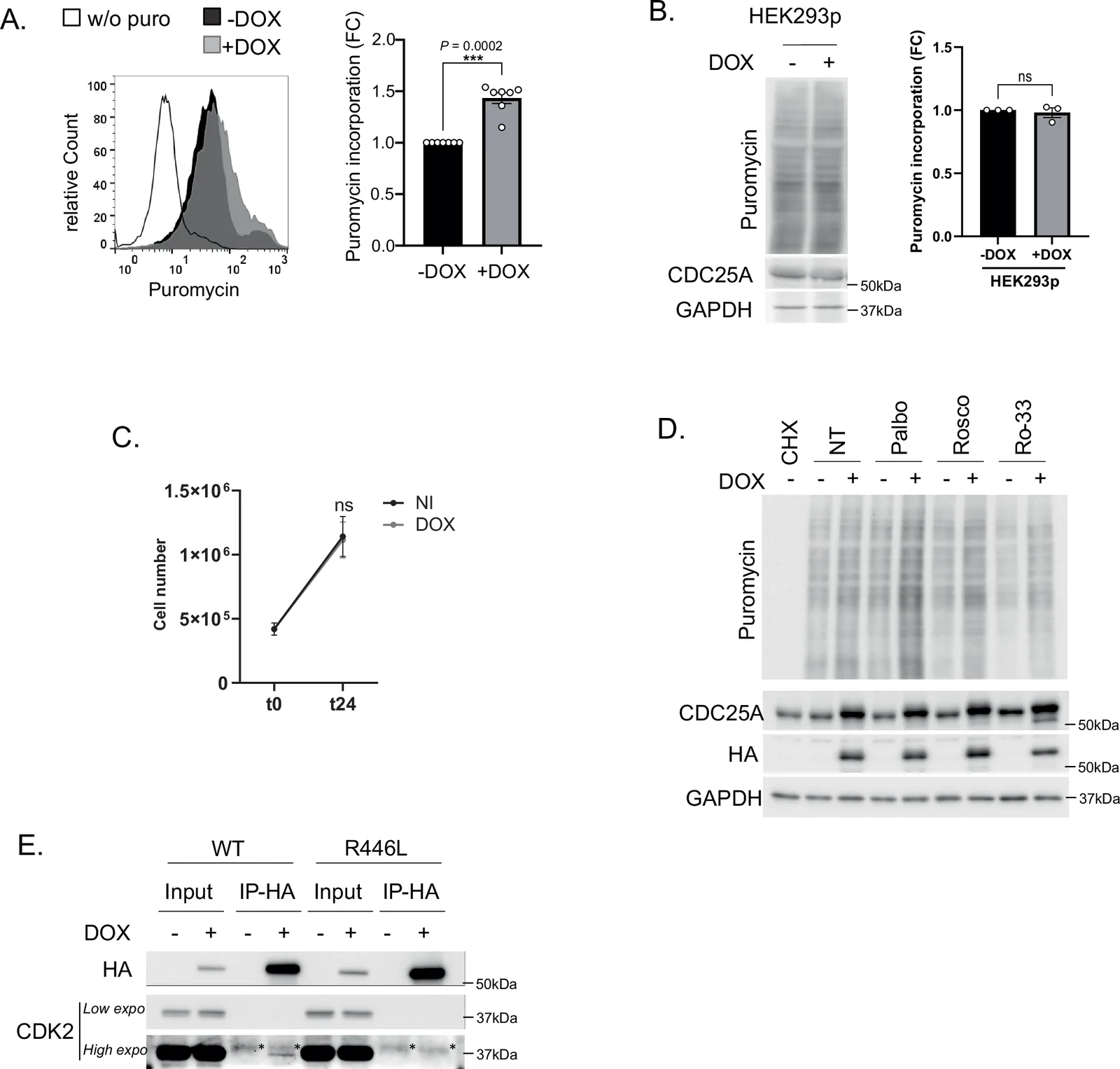
CDC25A overexpression promotes global translation independently of the cell cycle. (**A**) Flow cytometry SUnSET analysis (left panel) and quantification (right panel) of global translation in response to CDC25A induction (DOX) in HEK293 cells. Data are presented as mean values ± SEM of *n* = 7 independent experiments, ***P < 0.0001, ns: non-significant (two-sided paired *t* test). (**B**) Western blot SUnSET analysis of puromycin incorporation in response to doxycycline treatment in parental HEK293 (HEK293p) cell line. Data are presented as mean values ± SEM of *n* = 3 independent experiments, ns: non-significant (two-sided paired *t* test). (**C**) Impact of CDC25A induction (DOX) for 24 h on total HEK293 cell number. NI not induced. Data are presented as mean values ± SEM of *n* = 3 independent experiments, ns: non-significant (two-sided paired *t* test). (**D**) Western blot SUnSET analysis of puromycin incorporation in HEK293 cells induced for CDC25A expression (DOX) for 24 h, and further treated for four additional hours with the CDK inhibitors Palbocyclib (Palbo; 1 μM), Roscovitine (Rosco;1 μM), and RO-3306 (RO-33; 10 μM). (**E**) Immunoprecipitation using anti-HA antibody of WT or R245L HA-CDC25A from cell extracts of HEK293 cells induced for HA-CDC25AWT or HA-CDC25A-R245L mutant (for 24 h) with doxycycline (DOX) or left non-induced (NI). Inputs, flow through (FT), and immunoprecipitates (IP) were analyzed by western blot with the anti-HA and CDK2 antibodies. *Non-specific (ns). Source data are available online for this figure.

In order to investigate the role of CDC25A in mRNA translation, we quantified the global protein synthesis by pulsed puromycin labeling of newly synthesized proteins (i.e., SUnSET assay) after the conditional expression of HA-tagged CDC25A in the HEK293 cell line. Western blot (Fig. 2A) and FACS (Fig. EV2A) analysis of puromycin incorporation in elongating peptides showed that after 24 h of treatment with doxycycline, translation was increased in cells overexpressing CDC25A, while doxycycline had no impact on the parental HEK293 cells translation level (Fig. EV2B). Moreover, removing doxycycline from the culture medium after 24 h of treatment reversed the effect of CDC25A overexpression on global translation (Fig. 2B), revealing a correlation between the variation of CDC25A expression and translation efficiency. To assess whether the phosphatase activity of CDC25A was responsible for this effect, we used a catalytically inactive mutant (C431S) of CDC25A (for a recent description of CDC25A structure, see (Rowland et al, 2024)). We observed a trend towards the increase of translation upon transient expression of the wtCDC25A, while expression of the mutant did not modify the translation rates of HEK293T cells (Fig. 2C). These results demonstrate that its catalytic phosphatase activity is necessary for CDC25A functions in translation.

To distinguish between the cell cycle and mRNA translation functions of CDC25A, we performed cell cycle analysis by FACS. Interestingly, CDC25A overexpression did not induce any detectable modification of the cell cycle profile (Fig. 2D) or cell proliferation rate (Fig. EV2C). Importantly, CDC25A expression did not increase translation in a specific phase but similarly all along the cell cycle (Fig. 2E), suggesting a CDK-independent function of CDC25A. Moreover, treatment with pharmacological inhibitors of CDK4/CDK6 (palbociclib), CDK2 (roscovitine), or CDK1 (Ro-3306) after 24 h of CDC25A induction did not modify the positive effect of CDC25A overexpression on global translation (Fig. EV2D), suggesting that CDKs are not implicated in CDC25A-mediated regulation of mRNA translation. To further confirm this important observation, we built a CDC25A R446L mutant deficient for its interaction with CDKs, as previously described by Sohn and Rudolph (Sohn and Rudolph, 2006) for CDC25B. We verified by co-immunoprecipitation experiments that the CDC25A R446L mutant does not interact with CDK2 (Fig. EV2E). As expected, overexpression of this CDC25A CDK interaction-deficient mutant (Fig. 2A) had a similar impact on translation compared to the wild type protein, confirming that the increase of mRNA translation is independent of CDC25A CDK-activating functions.

Altogether, these results demonstrate the existence of a CDK-independent function of the phosphatase CDC25A in mRNA translation.

### Both CDC25A and CDC25B regulate translation

**Figure 3:**
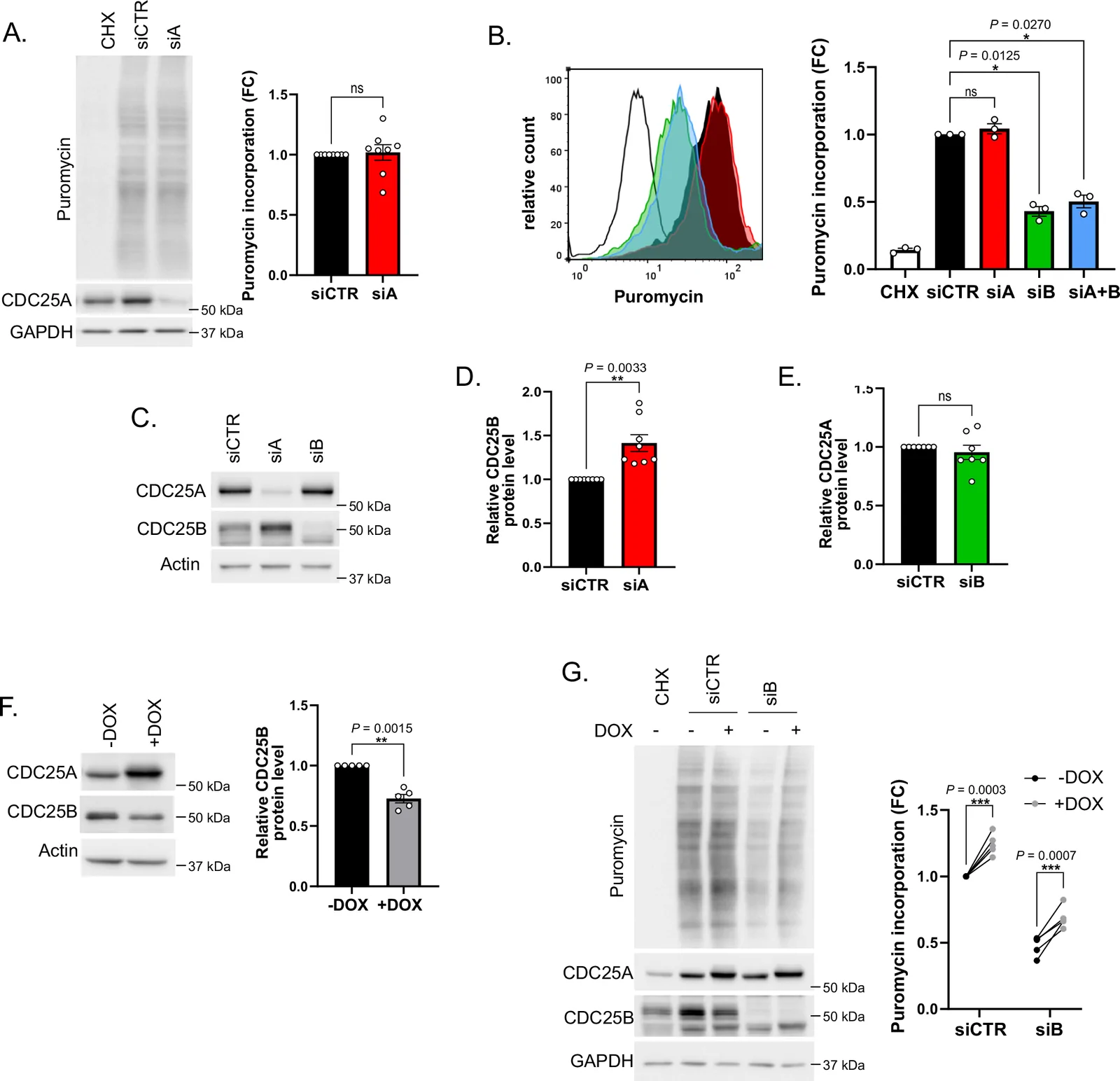
Compensation between CDC25A and CDC25B. (**A**, **B**) Western blot (**A**) and flow cytometry (**B**) analysis of puromycin incorporation in response to siRNA-mediated depletion of CDC25A (siA) or CDC25B (siB) in HEK293 cells. Cycloheximide treatment (CHX) was used as a positive control of translation inhibition. Data are presented as mean values ± SEM of *n* = 8 and 3 independent experiments for A and B, respectively, *P < 0.05, ns: non-significant (two-sided paired *t* test for (**A**); repeated measures two-way ANOVA for (**B**)). (**C**–**E**) Western blot analysis (**C**) and quantification (**D**, **E**) of reciprocal impact of CDC25A (siA) and CDC25B (siB) siRNA-mediated depletions. Data are presented as mean values ± SEM of *n* = 8 and 7 independent experiments for (**C**, **E**), respectively. For (**D**, **E**), **P < 0.01, ns: non-significant (two-sided paired *t* test). (**F**) Western blot analysis (left panel) and quantification (right panel) of CDC25B expression in response to doxycycline-induced HA-CDC25A expression (DOX). Data are presented as mean values ± SEM of *n* = 5 independent experiments, **P < 0.01 (two-sided paired *t* test). (**G**) Western blot analysis (left panel) and quantification (right panel) of the impact of siRNA-mediated CDC25B depletion (siB) on CDC25A-mediated (DOX) increase of translation (puromycin incorporation). Data are presented as mean values ± SEM of *n* = 5 independent experiments, ***P < 0.001; (two-way ANOVA). Source data are available online for this figure.

**Figure EV3:**
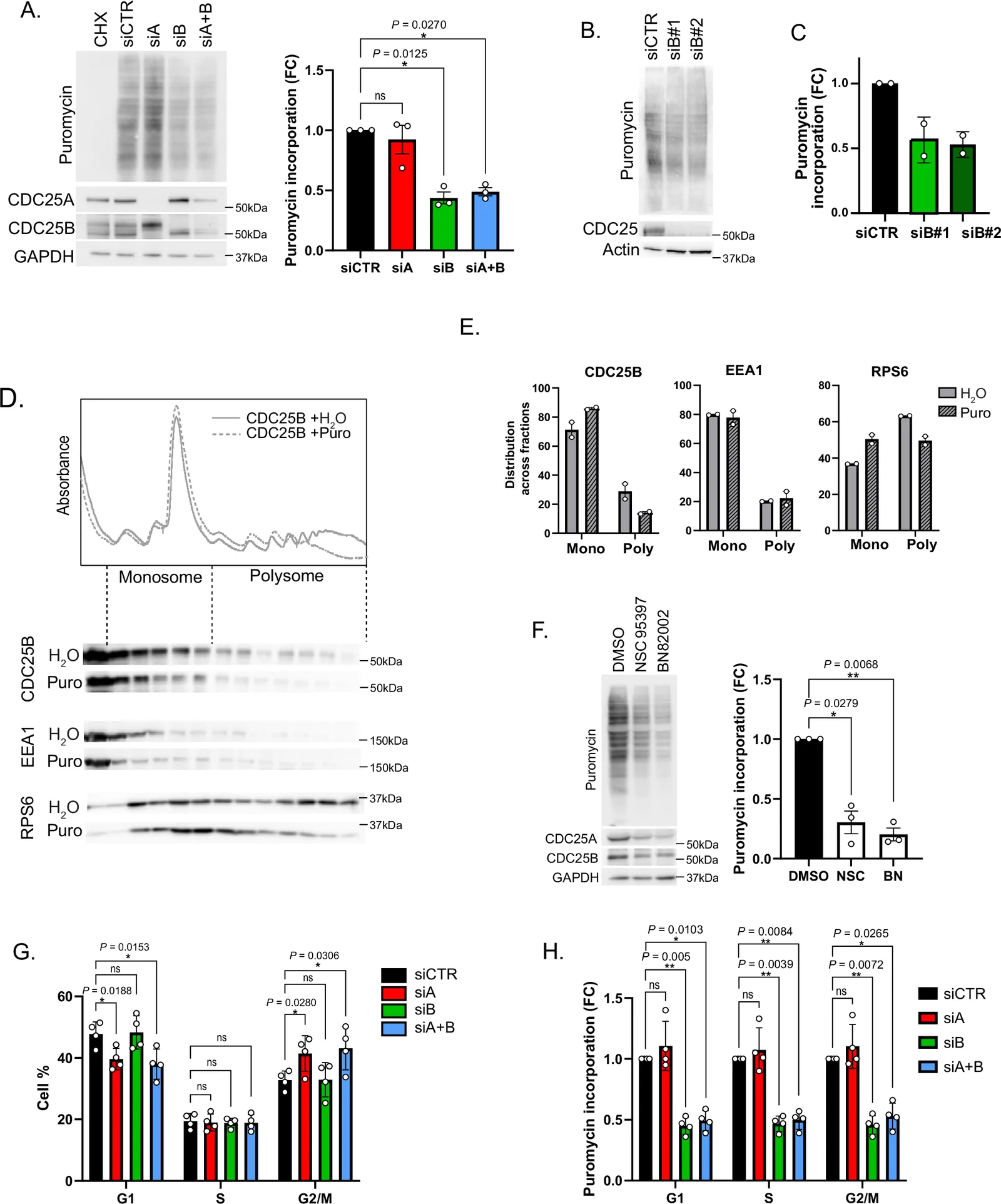
Compensation between CDC25A and CDC25B. (**A**) Western blot analysis (left panel) and quantification (right panel) of puromycin incorporation, and of CDC25A and CDC25B protein levels in response to siRNA-mediated depletion of CDC25A (siA) and/or CDC25B (siB) in HEK293 cells. Cycloheximide treatment (CHX) was used as a positive control of translation inhibition. Data are presented as mean values ± SEM of *n* = 3 or independent experiments, *P < 0.05, ns: non-significant (two-way ANOVA). (**B**, **C**) Western blot analysis (**B**) and quantification (**C**) of puromycin incorporation, and of CDC25B protein levels in response to siRNA-mediated depletion of CDC25B using two distinct siRNA (siB#1 and siB#2) in HEK293 cells. Data are presented as mean values ± SEM of *n* = 2 independent experiments. (**D**) Polysome sedimentation profile of HEK293 cells transfected with CDC25B for 48 h (upper panel), and western blot analysis of CDC25B in the monosomal and polysomal fractions (lower panel), in the absence (H_2_O) or the presence of puromycin (Puro). Western blots of EEA1 and RPS6 were used as negative and positive controls, respectively. Shown is a representative result from *n* = 3 independent experiments. (**E**) Quantification of CDC25B, EEA1, and RPS6 western blots in monosomal (Mono) and polysomal (Poly) fractions in the presence (Puro) or the absence (H_2_O) of puromycin. Data are presented as mean values ± SEM of *n* = 3 independent experiments, *P < 0.05; **P < 0.01, ns: non-significant (repeated measures two-way ANOVA). (**F**) Western blot analysis of puromycin incorporation in HEK293 cells treated for 1 h with CDC25 inhibitors NSC95397 (30 μM) and BN8202 (100 μM). Data are presented as mean values ± SEM of *n* = 3 independent experiments, *P < 0.05, **P < 0.01 (two-way ANOVA). (**G**) Flow cytometry cell cycle analysis (DAPI labeling) of HEK293 cells in response to CDC25A (siA), CDC25B (siB), or CDC25A and CDC25B (siA+B) depletion. Data are presented as mean values ± SEM of *n* = 4 independent experiments, *P < 0.05, ns: non-significant (two-way ANOVA). (**H**) Flow cytometry analysis of puromycin incorporation in response to CDC25A (siA) and/or CDC25B (siB, siA+B) depletion during G1, S, and G2/M phases of the cell cycle. Data are presented as mean values ± SEM of *n* = 4 independent experiments, *P < 0.05, **P < 0.01, ns: non-significant (two-way ANOVA). Source data are available online for this figure.

We then investigated the impact of CDC25A silencing on mRNA translation in HEK293 cells. Surprisingly, siRNA-mediated CDC25A downregulation did not modify the sunset profile (Fig. 3A,B). The observation that CDC25B expression was increased in response to CDC25A downregulation (Fig. 3C,D), raised the hypothesis that CDC25B could compensate for the effects of CDC25A downregulation on mRNA translation. In agreement with this hypothesis, we observed that siRNA-mediated CDC25B depletion significantly decreased translation (Figs. 3B and EV3A), without modifying CDC25A level (Fig. 3C,E). This effect on translation was validated using a second independent siRNA targeting CDC25B (Fig. EV3B,C).

To further confirm a possible implication of CDC25B in the translation regulation, we performed polysome profiling of HEK293 cells transfected with CDC25B and investigated the presence of this protein in the polysomal fractions, as performed for CDC25A in Fig. 1D. As shown in Fig. EV3D,E, CDC25B is localized in the polysomal fractions, and its association depended on polysome integrity. Double CDC25A and CDC25B depletion induced a similar decrease compared to CDC25B alone (Figs. 3B and EV3A), further suggesting that modifications of CDC25B expression might interfere with the response to CDC25A depletion. Similarly, short-term (1 h) pharmacological inhibition of CDC25 phosphatases, using NSC95397 and BN82002, which are not discriminant between CDC25A and B activities, induced a quick and robust drop of translation (Fig. EV3F), suggesting that both phosphatases are probably involved in this process, and that CDC25B probably upregulates translation in the absence of CDC25A.

To define whether these compensatory mechanisms of translation might involve cell cycle regulations, we performed cell cycle analysis by FACS. CDC25A depletion for 24 h induced a modest reduction of cells in G1 and a consequent accumulation in G2/M, while CDC25B depletion had no significant effect (Fig. EV3G). Importantly, the impact of CDC25B depletion on translation was similar in all phases of the cell cycle, including in G1 (Fig. EV3H), an interesting observation when considering that, contrary to CDC25A, CDC25B cell cycle functions are essentially restricted to CDK2 and CDK1 activation during the S and G2 phases.

We then asked whether CDC25B activity could impact the translation induced by CDC25A overexpression (Fig. 2). First, we observed that overexpression of CDC25A decreased CDC25B expression (Fig. 3F), meaning that the effect of CDC25A overexpression on translation is not mediated by CDC25B. Moreover, the increase of translation efficiency induced by CDC25A overexpression is similar when CDC25B is depleted by RNA interference (Fig. 3G), further suggesting that CDC25A and B act independently of each other.

Altogether, these data suggest that both CDC25A and CDC25B regulate mRNA translation all along the cell cycle, independently of their role in CDK activation, and that CDC25B can compensate for CDC25A depletion in asynchronous cells but does not interfere with the effects of CDC25A overexpression.

### CDC25A and CDC25B regulate translation elongation and initiation, respectively

**Figure 4:**
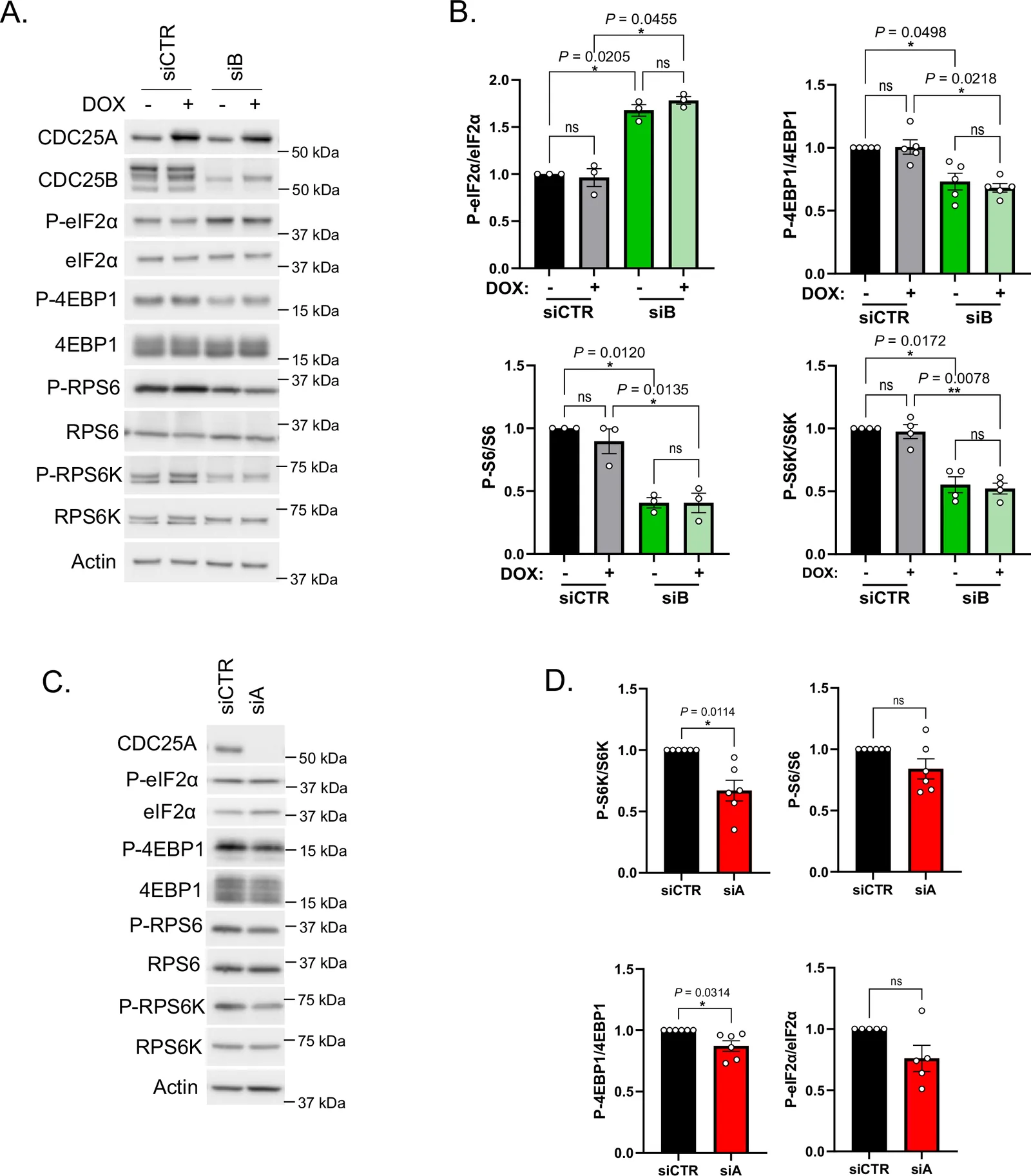
CDC25B regulates translation initiation. (**A**, **B**) Western blot analysis (**A**) and quantification (**B**) of translation initiation regulators phosphorylation in response to CDC25A induction (DOX) and/or CDC25B deletion (siB). For (**B**), data are presented as mean values ± SEM of *n* = 3–5 independent experiments, *P < 0.05; **P < 0.01; ns: non-significant (repeated measures two-way ANOVA). (**C**, **D**) Western blot analysis (**C**) and quantification (**D**) of translation initiation actors phosphorylation in response to CDC25A depletion (siA). For (**D**), data are presented as mean values ± SEM of *n* = 5 independent experiments, *P < 0.05, ns: non-significant (two-sided paired *t* test). Source data are available online for this figure.

**Figure EV4:**
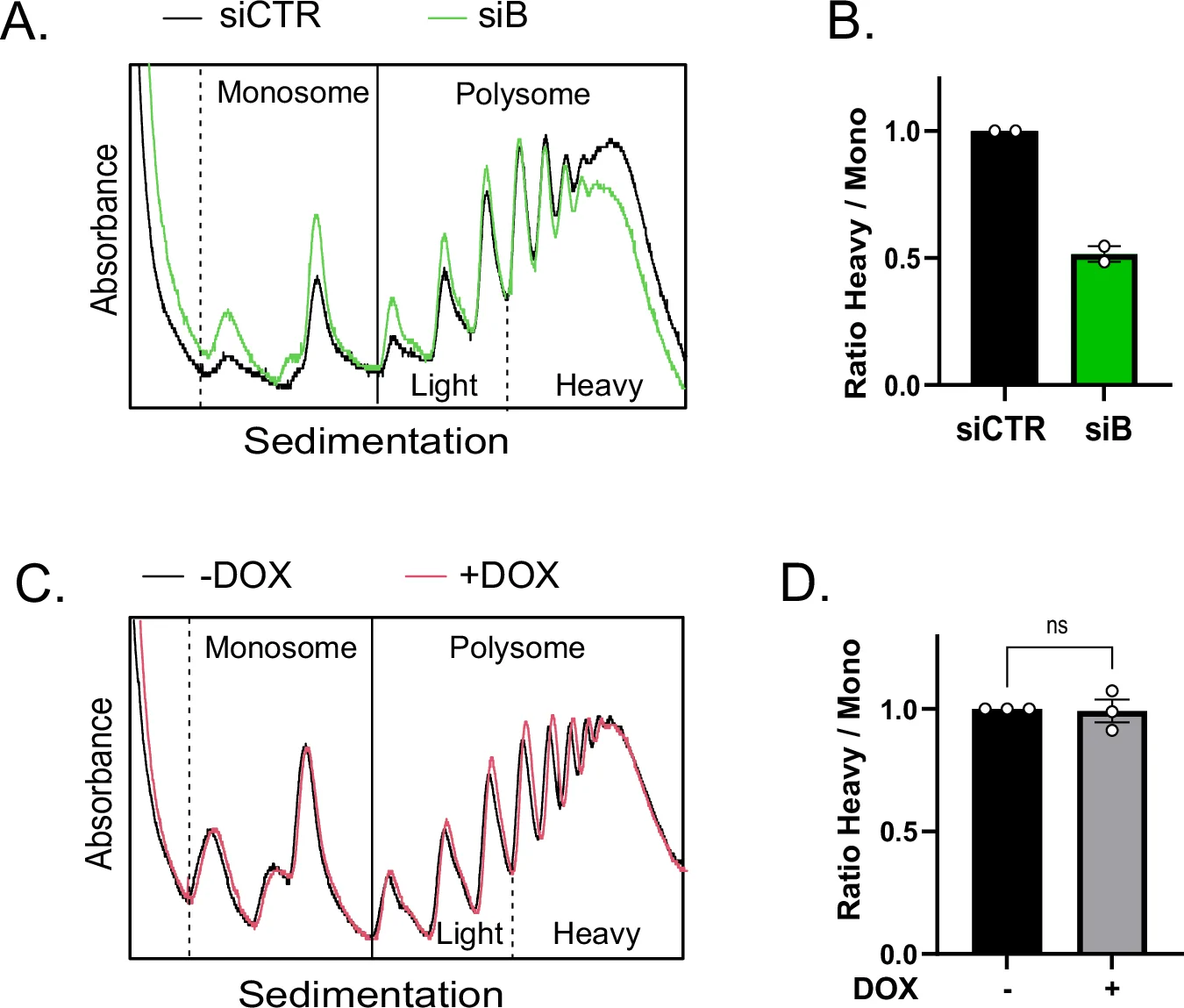
CDC25B knockdown affects polysome profiling. (**A**–**D**) Polysome sedimentation profile of HEK293 after CDC25B depletion (siB) (**A**, **B**), and of non-induced HEK293 cells (-DOX) or induced for HA-CDC25A expression ( + DOX) (**C**, **D**). Shown is a representative result from *n* = 2 or 3 independent experiments. (**B**, **D**) Quantification of the ratio between the area under the Polysome curve and the Monosome curve from the polysome profile in (**A**, **C**). Data are presented as mean values ± SEM of *n* = 2 or 3 independent experiments, ns: non-significant (two-sided paired *t* test). Source data are available online for this figure.

To better understand the implications of CDC25B in translation, we examined the effect of its depletion on major signaling pathways involved in translation initiation in HEK293 cells. As shown in Fig. 4A, most of these pathways, including mTOR and eIF2α, were altered by CDC25B downregulation (Fig. 4A, lane 1 vs 3, Fig. 4B). By contrast, siRNA-mediated depletion of CDC25A had a more limited effect on mTOR and eIF2α pathways (Fig. 4C,D) that might be explained by the compensatory role of CDC25B after CDC25A depletion. Moreover, doxycycline-induced overexpression of CDC25A neither modified the effects of CDC25B on the translation initiation signaling pathways (Fig. 4A, lane 1 vs 2, Fig. 4B), nor rescued the effect of CDC25B depletion (Fig. 4A, lane 3 vs 4, Fig. 4B), indicating a specific role for CDC25B in this translation initiation. Consistent with a role of CDC25B in regulating translation initiation, siRNA-mediated downregulation of CDC25B led to a marked reduction in polysome assembly that correlates with an accumulation of 80S particles (Fig. EV4A,B). These results, together with the data showing that CDC25A overexpression increased global protein synthesis as measured by SUnSET (Fig. 2A) but did not modify polysome profiles (Fig. EV4C,D), suggest that, in contrast to CDC25B, CDC25 A may preferentially regulate translation elongation rather than initiation.

**Figure EV5:**
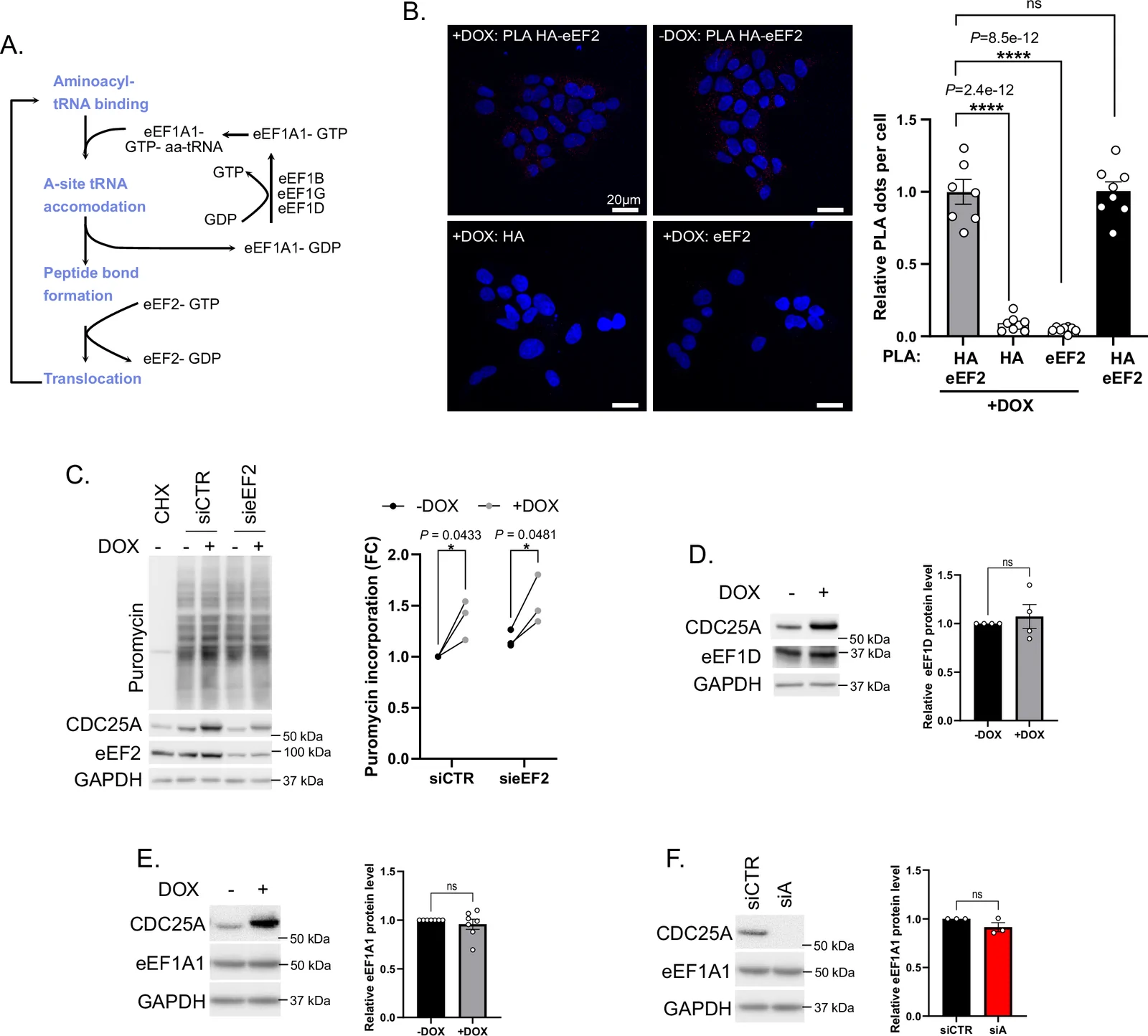
The variation of CDC25A expression affects translation independently of eEF2. (**A**) Scheme of the translation elongation cycle. eEF1A1 delivers the aa-tRNA to the A-site of the ribosome. After peptide bond formation, the ribosome is translocated with the help of eEF2. The GTP exchange for eEF1A1 is a complex composed of eEF1B, eEF1G, and eEF1D. (**B**) Immunofluorescence analysis (left panel) and quantification (right panel) of proximity ligation assay experiments performed in HEK293 cells induced for HA-CDC25A expression (DOX) with HA and eEF2 antibody. Negative controls were performed with either eEF2 or HA antibodies alone, or in non-induced cells that do not express HA-CDC25A. Data are presented as mean values ± SEM of *n* = 2 independent experiments (4 images were acquired per experiment; each dot is an image), ****P < 1.e-11, ns: non-significant (two-way ANOVA). (**C**) Western blot analysis (left panel) and quantification (right panel) of puromycin incorporation in response to CDC25A induction (DOX) and/or translation elongation factors eEF2 depletion (sieEF2). Data are presented as mean values ± SEM of *n* = 3 independent experiments, *P < 0.05 (two-way ANOVA). (**D**–**F**) Western blot analysis and quantification of eEF1D (**D**) or eEF1A1 (**E**, **F**) protein level in response to CDC25A induction (DOX) or depletion (siA). Data are presented as mean values ± SEM of *n* = 3–7 independent experiments, ns: non-significant (two-sided paired *t* test). Source data are available online for this figure.

**Figure 5:**
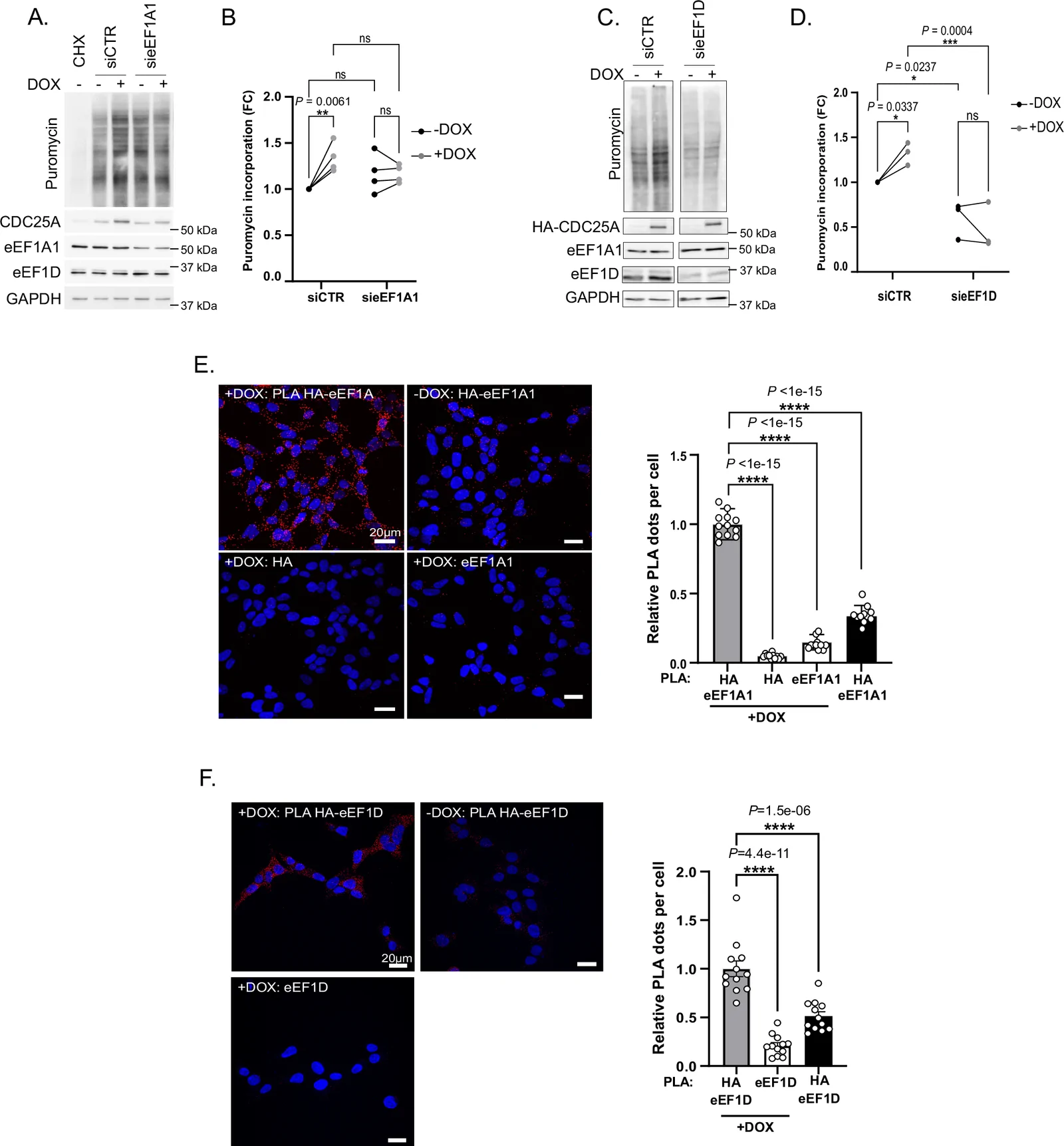
CDC25A regulates translation elongation. (**A**–**D**) Western blot analysis (**A**, **C**) and quantification (**B**, **D**) of puromycin incorporation in response to CDC25A induction (DOX) and/or translation elongation factors eEF1A1 (**A**, **B**) and eEF1D (**C**, **D**) depletion for 24 h or 48 h, respectively. For (**B**, **D**), data are presented as mean values ± SEM of *n* = 4 independent experiments, *P < 0.05; **P < 0.01; ***P < 0.0001; ns: non-significant (two-way ANOVA). (**E**,** F**) Immunofluorescence analysis (left panel) and quantification (right panel) of proximity ligation assay experiments performed in HEK293 cells induced for HA-CDC25A expression (DOX) with HA and eEF1A1 or eEF1D antibodies. Negative controls were performed with either eEF1A1, eEF1D, or HA antibodies alone, or in non-induced cells that do not express HA-CDC25A (-DOX). Data are presented as mean values ± SEM of *n* = 3 independent experiments (4 images were acquired per experiment; each dot is an image), ****P < 1.e-15 (two-way ANOVA). Source data are available online for this figure.

To investigate the molecular mechanism underlying the role of CDC25A in translation, we focused on elongation factors previously reported to interact with CDC25A, particularly eEF1A1 (Wang et al, 2011) and its partner eEF1D, a component of the eEF1B complex that cooperates with eEF1A1 during translation elongation (Xu et al, 2021) (Fig. EV5A). As shown in Fig. 5A,B, depletion of eEF1A1 abolished the increase in translation induced by CDC25A overexpression, suggesting that eEF1A1 is required for this effect. Consistent with the functional cooperation between eEF1A1 and eEF1D in translation, prolonged depletion of eEF1D produced a similar effect, abrogating the response to CDC25A overexpression and markedly reducing global protein synthesis (Fig. 5C,D). To further substantiate the functional relationship between eEF1A1, eEF1D, and CDC25A, we investigated whether these factors interact *in cellulo* by performing proximity ligation assay (PLA) using antibodies against endogenous eEF1A1 or eEF1D and the ectopically expressed, doxycycline-inducible HA-tagged CDC25A. The number of PLA dots in the cytoplasm detected using the HA antibody in combination with eEF1A1 or eEF1D antibodies was significantly higher than the number detected in the absence of doxycycline-induced CDC25A or with each PLA antibody alone (Fig. 5E,F), indicating a specific interaction between CDC25A and these elongation factors. By contrast, PLA experiments examining the interaction between CDC25A and eEF2, another major elongation factor that functions downstream of eEF1A1, did not show co-localization of these two proteins (Fig. EV5B). This is consistent with the inability of eEF2 to modulate the increase in translation induced by CDC25A (Fig. EV5C). Altogether, these data suggest that CDC25A regulates translation elongation by modulating the activity of eEF1A1 and/or eEF1D. Nonetheless, CDC25A overexpression or depletion did not alter the protein levels of eEF1A1 or eEF1D (Fig. EV5D–F), suggesting that CDC25A might regulate eEF1A1 and/or eEF1D through post-translational modifications.

### CDC25A regulates translation in AML cells

**Figure 6:**
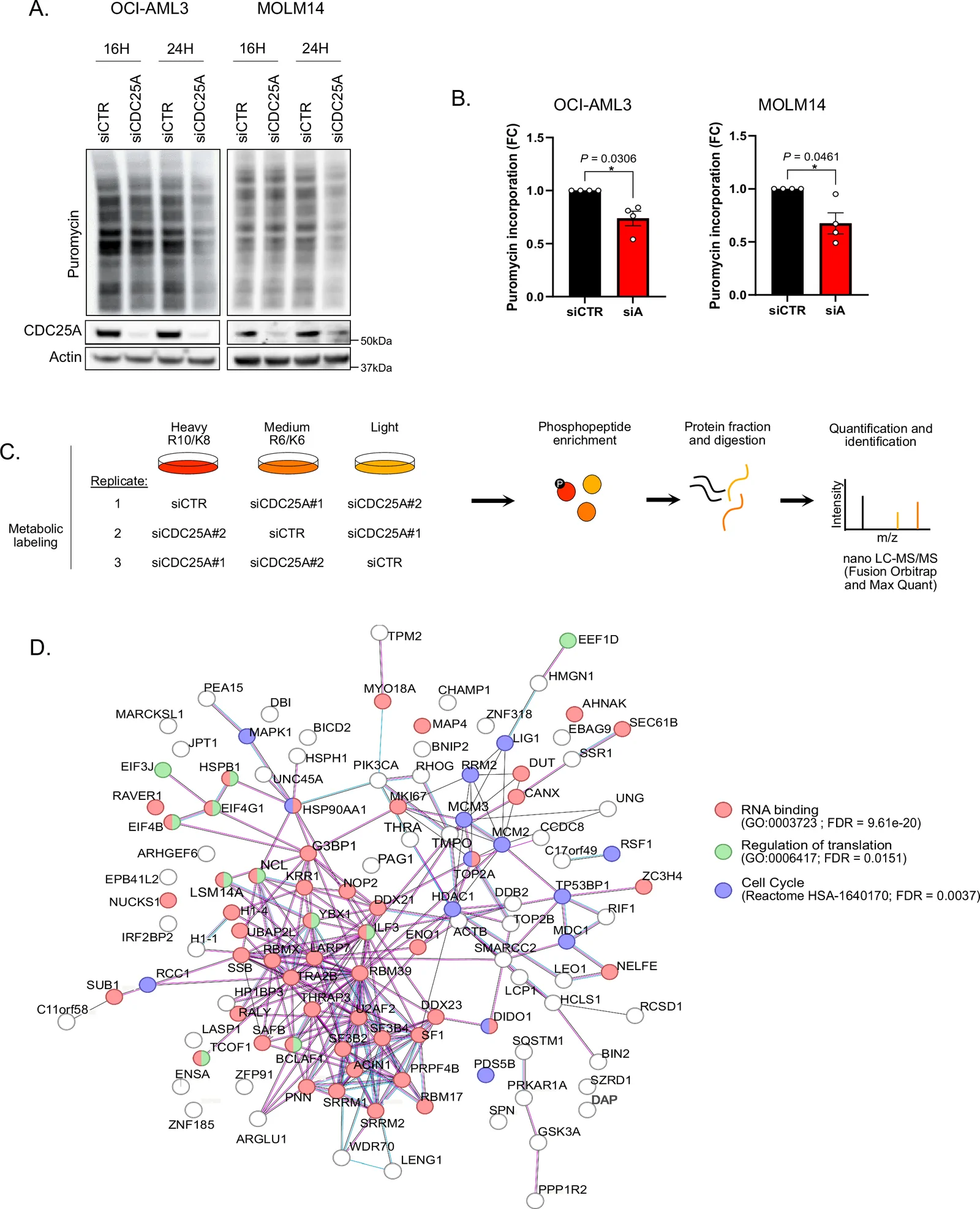
Translation factors are regulated by CDC25A phosphatase in leukemic cells. (**A**, **B**) Western blot analysis (**A**) and quantification (**B**) of puromycin incorporation and CDC25A protein level in response to siRNA-mediated depletion of CDC25A in leukemic MOLM-14 and OCI-AML3 cells. For (**B**), data are presented as mean values ± SEM of *n* = 4 independent experiments, *P < 0.05 (two-sided paired *t* test). (**C**) SILAC quantitative phosphoproteomic analysis protocol on extracts from MOLM-14 after siRNA-mediated depletion of CDC25A. (**D**) Network of interactions, generated with STRING database (Szklarczyk et al, 2021), between the proteins whose phosphorylation is impacted by siRNA-mediated depletion of CDC25A. Source data are available online for this figure.

**Figure EV6:**
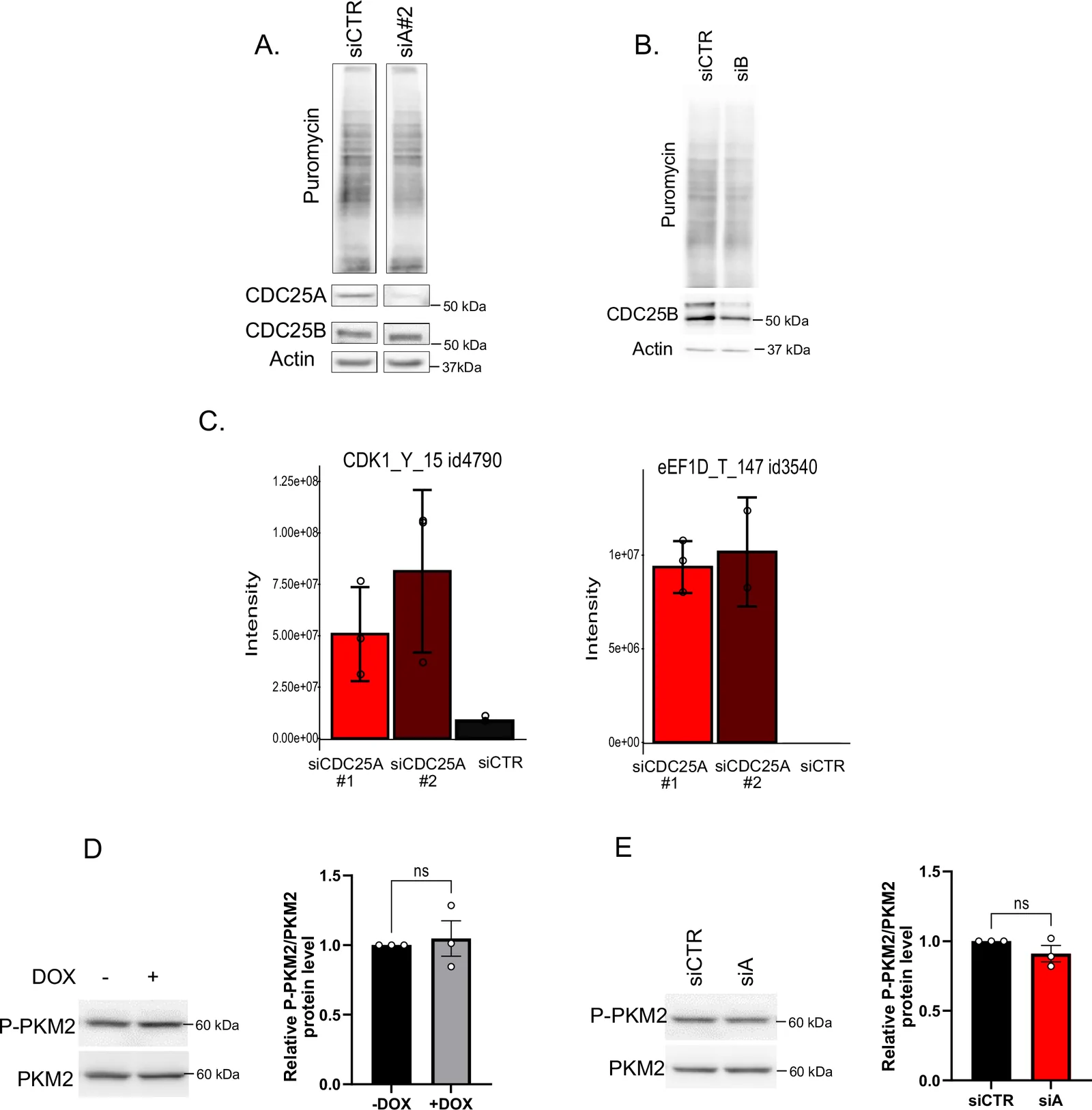
CDC25A controls CDK1, eEF1D phosphorylation but does not regulate PKM2 phosphorylation. (**A**) Western blot analysis of puromycin incorporation and of CDC25B protein levels in response to siRNA-mediated depletion of CDC25A using a distinct siRNA (siA#2) in OCI-AML3 cells. (**B**) Western blot analysis of puromycin incorporation in response to siRNA-mediated depletion of CDC25B using a distinct siRNA in OCI-AML3 cells. (**C**) LFQ intensity, identified by mass spectrometry following the SILAC quantitative phosphoproteomic analysis (Fig. 5C, *n* = 3), for CDK1 Tyr15 (Y_15) and eEF1D Thr147 (T_147) in MOLM-14 treated with siRNA control (CTR) or siRNAs against CDC25A (siCDC25A #1 or #2). (**D**, **E**) Western blot analysis of PKM2 and PKM2 phosphorylation (P-PKM2) in response to doxycycline-induced CDC25A overexpression (**D**) or siRNA-mediated depletion (**E**) in HEK293 cells. Data are presented as mean values ± SEM of *n* = 3 independent experiments, ns: non-significant (two-sided paired *t* test). Source data are available online for this figure.

In previous works, we identified CDC25A as an essential factor driving the proliferation and differentiation of acute myeloid leukemia cells (Fernandez-Vidal et al, 2006; Bertoli et al, 2015; Sueur et al, 2020). Taking advantage of this dependance to CDC25A activity, we tested the impact of CDC25A depletion on translation by performing puromycin incorporation assay (SUnSET) in AML cell lines. By contrast with HEK293 cells, we observed that RNA interference-mediated CDC25A depletion reduced global mRNA translation in MOLM-14 and OCI-AML3 leukemic cell lines (Figs. 6A,B and  EV6A, using a second independent siRNA targeting CDC25A). In these cells, CDC25B does not compensate for CDC25A depletion (Fig. EV6A), although CDC25B depletion inhibits translation (Fig. EV6B). To better understand the effect of CDC25A depletion on global mRNA translation, we aimed at identifying CDC25A phosphorylation protein targets. To this end, we depleted CDC25A using two specific CDC25A siRNAs in MOLM-14 leukemic cells, and we performed a SILAC quantitative phosphoproteomic study followed by mass spectrometry analysis (Fig. 6C). Consistent with its well described canonical function, a protein-protein network of the phosphorylated proteins using STRING database (Szklarczyk et al, 2021) showed that CDC25A significantly regulates the phosphorylation of proteins associated to the cell cycle process (Fig. 6D). In agreement with this observation, the well-characterized CDC25A target site at the CDK1 Tyr15 showed an upregulated phosphorylation in response to CDC25A depletion (Fig. EV6C; Dataset EV2). In agreement with the impact of siRNA-mediated CDC25A depletion on global mRNA translation (Fig. 6A), proteins involved in mRNA translation, like initiation and elongation factors, as well as RBPs involved in translation, showed a significant increase in phosphorylation following CDC25A depletion (Fig. 6D; Dataset EV2). Interestingly, we observed that phosphorylation of the elongation factor eEF1D on Thr147 was increased (Fig. EV6C; Dataset EV2).

## Discussion

In this work, we demonstrate that CDC25A and CDC25B regulate mRNA translation independently of their CDK-activating function. Since CDC25A and B are dual-specificity phosphatases, their activating function on translation may occur through dephosphorylation of an inhibitory site on a positive translational regulator, or of an activating site on a negative translational regulator. Our phosphoproteomic screen identified several potential candidates, and we demonstrated both a functional link and a co-localization of CDC25A with the elongation factors eEF1A1 and eEF1D. Supporting this interplay, Lin et al (Lin et al, 2010) found that eEF1A1 translational function is negatively regulated by phosphorylation on the Ser300 residue, downstream of TGFβ signaling. Further investigation of this interaction will necessitate a phospho-specific antibody to elucidate the role of CDC25A in the dephosphorylation of this site and the activation of eEF1A1. In addition, our phosphoproteomic analysis identified the elongation factor eEF1D as a potential CDC25A substrate. Indeed, Thr147 phosphorylation of eEF1D was increased in response to CDC25A inhibition (Dataset EV2). eEF1D (eEF1Bδ) is a subunit of the eEF1B complex, which works in association with eEF1A to regulate elongation (Xu et al, 2021). Thr147 of eEF1D was found phosphorylated in several large-scale phospho-proteomic studies, in particular in response to genotoxic treatments or oncogenic signaling inhibition (see PhosphositePlus site (Hornbeck et al, 2015)). However, the functional impact as well as the regulation of this phosphorylation remain unknown. Since CDC25A inhibition modulates this phosphorylation, this opens the possibility that either or both eEF1D and eEF1A1 regulate elongation downstream of CDC25A. Whether these two elongation factors are direct substrates of CDC25A remains to be established.

PKM2 is one of the very few described targets of CDC25A, apart from CDKs (Liang et al, 2016). Interestingly, recent works pointed to ribosomal and mRNA regulation functions of PKM2 (Kejiou et al, 2023). Although PKM2 was not detected in our CDC25A phosphoproteome analysis, we investigated whether it could be involved in CDC25A-dependent regulation of translation. For this, we estimated the impact of CDC25A expression or depletion on PKM2 level and Ser37 phosphorylation. As shown in Fig. EV6D,E, neither overexpression nor siRNA-mediated depletion of CDC25A modified PKM2 level or Ser37 phosphorylation status, indicating that CDC25A does not regulate translation through PKM2.

Importantly, the combined analysis of translation (using both polysomal profile and sunset assays) and signaling pathways reveals that CDC25A and CDC25B regulate translation elongation and initiation, respectively. The impact of CDC25B depletion on the mTOR pathway likely reflects an indirect function of the phosphatase, as we observed dephosphorylation of 4EBP1, RPS6, and RPS6K under these conditions (Fig. 4A,B). By contrast, the effect on the eIF2α Ser51 phosphorylation could be direct, although the GADD34 phosphatase is considered the major regulator of this dephosphorylation. eIF2 belongs to the 43S pre-initiation complex recruited to mRNA at the initiation steps of translation, implicating potential localization of this protein and of its regulators in light or heavy polysomal fractions. Further experiments are needed to determine whether CDC25B can either replace or complement GADD34 function under specific conditions. In addition, we cannot exclude that other, as yet unidentified, targets of CDC25B may contribute to its localization within polysomal fractions.

Our results also suggest that CDC25B can compensate for CDC25A depletion. Given that CDC25B regulates key steps in translation initiation, this likely explains why the effects of CDC25A depletion are not easily detectable in our experiments. To our knowledge, increased expression of CDC25B in response to CDC25A downregulation has not been reported in the literature. Murine knock-out of the two genes rather argues for CDC25A compensating for the absence of CDC25B and CDC25C during development (Ferguson et al, 2005). However, our observation that siRNA-mediated CDC25A depletion induces G2 accumulation of HEK293 cells (Fig. EV3G) may explain this compensation and the increase of CDC25B expression observed in these conditions. Indeed, major CDC25B functions have been identified at the S/G2 and G2/M transitions, and expression of the protein is increased during these phases of the cell cycle (Boutros et al, 2007). Although its more divergent sequence homology with CDC25A and CDC25B, as well as its exclusive function during mitosis, do not argue for CDC25C involvement in translation, we cannot exclude its implication in this process in specific circumstances.

Altogether, our data suggest that CDC25A and CDC25B work as molecular platforms coordinating cell cycle progression with mRNA translation and protein synthesis. Given their specific CDK-dependent functions at different phases of the cell cycle, one hypothesis could be that each phosphatase regulates translation at specific phases. However, flow cytometry analysis of puromycin incorporation demonstrates that both CDC25A and CDC25B can regulate translation similarly throughout cell cycle progression, including during G1. Together with the results shown in Fig. 4, this rather argues for distinct functions of CDC25B and CDC25A in translation initiation and elongation, respectively, independently of their canonical functions in cell-cycle control, consistent with potential moonlighting activities of CDC25 phosphatases. Overall, these results expand the functional landscape of CDC25 phosphatases and suggest that their non-canonical roles in translational control may represent an additional vulnerability in proliferative diseases.

## Methods

**Table:** Reagents and tools table

Reagent/resource	Reference or source	Catalog number
**Experimental models**		
MOLM-14 (*H. sapiens*)	DSMZ	ACC 777
OCI-AML3 (*H. sapiens*)	DSMZ	ACC 582
HEK-293 (*H. sapiens*)	ATCC	CRL-1573
**Recombinant DNA**		
pIRES2-GFP-CDC25Awt	Gifted by Dr Chritsine Dozier, CBI, Toulouse, France.	
pIRES2-GFP-CDC25AC431S	Gifted by Dr Chritsine Dozier, CBI, Toulouse, France.	
pCDNA-HA-CDC25B2	Gifted by Dr Chritsine Dozier, CBI, Toulouse, France.	
pCDNA-HA-CDC25B3	Dr Odile Mondesert, CBI, Toulouse, France	
pIRES2-EGFP	Clontech, Mountain View, CA	
pTRE-tight	Clontech, Mountain View, CA	
pZDonor-AAVS1	Sigma-Aldrich, St Louis, MO, USA	
**Antibodies**		
PTM Scan Phospho-Tyrosine Rabbit mAb	Cell Signaling P-Tyr-1000 Kit	8803
Rabbit IgG	Millipore	12-370
Rabbit HA antibody	CST	3724, 1/50
CDC25A	Santa Cruz	sc-7389
CDC25B	Santa Cruz	sc-326
GAPDH	Santa Cruz	sc-32233
Puromycin	Millipore	MABE343
p4EBP	Cell Signaling Technologies	9456 P
4EBP	Cell Signaling Technologies	9644S
pS6K	Cell Signaling Technologies	9234 P
S6K	Cell Signaling Technologies	2708
Actin	Chemi-Con	MAB1501
peIF2a	Cell Signaling Technologies	3398S
eIF2a	Cell Signaling Technologies	3398S
EEA1	Santa Cruz	sc-53939
CDK2	Santa Cruz	sc-163
RPS6	Santa Cruz	sc-74459
P-RPS6	Cell Signaling	2215S
eEF1D	Proteintech	PR-60085
eEF2	Bethyl	BE-A301-688A-T
PKM2	Cell Signaling	1398
P-PKM2	Invitrogen	PA5-37684
Anti-puromycin antibody coupled with Alexa 647	BioLegend	381508
HA	Cell Signaling Technologies	3724
HA	Sigma	A2095
eEF1A1	Proteintech	67495
HA	Sigma	H9658
Tubulin	Santa Cruz	sc-5286
HRP-conjugated anti-rabbit	Ozyme	7074S
or HRP-conjugated anti-mouse	Ozyme	7076S
**Chemicals, enzymes, and other reagents**		
RPMI 1640	Life Technologies	61870044
Fetal bovine serum	Life Technologies	A5256701
Penicillin–streptomycin	Sigma	P0781
BN82002	Abcam	Ab287024
NSC9597	Sigma	N1786
Palbociclib	Bio-techne	4786/10
Roscovitine	Bio-techne	1332/10
Ro-3306	Bio-techne	4181/10
Puromycin	Euromedex	UP9200-A
Doxycycline	Sigma	D9891
Lipofectamine RNAiMAX	Life Technologies	13778150
Lipofectamine 3000	Fisher Scientific	15292465
1× Laemmli	Bio-Rad	1610747
BCA	Interchim	UP40840A
DAPI	Sigma	P36931
RNaseOUT	Invitrogen	10777019
**Software**		
Maxquant software version 1.6.17.0		
Perseus software version 1.6.14.0		
DIA-NN version 1.9		
FlowJo v10		
**Other**		
Amaxa nucleofection system	Lonza, Koeln, Germany	
Invitrogen Neon transfection system	Invitrogen™ MPK5000	
**IMAC beads**	**Phos-select Iron Affinity gel, Sigma**	**P9740**
TiO2 beads	GlSciences	5020-75000
C8 stage-tip	3 M Empore Dics	2214-C8
Orbitrap Fusion Mass Spectrometer MS	Thermo Scientific	
Protein A/G magnetic beads	ThermoFisher	88802
S-Trap Micro Spin Column	Protifi, NY, USA	
Detergent Removal Spin Columns	Pierce™	N°87777
Dionex U3000 RSLC nano-LC- system coupled to a TIMS-TOF Pro mass spectrometer	Bruker Daltonik GmbH, Bremen, Germany	
C_18_ reverse-phase column	Thermo Fisher Scientific	
Aurora C18 reverse-phase resin	IonOpticks, Middle Camberwell, Australia	
ECL system	Amersham Pharmacia Biotech	
Ibidi µ-slides 8 wells	Cliniscience	80827
Duolink kit	Sigma Duolink In Situ orange	DUO92102
ISCO density gradient fractionation system	Foxy Jr fraction collector coupled to UA-6UV detector, Lincoln, NE	
**Oligonucleotides**	**Source**	**Sequence or catalog number**
siRNA Control	This study	5’-GGUCCGGCUCCCCCAAAUGdTdT-3’
siRNA CDC25A#1	This study	5’-AAGGGTTATCTCTTTCATACAdtdt-3’
siRNA CDC25A#2	This study	5’- CTGTGATATTGATAGCTTTdtdt-3’
siRNA CDC25B#1	This study	5’-AGACUGCAGAUACCCCUAUdTdT-3’
siRNA CDC25B#2	This study	5’- AGACTGCAGATACCCCTATdtdt-3’
siRNA eEF2	This study	5’-GGUGUUUGAUGCGAUCAUGAAUUUC dTdT-3’
siRNA eEF1A1	This study	5’-CCGGUAUGGUGGUCACCUU dTdT-3’
siRNA eEF1D	This study	5’- TGACGAGGATGATGACA dTdT-3’
Total CDC25A siRNA	Qiagen, Hilden, Germany	Hs_CDC25A_9,
3′UTR CDC25A siRNA	Sigma	CDC25A 2943
siRNA negative control	Dharmacon	ON-TARGETplus control pool

### Cell lines and reagents

MOLM-14 and OCI-AML3 leukemic cell lines were grown in RPMI 1640 medium supplemented with 10% Fetal Bovine Serum and 1× penicillin–streptomycin. HEK293-CDC25A cell lines were grown in DMEM medium supplemented with 10% calf serum and 1× penicillin–streptomycin. All cells were cultured at 37 °C and 5% CO_2_, in a humidified incubator, and were regularly tested for mycoplasma contamination.

### Establishment of HEK293 inducible CDC25A cell lines

HEK293 cell lines were designed for conditional expression of HA-tagged CDC25A as described in (Mazzolini et al, 2016). Briefly, the bicistronic Cdc25A expression plasmids were constructed by inserting the coding sequence of human Cdc25A into the pIRES2-EGFP vector. The R446L mutation was introduced by site-directed mutagenesis using inverted PCR. The TET-Cdc25A vectors were constructed by first inserting the coding sequence of human Cdc25A downstream of the Tet promoter in the pTRE-tight plasmid. This TET cassette was then introduced in the pZDonor-AAVS1 plasmid) together with the tricistronic expression cassette of vector pWHE644, which allows the constitutive expression of Tet activator, Tet repressor, and puromycin resistance genes. Tetracycline-inducible HEK293 cell pools were generated by a one-step transformation process with the “all-in-one” TET-Cdc25A plasmid vectors. Plasmids were transfected with the Amaxa nucleofection system. Puromycin-resistant pools were selected in culture medium supplemented with 1 μg/ml puromycin. Cells were induced for CDC25A expression with 1 μg/ml of doxycycline for 24 h.

### Cell transfection

siRNAs were transfected using the Lipofectamine RNAiMAX according to the manufacturer’s instructions. Cells were reverse-transfected with 20 nM siRNA in HEK293 for 24 h. siRNA were transfected into MOLM14 and OCI-AML3 by electroporation through the Invitrogen Neon transfection system. All used primers can be found in the Reagents and Tools table. Cells were incubated for 24 h at 37 °C before harvesting and analysis.

Plasmids were transfected using Lipofectamine 3000 according to the manufacturer’s instructions. In brief, cells were transfected the day after plating with 0.5 µg DNA in six wells for 48 h.

### SILAC labeling

For SILAC metabolic labeling, MOLM-14 cells were grown in SILAC-RPMI lysine and arginine-deficient medium (Pierce), supplemented with 10% dialyzed fetal calf serum (Pierce), and complemented with heavy (arginine-13C6-15N4 (Arg10), lysine-13C6-15N2 (lys8)), medium (arginine-13C6 (Arg6), lysine-13C6 (Lys6)) (medium and heavy amino acids from Cambridge Isotope Laboratories), or light (Sigma) amino acids for 10 days. The complete incorporation of heavy labeled amino acids in proteins was tested by mass spectrometry and obtained after 5 passages. Once labeling was completed, cells were transfected with either total CDC25A siRNA (Hs_CDC25A_9) or 3′UTR CDC25A siRNA (CDC25A 2943) or siRNA negative control.

### Phosphoproteomics

#### Sample preparation

Cells were lysed in a buffer containing 2% Sodium Deoxycholate, 10 mM TCEP, 40 mM CAA, 100 mM Tris then boiled 5 min at 95 °C. Samples were sonicated by diagenode during 45 min before being boiled again 5 min at 95 °C. MgCl_2_ (1 mM final) and 1 µl of benzonase were added, then samples were incubated 1 h at room temperature. In total, 4 ml of cold acetone was added, and samples were placed at −20 °C overnight. After cold centrifugation for 15 min at 10,000× *g*, washing with 2 * 1 ml of cold acetone, the pellet was dried and solubilized in 1 ml of TEAB 100 mM-TFE 10%. Samples were digested overnight with trypsin 1/50 and LysC 1/250. Peptides were then speed-vacuum dried, and phospho-tyrosine phosphorylated peptides were immunoaffinity purified using the PTM Scan Phospho-Tyrosine Rabbit mAb, following the protocol provided by the kit.

TFA was added to the unbound peptides to reach 2% final, then peptides were cleaned up at room temperature on Sep-Pak columns (C18 from Waters Corporation WAT036905). The column was equilibrated and washed successively with ACN, 50% ACN–0.1% TFA, and 0.1% TFA. Once the peptides are attached, they are washed with 0.1% TFA and eluted three times with 80% ACN–0.5% TFA before being freeze-dried. IMAC beads were washed three times in a solution containing 1% FA–40% ACN before being incubated for 1 h at room temperature with the peptides (20 µl of beads/mg of peptides) that have been resolubilized in 1% FA–40% ACN.

Once again, the supernatant was incubated on new washed beads for 1 h. The surnageant were freeze-dried. The IMAC beads are deposited on a 2-layer C18 stage-tip (3 M Empore Dics, 2215-C18) previously washed with methanol and a solution containing 50% ACN–0.1% TFA. They are then washed three times with 1% FA–40% ACN, two times with 1% FA. The phosphorylated peptides were eluted from the beads with 500 mM KHPO_4_ pH 7, then washed twice with 1% FA and then eluted from the C18 phase with 40% ACN–1% FA. The two incubations on beads were pooled before being dried in a speed-vac.

The unbound peptides following IMAC purification were solubilized in 2 M lactic acid–50% ACN. They were purified on TiO_2_ beads previously washed with 2 M lactic acid–50% ACN (1 mg of TiO_2_/mg of peptides). The bead/peptide mixture was incubated for 1 h at room temperature. Once again, the supernatant was incubated on new beads for 1 h.

The TiO_2_ beads were deposited on a 2-layer C8 stage-tip previously washed with methanol and a solution containing 60% ACN–1% TFA. They were then washed one time with 60% ACN–1% TFA. The phosphorylated peptides were eluted from the beads, two times, with 40% ACN–15% NH_4_OH 28%. The two incubations on beads were pooled before being dried in a speed-vac.

#### Sample analysis by LC MS/MS

For each purification (immunoaffinity, via IMAC beads, via TiO_2_ beads), phosphopeptides were separated by Reverse-phase C18 using an u3000 RSLC chromatographic system from Dionex on a 2 µm particle size, 100 Angström pore size, 75 µm internal diameter, 25 cm length C18 reverse-phase analytical column with a 150 min binary gradient from 99% solution A (0.1% FA in H_2_O) to 55% solution B (80% ACN, 0.085% FA) before injection into an Orbitrap Fusion Mass Spectrometer MS. MS data acquisition was performed throughout the elution process in a data-dependent scheme (top speed mode in 5 s) with full MS scans acquired with the orbitrap detector, followed by HCD peptide fragmentation and Ion-trap fragment detection of the most abundant ions detected in the MS scan. Mass spectrometer settings for full-scan MS were: 2.0E5 AGC, 60,000 target resolution, 350–1500 *m/z* range, maximum ion injection time (MIIT) of 60 ms. HCD MS/MS fragmentation was permitted for 2–7+ precursor ions reaching more than 5.0E3 minimum intensity. Quadrupole-filtered precursors within 1.6 *m/z* isolation window were fragmented with a Normalized Collision Energy setting at 30. 2.0E4 AGC Target and 100 ms MIIT were the limiting ion accumulation values. The Ion-trap detector was used for its fast and sensitive detection capabilities. A 30 s dynamic exclusion time was set.

#### Data processing protocol

Maxquant software version 1.6.17.0 was used for the data extraction from the raw MS files from the mass spectrometer. For each sample, the data from each purification were pooled by giving them the same experiment name. The databases used were the human sequences from the Uniprot database (March 2020, 42384 entries) and a list of in-house frequent contaminant sequences. The labels quantification was: heavy (arginine-13C6-15N4 (Arg10), lysine-13C6-15N2 (lys8)) and medium (arginine-13C6 (Arg6), lysine-13C6 (Lys6)). The cleavage specificity was trypsin’s with a maximum of two missed cleavages. Carbamidomethylation of cysteines was set as a constant modification, whereas acetylation of the protein N terminus, oxidation of methionines, and phosphorylation of serine, threonine, and tyrosine were set as variable modifications. The false discovery rate was kept below 1% on both peptides and proteins. The “match between runs” (MBR) option was allowed with a match time window of 1 min and an alignment time window of 30 min but the normalization was not allowed. For statistical analysis, data were imported into the Perseus software version 1.6.14.0. Reverse and contaminant proteins were excluded from analysis. Only phosphosites with a localization probability >0.75 and a PEP < 1% were kept. Intensity were log2 transformed. Only phosphosites with at least two pairs of valid values were selected to perform a paired *t* test of Student. Phosphosites with a *P* value < 0.05% were kept.

### Immunoprecipitation

Cells were lysed with 20 mM Tris HCl pH 7.5 100 mM NaCl 5 mM EDTA 0.3% NP40 supplemented with protease and phosphatase inhibitors. Cell extracts were precleared with protein A/G magnetic beads for 1 h at 4 °C, and then incubated with either Rabbit IgG (1/200) or Rabbit HA antibody (1/50) for 1 h at 4 °C. Lysates containing IgG or HA antibody were then incubated overnight with 15 µl of slurry beads at 4 °C. Beads were washed twice with the wash buffer (20 mM Tris HCl pH 7.5 100 mM NaCl 5 mM EDTA 0.1% NP40 supplemented with protease and phosphatase inhibitor) followed by three washes with PBS. Beads were eluted with 1× Laemmli. IP efficacy was validated through western blot.

### Proteomic analysis protocols

SDS: Sodium Dodecylsulfate; TCEP: Tris (2-carboxyethyl) phosphine hydrochloride; CAA: chloroacetamide; TFA: Trifluoroacetic Acid; ACN: acetonitrile; TEAB: Triethylammonium bicarbonate; TFE: Trifluoroethanol; HCD: High Collision Dissociation, AGC: Automatic Gain Control.

#### Sample preparation

The IP samples were solubilized in lysis buffer (4% SDS, 400 mM Tris-HCl, pH 8.0, 20 mM TCEP, 100 mM CAA) and boiled 5 min at 95 °C. Bottom-up experiments’ tryptic peptides were obtained by S-Trap Micro Spin Column according to the manufacturer’s protocol. Samples were cleaned on Detergent Removal Spin Columns before being dried in a speed-vac. Peptides were solubilized in 10 μL of 0.1% TFA containing 10% ACN.

#### Sample analysis by LC MS/MS

nLC-MS/MS analyses were performed on a Dionex U3000 RSLC nano-LC- system coupled to a TIMS-TOF Pro mass spectrometer.

One μL was loaded, concentrated, and washed for 3 min on a C_18_ reverse-phase column (5μm particle size, 100 Å pore size, 300 μm inner diameter, 0.5 cm length). Peptides were separated on an Aurora C18 reverse-phase resin (1.6 μm particle size, 100 Å pore size, 75 μm inner diameter, 25 cm length mounted to the Captive nanoSpray Ionization module) with a 1 h run time with a gradient ranging from 98% of solvent A containing 0.1% FA in milliQ-grade H_2_O to 35% of solvent B containing 80% ACN, 0.085% FA in mQ H_2_O.

The mass spectrometer acquired data throughout the elution process and operated in DIA PASEF mode with a 1.38 s/cycle, with Timed Ion Mobility Spectrometry (TIMS) enabled and a data-independent scheme with full MS scans in Parallel Accumulation and Serial Fragmentation (PASEF). Ion accumulation and ramp time in the dual TIMS analyzer were set to 100 ms each, and the ion mobility range was set from 1/K0 = 0.63 Vs cm^−2^ to 1.43 Vs cm^−2^. Precursor ions for MS/MS analysis were isolated in positive polarity with PASEF in the 100–1.700 *m/z* range by synchronizing quadrupole switching events with the precursor elution profile from the TIMS device.

#### Data processing protocol

The mass spectrometry data were analyzed using DIA-NN version 1.9. The database used for in silico generation of spectral library was a concatenation of Human sequences from the Swissprot database (release 2024-06) and a list of contaminant sequences from Maxquant and from the cRAP (common Repository of Adventitious Proteins). M-Terminus exclusion and carbamidomethylation of cysteins was set as a permanent modification, and one trypsin misscleavage was allowed. The precursor false discovery rate (FDR) was kept below 1%. The normalization and “match between runs” (MBR) option was allowed. A Welch *T* test one-tailed (greater in the condition DOX-HA) Paired were done on proteins showing at least two pairs of valid values using log2(LFQ intensity).

### Western blot and SUnSET assays

Cells were lysed in 50 mM Tris-HCl pH 7.5, 150 mM NaCl, 2 mM EDTA, 1% NP40, 0.1% SDS, and 0.5% sodium deoxycholate supplemented with protease and phosphatase inhibitors. Protein concentration was measured using BCA. Equal amounts of proteins were resolved on 10% denaturing polyacrylamide gels and were transferred to nitrocellulose membranes. Membranes were blocked for 1 h with TBS-0.01%Tween20-5%milk then incubated overnight with primary antibodies against CDC25A (Santa Cruz sc-7389), CDC25B (Santa Cruz sc-326), GAPDH (Santa Cruz sc-32233), Puromycin (Millipore, MABE343), p4EBP (Cell Signaling Technologies 9456 P), 4EBP (Cell Signaling Technologies 9644S), pS6K (Cell Signaling Technologies 9234 P), S6K (Cell Signaling Technologies 2708), Actin (Chemi-Con MAB1501), peIF2a (Cell Signaling Technologies 3398S), eIF2a (Cell signaling Technologies 3398S), EEA1 (santa cruz sc-53939), CDK2 (Santa Cruz sc-163), RPS6 (Santa Cruz sc-74459), P-RPS6 (Cell signaling 2215S), eEF1D (proteintech PR-60085), eEF2 (Bethyl BE-A301-688A-T), PKM2 (Cell Signaling 1398), P-PKM2 (Invitrogen PA5-37684), Tubulin (Santa Cruz sc-5286) in TBS-0.01%Tween20-1%BSA. Membranes were incubated with the secondary antibody, either HRP-conjugated anti-rabbit (1:10,000, Ozyme 7074S) or HRP-conjugated anti-mouse (1:10,000, Ozyme 7076S) for 1 h at RT in TBS-0.01%Tween20-5% milk. Membranes were revealed using the ECL system according to the manufacturer’s directions, and images quantified using FIJI software. For SUnSET assays, cells were treated or not with 100 µg/mL cycloheximide (CHX, Sigma, C7698) (negative control) for 5 min, and then labeled with 10 µg/mL (western blot analysis) or 1 µg/mL (flow cytometry analysis) puromycin for 10 min at 37 °C prior to western blot or flow cytometry analysis, respectively.

### Flow cytometry

Cells were trypsinized and washed in PBS before fixation and permeabilization with PBS–4% PFA–0.01% saponine–5% BSA and washed twice with PBS–0.01% saponine-1%BSA. Cells were incubated with anti-puromycin antibody coupled with Alexa 647 (508, dilution 1/50) in wash buffer for 1 h 15 min at RT. Cells were washed three times and resuspended in PBS–1% BSA at a concentration of 1.10e6 cells/mL. When necessary, cells were resuspended in PBS–1% BSA containing 1 µg/mL DAPI. A minimum of 10,000 cells were analyzed per condition. Data analysis was carried out using FlowJo v10.

### Immunofluorescence

Cells were grown on Ibidi (Ibidi µ-slides 8 wells) coated with poly-D-lysine solution. Cells were fixed with 4% paraformaldehyde in PBS for 10 min at RT, permeabilized in PBS–0.5% tritonX100 for 10 min, and blocked in PBS–3% BSA for 1 h at RT. Cells were incubated in HA antibody (Cell Signaling Technologies 3724, 1/50) overnight at 4 °C and incubated with goat anti-rabbit IgG secondary antibody coupled to Alexa Fluor 488 (1:500) in PBS–3% BSA for 1 h at RT. Cells were washed three times and then incubated in mounting media containing DAPI until analysis. Cells were analyzed using a high-resolution confocal microscope (Zeiss, LSM880).

### Proximity ligation assay

Cells were grown on 1 cm^2^ coverslip coated with poly-D-lysine solution. The day after, cells were treated with doxycycline for 24 h. Cells were washed with PBS, fixed with PBS–4%PFA for 20 min, blocked and permeabilized in PBS–5% BSA–0.01% saponine for 1 h at RT. Cells were incubated either with rabbit HA antibody (SIGMA A2095 1/50), mouse eEF1A1 antibody (Proteintech 67495; 1/200), mouse eEF1D antibody (Proteintech 60085; 1/400), rabbit eEF2 antibody (bethyl BE-A301-688A-T; 1/500), mouse HA antibody (SIGMA H9658; 1/600), or with combinations of two antibodies. For the PLA, the Duolink kit was used, and the rest of the assay was performed as per the manufacturer’s protocol. Cells were visualized using a high-resolution confocal microscope (Zeiss, LSM880). The number of dots was quantified using a FIJI macro.

### Polysome profiling

For a detailed protocol, see (Cammas et al, 2022). Briefly, HEK293 cells were treated with 100 µg/mL cycloheximide (CHX) for 5 min at 37 °C, washed with ice-cold PBS- 100 µg/mL CHX, and scraped on ice. After centrifugation for 5 min at 1200 rpm, the cell pellet was gently resuspended in 450 μL of hypotonic lysis buffer (5 mM Tris pH 7.5, 2.5 mM MgCl_2_, 1.5 mM KCl, 100 µg/mL CHX, 2 mM DTT, 200 U/mL RNaseOUT), vortexed for 5 s, and incubated on ice for 5 min. After the addition of 0.5% Triton X-100 and 0.5% sodium deoxycholate, the lysate was vortexed for 5 s and incubated on ice for 5 min. The lysate was then centrifuged at 16,000× *g* for 7 min at 4 °C, and a volume of the cytosolic fraction corresponding to 20 OD_260_ nm was layered on a 11.3 mL continuous sucrose gradient (5–50% sucrose in 20 mM HEPES pH 7.6, 0.1 M KCl, 5 mM MgCl_2_, 10 µg/mL CHX, 10 U/mL RNaseOUT). After 2 h of ultracentrifugation at 222,228× *g* in a SW41-Ti rotor at 4 °C, fractions were collected with an ISCO density gradient fractionation system (Foxy Jr fraction collector coupled to UA-6UV detector, Lincoln, NE). The settings were as follows: fraction time, 35 s/fraction; sensitivity of the OD254 recorder, 1. Absorbance at 254 nm was measured continuously as a function of gradient depth; 16 fractions of approximately 0.8 mL were collected. Protein from individual fractions was extracted by using isopropanol precipitation and analyzed by western blot.

### Statistical analysis

Statistical analysis of the difference between two sets of data was assessed using two-sided paired *t* test or one-way ANOVA (GraphPad Prism). *P* values of less than 0.05 were considered to be significant (*P < 0.05; **P < 0.01; ***P < 0.001, ****P < 0.001).

## Supplementary information

Peer Review File

Data set EV1

Dataset EV2

Source data Fig. 1

Source data Fig. 2

Source data Fig. 3

Source data Fig. 4

Source data Fig. 5

Source data Fig. 6

EV Figures Source Data

Expanded View Figures

## Data Availability

The datasets produced in this study are available in the following databases: mass spectrometry proteomics for the interactome: ProteomeXchange Consortium PXD066262 (PRIDE partner repository); mass spectrometry proteomics for the phosphoproteome: ProteomeXchange Consortium PXD066447 (PRIDE partner repository). The source data of this paper are collected in the following database record: biostudies:S-SCDT-10_1038-S44319-026-00852-y.

## References

[CR1] Aramayo R, Polymenis M (2017) Ribosome profiling the cell cycle: lessons and challenges. Curr Genet 63:959–964 28451847 10.1007/s00294-017-0698-3 PMC5790165

[CR2] Bertoli S, Boutzen H, David L, Larrue C, Vergez F, Fernandez-Vidal A, Yuan L, Hospital MA, Tamburini J, Demur C et al (2015) CDC25A governs proliferation and differentiation of FLT3-ITD acute myeloid leukemia. Oncotarget 6:38061–38078 26515730 10.18632/oncotarget.5706 PMC4741984

[CR3] Bhowmick NA, Ghiassi M, Aakre M, Brown K, Singh V, Moses HL (2003) TGF-beta-induced RhoA and p160ROCK activation is involved in the inhibition of Cdc25A with resultant cell-cycle arrest. Proc Natl Acad Sci USA 100:15548–15553 14657354 10.1073/pnas.2536483100 PMC307605

[CR4] Boutros R, Lobjois V, Ducommun B (2007) CDC25 phosphatases in cancer cells: key players? Good targets? Nat Rev Cancer 7:495–507 17568790 10.1038/nrc2169

[CR5] Cammas A, Herviou P, Dumas L, Millevoi S (2022) Analysis of mRNA translation by polysome profiling. Methods Mol Biol 2404:69–81 34694604 10.1007/978-1-0716-1851-6_4

[CR6] Feng X, Wu Z, Wu Y, Hankey W, Prior TW, Li L, Ganju RK, Shen R, Zou X (2011) Cdc25A regulates matrix metalloprotease 1 through Foxo1 and mediates metastasis of breast cancer cells. Mol Cell Biol 31:3457–3471 21670150 10.1128/MCB.05523-11 PMC3147788

[CR7] Ferguson AM, White LS, Donovan PJ, Piwnica-Worms H (2005) Normal cell cycle and checkpoint responses in mice and cells lacking Cdc25B and Cdc25C protein phosphatases. Mol Cell Biol 25:2853–2860 15767688 10.1128/MCB.25.7.2853-2860.2005 PMC1061644

[CR8] Fernandez-Vidal A, Ysebaert L, Didier C, Betous R, De Toni F, Prade-Houdellier N, Demur C, Contour-Galcéra M-O, Prévost GP, Ducommun B et al (2006) Cell adhesion regulates CDC25A expression and proliferation in acute myeloid leukemia. Cancer Res 66:7128–7135 16854822 10.1158/0008-5472.CAN-05-2552

[CR9] Herviou P, Le Bras M, Dumas L, Hieblot C, Gilhodes J, Cioci G, Hugnot J-P, Ameadan A, Guillonneau F, Dassi E et al (2020) hnRNP H/F drive RNA G-quadruplex-mediated translation linked to genomic instability and therapy resistance in glioblastoma. Nat Commun 11:2661 32461552 10.1038/s41467-020-16168-x PMC7253433

[CR10] Hornbeck PV, Zhang B, Murray B, Kornhauser JM, Latham V, Skrzypek E (2015) PhosphoSitePlus, 2014: mutations, PTMs and recalibrations. Nucleic Acids Res 43:D512–D520 25514926 10.1093/nar/gku1267 PMC4383998

[CR11] Jackson RJ, Hellen CU, Pestova TV (2010) The mechanism of eukaryotic translation initiation and principles of its regulation. Nat Rev Mol Cell Biol 11:113 20094052 10.1038/nrm2838 PMC4461372

[CR12] Kejiou NS, Ilan L, Aigner S, Luo E, Tonn T, Ozadam H, Lee M, Cole GB, Rabano I, Rajakulendran N et al (2023) Pyruvate kinase M (PKM) binds ribosomes in a poly-ADP ribosylation dependent manner to induce translational stalling. Nucleic Acids Res 51:6461–6478 37224531 10.1093/nar/gkad440 PMC10325899

[CR13] Kim Y, Lee JH, Park J-E, Cho J, Yi H, Kim VN (2014) PKR is activated by cellular dsRNAs during mitosis and acts as a mitotic regulator. Genes Dev 28:1310–1322 24939934 10.1101/gad.242644.114 PMC4066401

[CR14] Kittipatarin C, Li WQ, Bulavin DV, Durum SK, Khaled AR (2006) Cell cycling through Cdc25A: transducer of cytokine proliferative signals. Cell Cycle 5:907–912 16628013 10.4161/cc.5.9.2693

[CR15] Kiyokawa H, Ray D (2008) In vivo roles of CDC25 phosphatases: biological insight into the anti-cancer therapeutic targets. Anticancer Agents Med Chem 8:832–836 19075565 10.2174/187152008786847693 PMC2753225

[CR16] Kuleshov MV, Jones MR, Rouillard AD, Fernandez NF, Duan Q, Wang Z, Koplev S, Jenkins SL, Jagodnik KM, Lachmann A et al (2016) Enrichr: a comprehensive gene set enrichment analysis web server 2016 update. Nucleic Acids Res 44:W90–W97 27141961 10.1093/nar/gkw377 PMC4987924

[CR17] Liang J, Cao R, Zhang Y, Xia Y, Zheng Y, Li X, Wang L, Yang W, Lu Z (2016) PKM2 dephosphorylation by Cdc25A promotes the Warburg effect and tumorigenesis. Nat Commun 7:12431 27485204 10.1038/ncomms12431 PMC4976202

[CR18] Lin KW, Yakymovych I, Jia M, Yakymovych M, Souchelnytskyi S (2010) Phosphorylation of eEF1A1 at Ser300 by TβR-I results in inhibition of mRNA translation. Curr Biol 20:1615–1625 20832312 10.1016/j.cub.2010.08.017

[CR19] Lin Y, Li F, Huang L, Polte C, Duan H, Fang J, Sun L, Xing X, Tian G, Cheng Y et al (2020) eIF3 associates with 80S ribosomes to promote translation elongation, mitochondrial homeostasis, and muscle health. Mol Cell 79:575–587.e7 32589965 10.1016/j.molcel.2020.06.003

[CR20] Lin YM, Chung CL, Cheng YS (2009) Posttranscriptional regulation of CDC25A by BOLL is a conserved fertility mechanism essential for human spermatogenesis. J Clin Endocrinol Metab 94:2650–2657 19417033 10.1210/jc.2009-0108

[CR21] Malumbres M, Barbacid M (2005) Mammalian cyclin-dependent kinases. Trends Biochem Sci 30:630–641 16236519 10.1016/j.tibs.2005.09.005

[CR22] Mazars A, Fernandez-Vidal A, Mondesert O, Lorenzo C, Prévost G, Ducommun B, Payrastre B, Racaud-Sultan C, Manenti S (2009) A caspase-dependent cleavage of CDC25A generates an active fragment activating cyclin-dependent kinase 2 during apoptosis. Cell Death Differ 16:208–218 18927589 10.1038/cdd.2008.142

[CR23] Mazzolini L, Broban A, Froment C, Burlet-Schiltz O, Besson A, Manenti S, Dozier C (2016) Phosphorylation of CDC25A on SER283 in late S/G2 by CDK/cyclin complexes accelerates mitotic entry. Cell Cycle 15:2742–2752 27580187 10.1080/15384101.2016.1220455 PMC5053558

[CR24] Ray D, Terao Y, Fuhrken PG, Ma Z-Q, DeMayo FJ, Christov K, Heerema NA, Franks R, Tsai SY, Papoutsakis ET et al (2007a) Deregulated CDC25A expression promotes mammary tumorigenesis with genomic instability. Cancer Res 67:984–991 17283130 10.1158/0008-5472.CAN-06-3927

[CR25] Ray D, Terao Y, Nimbalkar D, Hirai H, Osmundson EC, Zou X, Franks R, Christov K, Kiyokawa H (2007b) Hemizygous disruption of Cdc25A inhibits cellular transformation and mammary tumorigenesis in mice. Cancer Res 67:6605–6611 17638870 10.1158/0008-5472.CAN-06-4815

[CR26] Rowland RJ, Korolchuk S, Salamina M, Tatum NJ, Ault JR, Hart S, Turkenburg JP, Blaza JN, Noble MEM, Endicott JA (2024) Cryo-EM structure of the CDK2-cyclin A-CDC25A complex. Nat Commun 15:6807 39122719 10.1038/s41467-024-51135-w PMC11316097

[CR27] Shen T, Huang S (2012) The role of Cdc25A in the regulation of cell proliferation and apoptosis. Anticancer Agents Med Chem 12:631–639 22263797 10.2174/187152012800617678 PMC3544488

[CR28] Sohn J, Rudolph J (2006) The energetic network of hotspot residues between Cdc25B phosphatase and its protein substrate. J Mol Biol 362:1060–1071 16950393 10.1016/j.jmb.2006.07.090 PMC1769329

[CR29] Stonyte V, Boye E, Grallert B (2018) Regulation of global translation during the cell cycle. J Cell Sci 131:jcs220327 30072440 10.1242/jcs.220327

[CR30] Stumpf CR, Moreno MV, Olshen AB, Taylor BS, Ruggero D (2013) The translational landscape of the mammalian cell cycle. Mol Cell 52:574–582 24120665 10.1016/j.molcel.2013.09.018 PMC3959127

[CR31] Sueur G, Boutet A, Gotanègre M, Mansat-De Mas V, Besson A, Manenti S, Bertoli S (2020) STAT5-dependent regulation of CDC25A by miR-16 controls proliferation and differentiation in FLT3-ITD acute myeloid leukemia. Sci Rep 10:1906 32024878 10.1038/s41598-020-58651-x PMC7002454

[CR33] Sun R, Cheng E, Velásquez C, Chang Y, Moore PS (2019) Mitosis-related phosphorylation of the eukaryotic translation suppressor 4E-BP1 and its interaction with eukaryotic translation initiation factor 4E (eIF4E). J Biol Chem 294:11840–11852 31201269 10.1074/jbc.RA119.008512 PMC6682726

[CR34] Szklarczyk D, Gable AL, Nastou KC, Lyon D, Kirsch R, Pyysalo S, Doncheva NT, Legeay M, Fang T, Bork P et al (2021) The STRING database in 2021: customizable protein-protein networks, and functional characterization of user-uploaded gene/measurement sets. Nucleic Acids Res 49:D605–D612 33237311 10.1093/nar/gkaa1074 PMC7779004

[CR35] Tarn W-Y, Lai M-C (2011) Translational control of cyclins. Cell Div 6:5 21314915 10.1186/1747-1028-6-5 PMC3048474

[CR36] Wang J, Huo K, Ma L, Tang L, Li D, Huang X, Yuan Y, Li C, Wang W, Guan W et al (2011) Toward an understanding of the protein interaction network of the human liver. Mol Syst Biol 7:536 21988832 10.1038/msb.2011.67 PMC3261708

[CR37] Xu H, Yu S, Peng K, Gao L, Chen S, Shen Z, Han Z, Chen M, Lin J, Chen S et al (2021) The role of EEF1D in disease pathogenesis: a narrative review. Ann Transl Med 9:1600 34790806 10.21037/atm-21-5025 PMC8576685

[CR38] Zou X, Tsutsui T, Ray D, Blomquist JF, Ichijo H, Ucker DS, Kiyokawa H (2001) The cell cycle-regulatory CDC25A phosphatase inhibits apoptosis signal-regulating kinase 1. Mol Cell Biol 21:4818–4828 11416155 10.1128/MCB.21.14.4818-4828.2001 PMC87174

