# Supplementary material for: Novel CDK-independent function of CDC25 phosphatases in mRNA translation
Source: EMBO Rep. 2026 Jul 14;27(16):4879–902. doi: 10.1038/s44319-026-00852-y (PMC13503811; doi:10.1038/s44319-026-00852-y)
Supplement: Supplementary file 1 [file 44319_2026_852_MOESM11_ESM.pdf]

Expanded View Figures

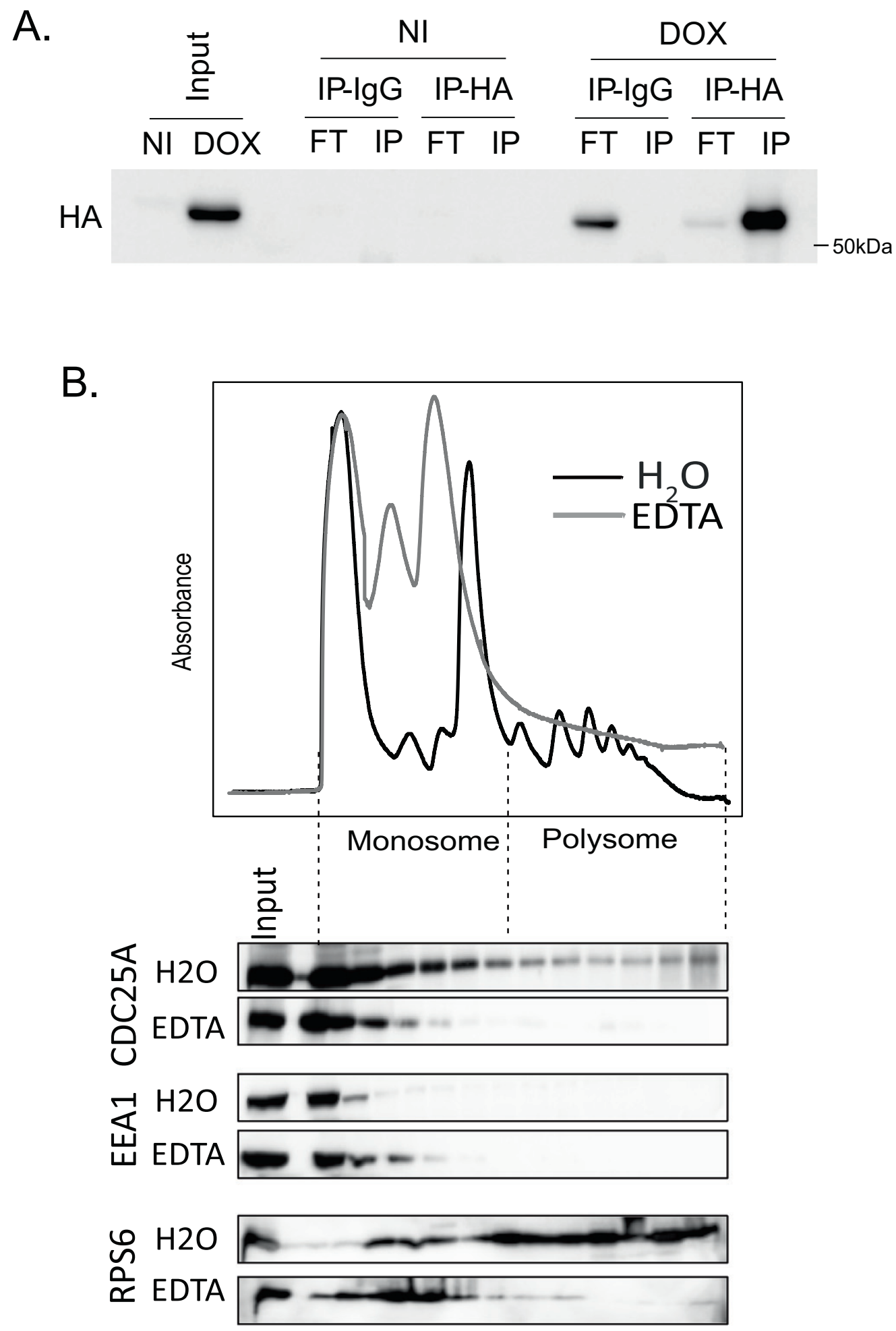

Figure EV1. CDC25A immunoprecipitation for its interactome analysis.

(**A**) HEK293 cells were induced for HA-CDC25A expression for 24 h with doxycycline 1μg/ml (DOX) or left non-induced (NI). Immunoprecipitation of HA-CDC25A from cell extracts was performed with an anti-HA antibody. Aliquots of the inputs, flow through (FT), and immunoprecipitates (IP) were analyzed by western blot with the anti-HA antibody to check the efficiency of the immunoprecipitation. (**B**) Polysome sedimentation profile of HEK293 cells induced for HA-CDC25A expression for 24 h (DOX; upper panel), and western blot analysis of HA-CDC25A in the monosomal and polysomal fractions (lower panel), in the absence (H2O) or the presence of EDTA. Western blots of EEA1 and RPS6 were used as negative and positive controls, respectively. Shown is a representative result from $n = 2$ independent experiments. Source data are available online for this figure.

 © The Author(s)

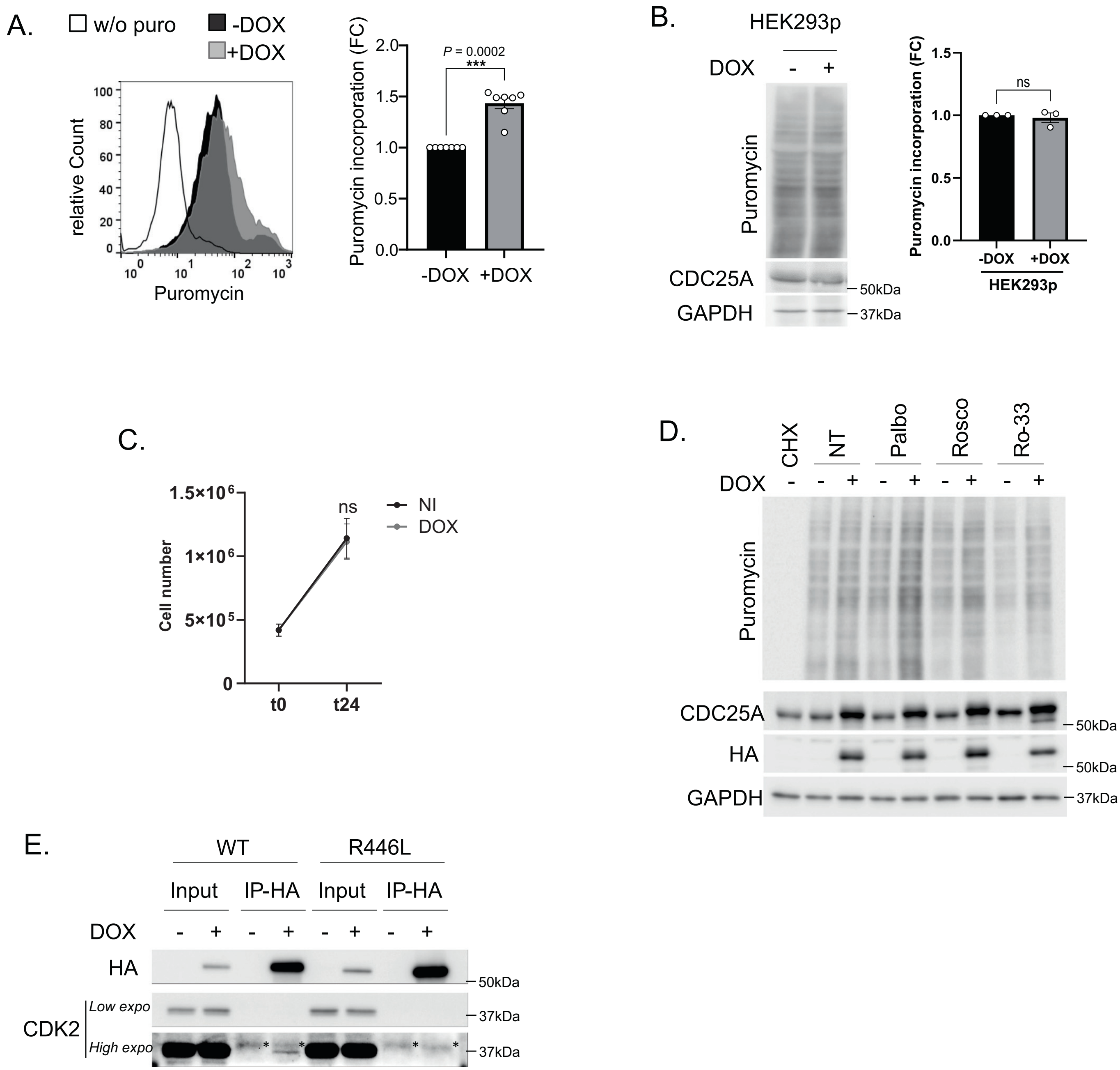

Figure EV2. CDC25A overexpression promotes global translation independently of the cell cycle.

(**A**) Flow cytometry SUnSET analysis (left panel) and quantification (right panel) of global translation in response to CDC25A induction (DOX) in HEK293 cells. Data are presented as mean values ± SEM of $n = 7$ independent experiments, ***$P < 0.0001$, ns: non-significant (two-sided paired t test). (**B**) Western blot SUnSET analysis of puromycin incorporation in response to doxycycline treatment in parental HEK293 (HEK293p) cell line. Data are presented as mean values ± SEM of $n = 3$ independent experiments, ns: non-significant (two-sided paired t test). (**C**) Impact of CDC25A induction (DOX) for 24 h on total HEK293 cell number. NI not induced. Data are presented as mean values ± SEM of $n = 3$ independent experiments, ns: non-significant (two-sided paired t test). (**D**) Western blot SUnSET analysis of puromycin incorporation in HEK293 cells induced for CDC25A expression (DOX) for 24 h, and further treated for four additional hours with the CDK inhibitors Palbocyclib (Palbo; 1 µM), Roscovitine (Rosco;1 µM), and RO-3306 (RO-33; 10 µM). (**E**) Immunoprecipitation using anti-HA antibody of WT or R245L HA-CDC25A from cell extracts of HEK293 cells induced for HA-CDC25AWT or HA-CDC25A-R245L mutant (for 24 h) with doxycycline (DOX) or left non-induced (NI). Inputs, flow through (FT), and immunoprecipitates (IP) were analyzed by western blot with the anti-HA and CDK2 antibodies. *Non-specific (ns). Source data are available online for this figure.

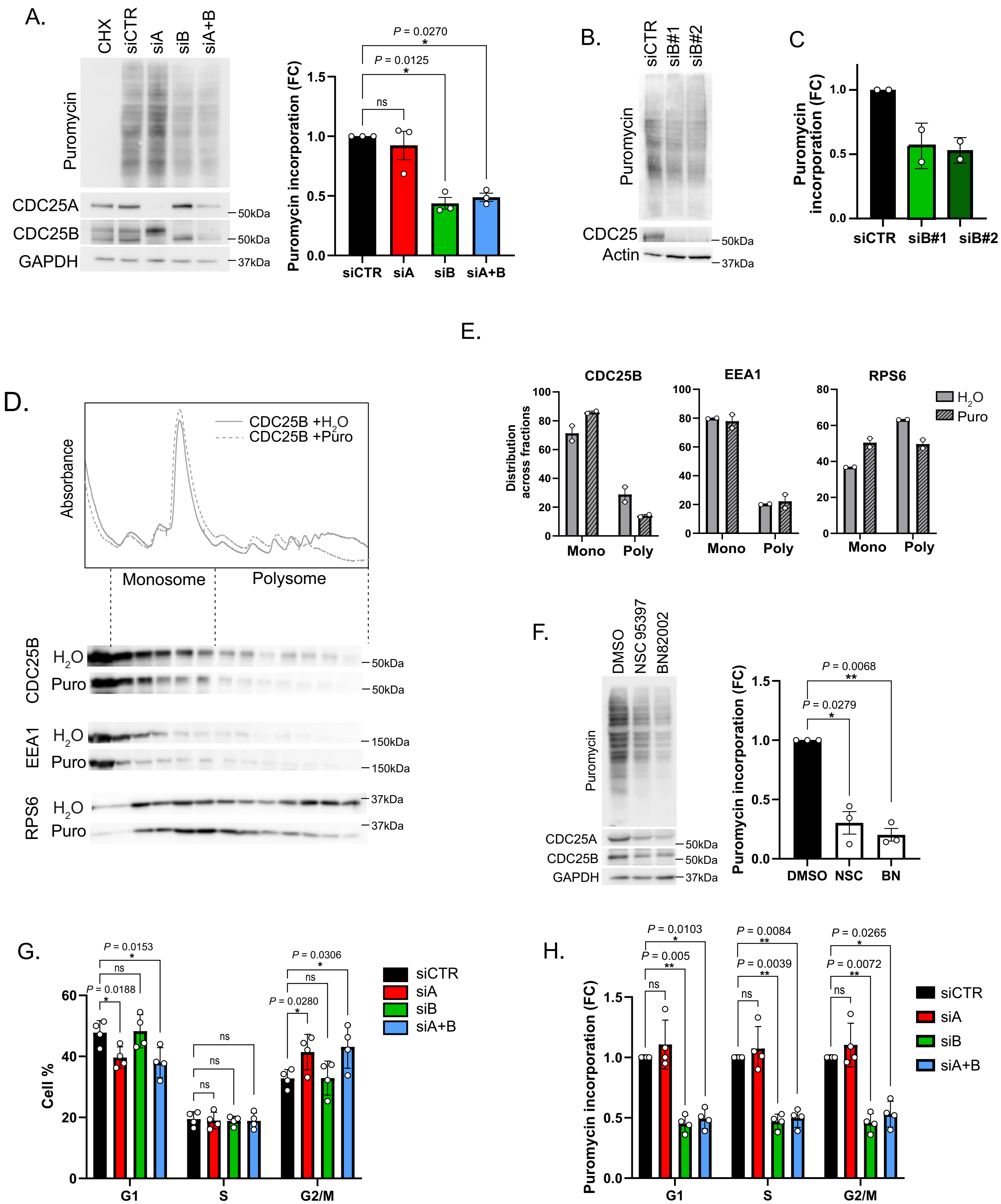
A.
CHX
siCTR
siA
siB
siA+B
Puromycin
CDC25A
CDC25B
GAPDH
50kDa
50kDa
37kDa
Puromycin incorporation (FC)
P = 0.0270
P = 0.0125
ns
siCTR siA siB siA+B
B.
siCTR siB#1 siB#2
Puromycin
CDC25
Actin
50kDa
37kDa
C
Puromycin incorporation (FC)
siCTR siB#1 siB#2
D.
Absorbance
CDC25B +H2O
CDC25B +Puro
Monosome
Polysome
CDC25B
EEA1
RPS6
H2O
Puro
150kDa
E.
CDC25B
EEA1
RPS6
Distribution across fractions
Mono
Poly
H2O
Puro
F.
DMSO
NSC 95397
BN82002
Puromycin
P = 0.0068
P = 0.0279
DMSO NSC BN
G.
Cell %
P = 0.0153
P = 0.0188
P = 0.0306
P = 0.0280
G1 S G2/M
siCTR
siA
siB
siA+B
H.
P = 0.0103
P = 0.005
P = 0.0084
P = 0.0039
P = 0.0265
P = 0.0072

 © The Author(s)

Figure EV3. Compensation between CDC25A and CDC25B.

(**A**) Western blot analysis (left panel) and quantification (right panel) of puromycin incorporation, and of CDC25A and CDC25B protein levels in response to siRNA-mediated depletion of CDC25A (siA) and/or CDC25B (siB) in HEK293 cells. Cycloheximide treatment (CHX) was used as a positive control of translation inhibition. Data are presented as mean values ± SEM of $n = 3$ or independent experiments, *$P < 0.05$, ns: non-significant (two-way ANOVA). (**B**, **C**) Western blot analysis (**B**) and quantification (**C**) of puromycin incorporation, and of CDC25B protein levels in response to siRNA-mediated depletion of CDC25B using two distinct siRNA (siB#1 and siB#2) in HEK293 cells. Data are presented as mean values ± SEM of $n = 2$ independent experiments. (**D**) Polysome sedimentation profile of HEK293 cells transfected with CDC25B for 48 h (upper panel), and western blot analysis of CDC25B in the monosomal and polysomal fractions (lower panel), in the absence (H_2O) or the presence of puromycin (Puro). Western blots of EEA1 and RPS6 were used as negative and positive controls, respectively. Shown is a representative result from $n = 3$ independent experiments. (**E**) Quantification of CDC25B, EEA1, and RPS6 western blots in monosomal (Mono) and polysomal (Poly) fractions in the presence (Puro) or the absence (H_2O) of puromycin. Data are presented as mean values ± SEM of $n = 3$ independent experiments, *$P < 0.05$; **$P < 0.01$, ns: non-significant (repeated measures two-way ANOVA). (**F**) Western blot analysis of puromycin incorporation in HEK293 cells treated for 1 h with CDC25 inhibitors NSC95397 (30 μM) and BN8202 (100 μM). Data are presented as mean values ± SEM of $n = 3$ independent experiments, *$P < 0.05$, **$P < 0.01$ (two-way ANOVA). (**G**) Flow cytometry cell cycle analysis (DAPI labeling) of HEK293 cells in response to CDC25A (siA), CDC25B (siB), or CDC25A and CDC25B (siA+B) depletion. Data are presented as mean values ± SEM of $n = 4$ independent experiments, *$P < 0.05$, ns: non-significant (two-way ANOVA). (**H**) Flow cytometry analysis of puromycin incorporation in response to CDC25A (siA) and/or CDC25B (siB, siA+B) depletion during G1, S, and G2/M phases of the cell cycle. Data are presented as mean values ± SEM of $n = 4$ independent experiments, *$P < 0.05$, **$P < 0.01$, ns: non-significant (two-way ANOVA). Source data are available online for this figure.

© The Author(s)

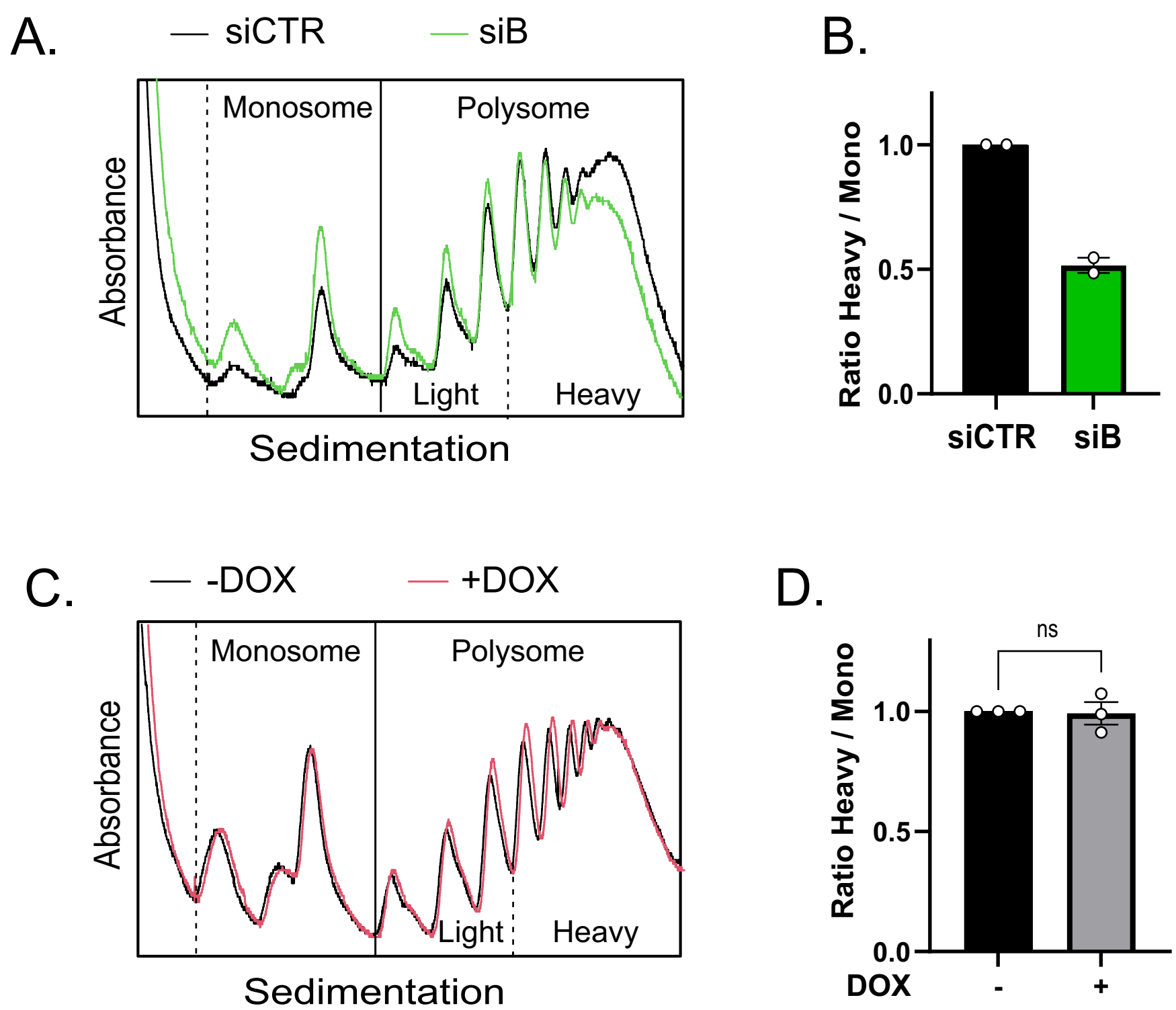

Figure EV4. CDC25B knockdown affects polysome profiling.

(**A–D**) Polysome sedimentation profile of HEK293 after CDC25B depletion (siB) (**A**, **B**), and of non-induced HEK293 cells (-DOX) or induced for HA-CDC25A expression (+DOX) (**C**, **D**). Shown is a representative result from $n = 2$ or 3 independent experiments. (**B**, **D**) Quantification of the ratio between the area under the Polysome curve and the Monosome curve from the polysome profile in (**A**, **C**). Data are presented as mean values ± SEM of $n = 2$ or 3 independent experiments, ns: non-significant (two-sided paired *t* test). Source data are available online for this figure.

 © The Author(s)

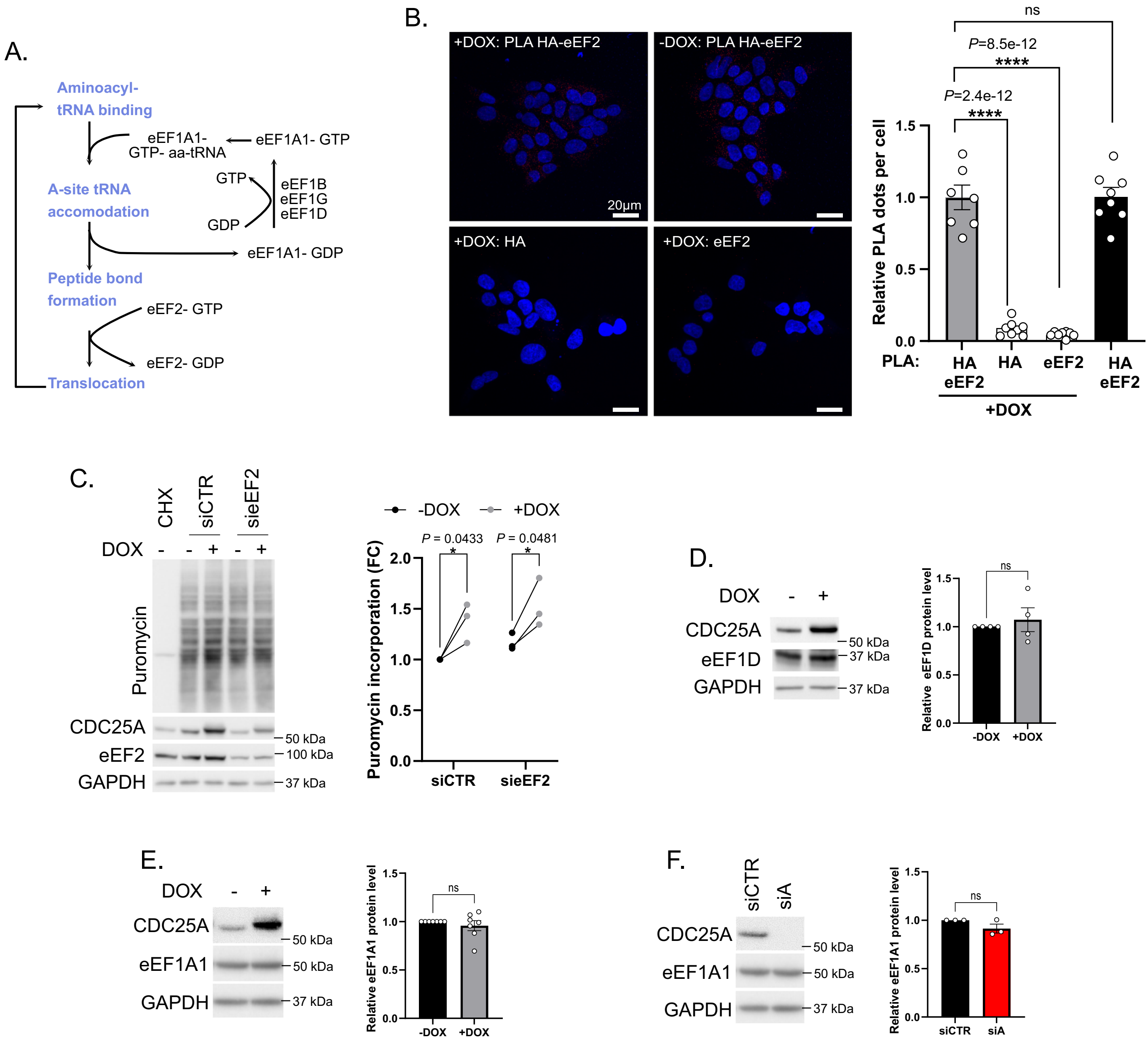

Figure EV5. The variation of CDC25A expression affects translation independently of eEF2.

(**A**) Scheme of the translation elongation cycle. eEF1A1 delivers the aa-tRNA to the A-site of the ribosome. After peptide bond formation, the ribosome is translocated with the help of eEF2. The GTP exchange for eEF1A1 is a complex composed of eEF1B, eEF1G, and eEF1D. (**B**) Immunofluorescence analysis (left panel) and quantification (right panel) of proximity ligation assay experiments performed in HEK293 cells induced for HA-CDC25A expression (DOX) with HA and eEF2 antibody. Negative controls were performed with either eEF2 or HA antibodies alone, or in non-induced cells that do not express HA-CDC25A. Data are presented as mean values ± SEM of $n = 2$ independent experiments (4 images were acquired per experiment; each dot is an image), $^{****}P < 1.e\text{-}11$, ns: non-significant (two-way ANOVA). (**C**) Western blot analysis (left panel) and quantification (right panel) of puromycin incorporation in response to CDC25A induction (DOX) and/or translation elongation factors eEF2 depletion (sieEF2). Data are presented as mean values ± SEM of $n = 3$ independent experiments, $^{*}P < 0.05$ (two-way ANOVA). (**D–F**) Western blot analysis and quantification of eEF1D (**D**) or eEF1A1 (**E**, **F**) protein level in response to CDC25A induction (DOX) or depletion (siA). Data are presented as mean values ± SEM of $n = 3$–7 independent experiments, ns: non-significant (two-sided paired t test). Source data are available online for this figure.

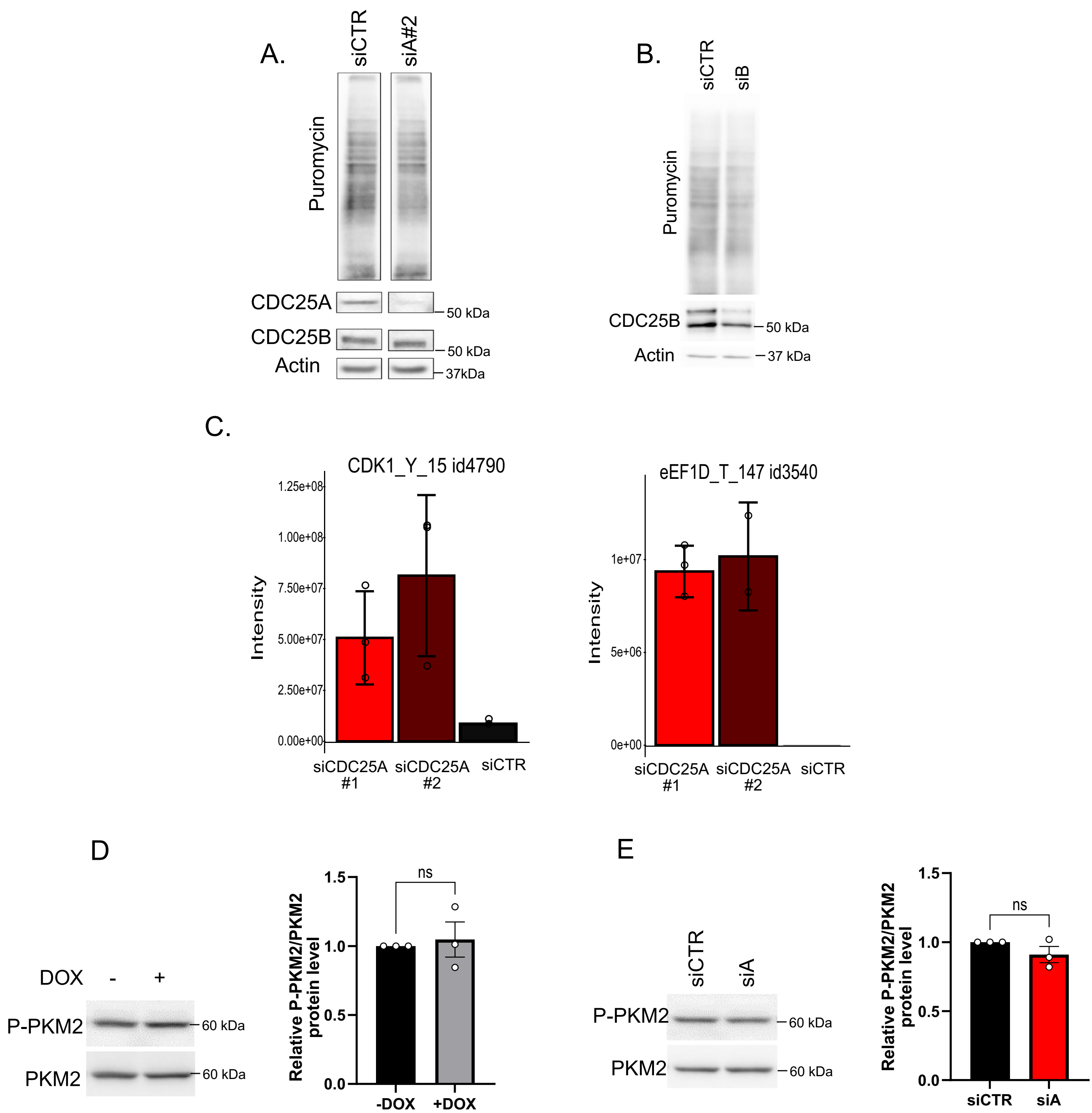

Figure EV6. CDC25A controls CDK1, eEF1D phosphorylation but does not regulate PKM2 phosphorylation.

(**A**) Western blot analysis of puromycin incorporation and of CDC25B protein levels in response to siRNA-mediated depletion of CDC25A using a distinct siRNA (siA#2) in OCI-AML3 cells. (**B**) Western blot analysis of puromycin incorporation in response to siRNA-mediated depletion of CDC25B using a distinct siRNA in OCI-AML3 cells. (**C**) LFQ intensity, identified by mass spectrometry following the SILAC quantitative phosphoproteomic analysis (Fig. 5C, $n = 3$), for CDK1 Tyr15 (Y_15) and eEF1D Thr147 (T_147) in MOLM-14 treated with siRNA control (CTR) or siRNAs against CDC25A (siCDC25A #1 or #2). (**D**, **E**) Western blot analysis of PKM2 and PKM2 phosphorylation (P-PKM2) in response to doxycycline-induced CDC25A overexpression (**D**) or siRNA-mediated depletion (**E**) in HEK293 cells. Data are presented as mean values ± SEM of $n = 3$ independent experiments, ns: non-significant (two-sided paired t test). Source data are available online for this figure.

 © The Author(s)
