# Supplementary material for: Novel CDK-independent function of CDC25 phosphatases in mRNA translation
Source: EMBO Rep. 2026 Jul 14;27(16):4879–902. doi: 10.1038/s44319-026-00852-y (PMC13503811; doi:10.1038/s44319-026-00852-y)
Supplement: Supplementary file 2 [file 44319_2026_852_MOESM1_ESM.pdf]

EMBO reports

Novel CDK-independent function of CDC25 phosphatases in mRNA translation

Sauyeun Shin, Marie Cargnello, Lisa Pinchede, Claire Medale Giamarchi, Cléa Villette, Alexandre Gay, Virginie Salnot, Emilie Fleur Gautier, Erik Dassi, Stefania Millevoi, Anne Cammas, and stéphane manenti

Corresponding author(s): Anne Cammas (anne.cammas@inserm.fr) , Stefania Millevoi (stefania.millevoi@inserm.fr), stéphane manenti (stephane.manenti@inserm.fr)

Review Timeline:

Submission Date:	31st Jul 25
Editorial Decision:	17th Sep 25
Revision Received:	10th Mar 26
Editorial Decision:	24th Apr 26
Revision Received:	29th May 26
Accepted:	12th Jun 26

Editor: Esther Schnapp

Transaction Report:

(Note: With the exception of the correction of typographical or spelling errors that could be a source of ambiguity, letters and reports are not edited. Depending on transfer agreements, referee reports obtained elsewhere may or may not be included in this compilation. Referee reports are anonymous unless the Referee chooses to sign their reports.)

Dear Dr. Cammas,

Thank you for the submission of your manuscript to EMBO reports. We have now received the full set of referee reports that is pasted below.

As you will see, the referees acknowledge that the findings are potentially interesting. However, they all point out that the data are not sufficiently strong for the publication of this work. They have several suggestions for how the study could be strengthened and I think all suggestions are good and should be addressed, except may be referee 3's suggestion that a role for any of the CDC25 target sites should be tested in the CDC25-dependent control of protein synthesis. Naturally, if you have such data, it should be included. Especially all points raised by referee 1 should be addressed.

I understand that we are asking for significant revisions, and the acceptance of your study will depend on the outcome of these revisions. If you have any questions or comments I am also happy to discuss the revisions further with you, also in a video chat, if you like.

I would thus like to invite you to revise your manuscript with the understanding that the referee concerns must be fully addressed and their suggestions taken on board. Please address all referee concerns in a complete point-by-point response. Acceptance of the manuscript will depend on a positive outcome of a second round of review. It is EMBO reports policy to allow a single round of major revision only and acceptance or rejection of the manuscript will therefore depend on the completeness of your responses included in the next, final version of the manuscript.

We realize that it is difficult to revise to a specific deadline. In the interest of protecting the conceptual advance provided by the work, we recommend a revision within 3 months (18th Dec 2025). Please discuss the revision progress ahead of this time with the editor if you require more time to complete the revisions.

You can either publish the study as a short report or as a full article. For short reports, the revised manuscript should not exceed 27,000 characters (including spaces but excluding materials & methods and references) and 5 main plus 5 expanded view figures. The results and discussion sections must further be combined, which will help to shorten the manuscript text by eliminating some redundancy that is inevitable when discussing the same experiments twice. For a normal article there are no length limitations, but it should have more than 5 main figures and the results and discussion sections must be separate. In both cases, the entire materials and methods must be included in the main manuscript file.

IMPORTANT NOTE: we perform an initial quality control of all revised manuscripts before re-review. Your manuscript will FAIL this control and the handling will be DELAYED if the following APPLIES:
1) A data availability section providing access to data deposited in public databases is missing. If you have not deposited any data, please add a sentence to the data availability section that explains that.
2) Your manuscript contains statistics and error bars based on n=2. Please use scatter blots in these cases. No statistics should be calculated if n=2.

When submitting your revised manuscript, please carefully review the instructions that follow below. Failure to include requested items will delay the evaluation of your revision.

1) a .docx formatted version of the manuscript text (including legends for main figures, EV figures and tables). Please make sure that the changes are highlighted to be clearly visible.

2) individual production quality figure files as .eps, .tif, .jpg (one file per figure). See https://wol-prod-cdn.literatumonline.com/pb-assets/embo-site/EMBOPress_Figure_Guidelines_061115-1561436025777.pdf for more info on how to prepare your figures.

3) We replaced Supplementary Information with Expanded View (EV) Figures and Tables that are collapsible/expandable online. A maximum of 5 EV Figures can be typeset. EV Figures should be cited as 'Figure EV1, Figure EV2" etc... in the text and their respective legends should be included in the main text after the legends of regular figures.

- For the figures that you do NOT wish to display as Expanded View figures, they should be bundled together with their legends in a single PDF file called *Appendix*, which should start with a short Table of Content. Appendix figures should be referred to in the main text as: "Appendix Figure S1, Appendix Figure S2" etc. See detailed instructions regarding expanded view here: <https://www.embopress.org/page/journal/14693178/authorguide#expandedview>

- Additional Tables/Datasets should be labeled and referred to as Table EV1, Dataset EV1, etc. Legends have to be provided in a separate tab in case of .xls files. Alternatively, the legend can be supplied as a separate text file (README) and zipped together with the Table/Dataset file.

4) a .docx formatted letter INCLUDING the reviewers' reports and your detailed point-by-point responses to their comments. As

part of the EMBO Press transparent editorial process, the point-by-point response is part of the Review Process File (RPF), which will be published alongside your paper.

5) a complete author checklist, which you can download from our author guidelines <https://www.embopress.org/page/journal/14693178/authorguide>. Please insert information in the checklist that is also reflected in the manuscript. The completed author checklist will also be part of the RPF.

6) Please note that all corresponding authors are required to supply an ORCID ID for their name upon submission of a revised manuscript (<https://orcid.org/>). Please find instructions on how to link your ORCID ID to your account in our manuscript tracking system in our Author guidelines <https://www.embopress.org/page/journal/14693178/authorguide#authorshipguidelines>

7) Before submitting your revision, primary datasets produced in this study need to be deposited in an appropriate public database (see https://www.embopress.org/page/journal/14693178/authorguide#datadeposition). Please remember to provide a reviewer password if the datasets are not yet public. The accession numbers and database should be listed in a formal "Data Availability" section placed after Materials & Method (see also https://www.embopress.org/page/journal/14693178/authorguide#datadeposition). Please note that the Data Availability Section is restricted to new primary data that are part of this study. * Note - All links should resolve to a page where the data can be accessed. *
If your study has not produced novel datasets, please mention this fact in the Data Availability Section.

8) At EMBO Press we ask authors to provide source data for the main manuscript figures. You will receive a separate email with instructions for providing source data with your revised manuscript, including how to upload and organize the files.

9) Our journal also encourages inclusion of *data citations in the reference list* to directly cite datasets that were re-used and obtained from public databases. Data citations in the article text are distinct from normal bibliographical citations and should directly link to the database records from which the data can be accessed. In the main text, data citations are formatted as follows: "Data ref: Smith et al, 2001" or "Data ref: NCBI Sequence Read Archive PRJNA342805, 2017". In the Reference list, data citations must be labeled with "[DATASET]". A data reference must provide the database name, accession number/identifiers and a resolvable link to the landing page from which the data can be accessed at the end of the reference. Further instructions are available at https://www.embopress.org/page/journal/14693178/authorguide#referencesformat

10) Regarding data quantification (see Figure Legends: https://www.embopress.org/page/journal/14693178/authorguide#figureformat)

The following points must be specified in each figure legend:

- the name of the statistical test used to generate error bars and P values,

- the number (n) of independent experiments (please specify technical or biological replicates) underlying each data point,

- the nature of the bars and error bars (s.d., s.e.m.),

- If the data are obtained from n Program fragment delivered error ``Can't locate object method "less" via package "than" (perhaps you forgot to load "than"?) at //ejpvfs23/sites23b/embor_www/letters/embor_decision_revise_and_review.txt line 56.' 2, use scatter blots showing the individual data points.

Discussion of statistical methodology can be reported in the materials and methods section, but figure legends should contain a basic description of n, P and the test applied.

- Please also include scale bars in all microscopy images.

11) The journal requires a statement specifying whether or not authors have competing interests (defined as all potential or actual interests that could be perceived to influence the presentation or interpretation of an article). In case of competing interests, this must be specified in your disclosure statement. Further information: https://www.embopress.org/competing-interests

12) All Materials and Methods need to be described in the main text using our 'Structured Methods' format, which is required for all research articles. According to this format, the Methods section includes a separate Reagents and Tools Table file (listing key reagents, experimental models, software and relevant equipment and including their sources and relevant identifiers) and a Methods and Protocols section describing the methods using a step-by-step protocol format. The aim is to facilitate adoption of the methodologies across labs. More information on how to adhere to this format as well as a downloadable template (.docx) for the Reagents and Tools Table can be found in our author guidelines: https://www.embopress.org/page/journal/14693178/authorguide#structuredmethods.

An example of a Method paper with Structured Methods can be found here: https://www.embopress.org/doi/full/10.1038/s44320-024-00037-6#sec-4

We would also welcome the submission of cover suggestions, or motifs to be used by our Graphics Illustrator in designing a cover.

As part of the EMBO publication's Transparent Editorial Process, EMBO reports publishes online a Review Process File (RPF) to accompany accepted manuscripts. This File will be published in conjunction with your paper and will include the referee reports, your point-by-point response and all pertinent correspondence relating to the manuscript.

You are able to opt out of this by letting the editorial office know (emboreports@embo.org). If you do opt out, the Review Process File link will point to the following statement: "No Review Process File is available with this article, as the authors have chosen not to make the review process public in this case."

I look forward to seeing a revised form of your manuscript when it is ready.

Best regards,
Esther

Esther Schnapp, PhD
Senior Editor
EMBO reports

Referee #1:

This manuscript investigates the role of CDC25A, and partly CDC25B, in mRNA translation. CDC25A is a well-characterized phosphatase essential for cell cycle progression. The authors begin with biochemical evidence indicating interaction between CDC25A and ribosomal proteins, then explore CDC25A's role in mRNA translation. The manuscript presents an interesting and novel observation: increased protein synthesis following transient CDC25A overexpression. It then develops through the functional interaction of both CDC25A and CDC25B with the translational machinery, ultimately suggesting that CDC25A regulates translation at the elongation level, while CDC25B acts at initiation.
However, several logical connections require strengthening, and the manuscript would benefit from extensive, focused, additional experimental validation. In its current form, while the work is suggestive, it appears patchy. Several datasets remain preliminary, making it challenging to derive a definitive conclusion beyond the general possibility that both CDC25A and CDC25B regulate mRNA translation.The authors present their data transparently and demonstrate solid experimental capabilities. Nevertheless, two major gaps limit the mechanistic insights:
Figure 4 demonstrates that CDC25A requires eEF1A1, but not eEF1D, for translation stimulation and shows CDC25A-eEF1A interaction. However, Table 2 reports that CDC25A affects eEF1D phosphorylation but not eEF1A1 phosphorylation. While (since) these experiments employ different approaches (doxycycline-induced CDC25A in HEK293 cells versus downregulation in leukemic cells), the conclusions appear contradictory. The use of two different cell lines with distinct experimental protocols raises questions about the generalizability of the findings. To strengthen this work, consistent analyses should be performed across both cell systems to resolve these apparent discrepancies. I understand this is a huge experimental work, but it is flawed to present different cell lines, with different experiments and make conclusions.
The authors propose that CDC25B significantly affects initiation based on western blotting of phosphorylated initiation factors. Two datasets are presented: CDC25B inhibition downregulates S6K/mTOR target phosphorylation and increases eIF2α phosphorylation. Both events correlate with reduced protein synthesis according to established literature (translation is negatively regulated by eIF2α phosphorylation and positively regulated by S6K/mTOR target phosphorylation). However, these effects appear inversely correlated with phosphatase activity, suggesting that CDC25B's effects on mTOR/S6K1 target dephosphorylation are indirect. The increased phospho-eIF2α levels represent an intriguing observation given GADD34's established role with this initiation factor, but this connection remains unexplored. The authors should attempt to clarify how CDC25A or CDC25B regulates protein synthesis rates, is the phosphatase activity required? Is it indirect?
Additional Issues
The authors report that CDC25A interacts with 55 unique proteins, including several ribosomal proteins and translation factors. This finding differs from published mass spectrometry studies, including those in BioGRID, which have not identified associations between CDC25A and ribosomal machinery components. The enrichment in ribosomal factors following doxycycline administration is less specific compared to enrichment of established CDC25A interactors such as FBXW11 (Table 1), which the authors do observe, validating their experimental approach. In short, the proteomics experiment successfully detects classical CDC25A interactors while identifying novel ribosomal apparatus interactions. However, the experimental design may preferentially capture newly synthesized CDC25A (associated with ribosomal machinery during translation) rather than genuine associations between mature CDC25A protein and ribosomes. Example, if CDC25A was N.-tagged, the risk of

association with translating ribosomes is quite high. However: The methods section lacks crucial details and contains errors, including reference to a non-existent "reference 21" in the statement: "HEK293 cell lines were designed for conditional expression of HA-tagged CDC25A as described in 21." We cannot know how the experiment was done. Please correct. Last, the mass spectrometry study should be repeated using C-terminally tagged protein (if the initial experiment used N-terminal tagging) with doxycycline induction followed by a chase period or additional validations are required (see also below).
The CDC25A shift with puromycin treatment is minimal (the rationale for two exposures is unclear). The rPS6 shift is also modest. Improved experiments are needed; treating extracts with EDTA rather than puromycin might be more informative.
If the authors claim that CDC25A and CDC25B have differential effects on initiation and elongation, appropriate polysomal profiles should be presented. While elongation defects may not alter polysomal profiles, initiation defects should cause substantial 80S accumulation.
The translation increase following short CDC25A induction is noteworthy. The authors appropriately use a mutant that cannot interact with cyclin downstream factors, demonstrating that CDC25A's stimulatory activity on translation is independent of cell cycle regulation. Consider additional experiments: Test a catalytically inactive mutant; Map residues necessary for inducing protein synthesis enhancement; Analyze which mRNAs are preferentially translated upon CDC25A expression
Use multiple siRNAs to exclude off-target effects
Correct methods section for references, typographical errors (e.g., "supernatant"), and proper chemical formulas with subscripts
In conclusion, this manuscript presents intriguing observations that require substantial development. In my opinion, a massive revision and focusing are required, starting from performing different experiments on the same cell line, rather than different experiments on different cellular system, define if the phosphatase activity is required for translational increase, actually showing that initiation and elongation are affected, and not using a proxy. Concerning the physiological relevance, I do not have concerns. If CDC25A and CDC25B really affects initiation and elongation, the physiological relevance will automatically follow. Finally, specific mRNA translation should be addressed. Authors should pick up a specific challenge and solve it.

Referee #2:

In this manuscript, Shin et al describe a role for the cell cycle phosphatases CDC25A and CDC25B in translation regulation independent of CDK activity or cell cycle modulation. Although the results are interesting, they are also somewhat contradictory and it is difficult to build a coherent model on how these proteins are involved in translation or whether the effects are at all direct (see points 3, 4 and 7 below).
Specific comments:
1) Fig 1D: Do endogenous CDC25A and CDC25B co-sediment with polysomes in HEX293T cells? This is important given the results of Fig 3. In this figure, please also indicate which fractions correspond to the 'monosome' and 'polysome' sections of the gradient. In addition, is it water or DMSO as indicated in parts E-F? Please, explain what EEA1 is in the main text and why it is a negative control.
2) Fig S2C: Is there any defect in proliferation beyond 24h? Normally one takes measurements through several days during exponential growth until cells have reached a plateau.
3) Fig 3: An important concern is that no differences in translation are observed upon depletion of CDC25A, not even in conditions where the compensatory CDC25B is absent. How is this explained?. This would rather suggest indirect or non-specific functions of CDC25A over-expression.
4) Figs 4C and S4B: It is noteworthy that depletion of the elongation factors used in these figures invariably results in decreased levels of HA-CDC25A, yet there is no effect on HA-CDC25A-mediated translation for most of them. Furthermore, there is no apparent decrease of translation by depletion of any of the elongation factors in the basal state (compare '-' lanes), which is unexpected.
5) Fig 4E: Please, show PLA of HA-CDC25A and another elongation factor as negative control.
6) Fig 5A: Why is there a difference between 16h and 24h if CDC25A is equally well-depleted at both time points? The results suggest indirect effects of CDC25A on translation. Furthermore, the authors propose that in these cell lines there is no compensation by CDC25B. Can the authors perform a Western-blot to ensure that CDC25B levels do not change?
7) Fig 5D and S5: It is intriguing that phosphorylation of eEF1A1 is not affected by CDC25A depletion, while phosphorylation of eEF1D is. This does not fit with the proposed roles of these factors in mediating CDC25A function in translation in HEK293 cells, as per the results of Figure 4. In these cells, the authors propose that eEF1A1 mediates translation regulation by CDC25A, while eEF1D does not. Perhaps these contradictions are rooted in the use of different cell lines, and therefore the authors may want to repeat their translation experiments (polysome profiles, potential role of CDC25B) in the leukemic cell lines.
8) Given the contradictions, the discussion is highly speculative.
Minor:
A scheme of how the factors evaluated in Fig 4 are involved in translation would help the reader.
Fig S1 has only one panel, not necessary to name a panel here.
Molecular weight markers should be shown in all Western blots.
Make sure to correctly show the formula of water.

Referee #3:

In this manuscript, Shin et al describe experiments to identify interacting partners of CDC25A, using mass spectrometry. This experiment revealed a large portion of proteins with roles in protein synthesis. Based on these findings, the rest of the manuscript tests the hypothesis that CDC25A associates with translating ribosomes to promote protein synthesis. Furthermore, the authors propose that this function is unrelated to the well-established role of CDC25A in cell cycle control, and that while CDC25A is involved in translation initiation, CDC25B is involved in elongation. Lastly, the authors propose that CDC25A regulates translation in AML cells. Functional tests involve over-expression and knock-down experiments to modulate the levels of CDC25. Readout assays include immunoblotting of relevant targets and puromycin experiments as a readout of protein synthesis.

Overall, while the paper makes some new and potentially important observations regarding the role of these phosphatases in protein synthesis, some of the claims are not adequately supported by the evidence shown, as detailed below:

- Their claims notwithstanding, the data in Figure 1D show very clearly that the bulk of CDC25A is not associated with translating ribosomes in the polysomal fractions. The pattern is not even remotely similar to that of RPS6, which (for RPS6) shows most of it in polysomes. While this result does not preclude a role for CDC25A in protein synthesis, it makes the connection more tenuous.

- The results in Figure 2 support a role for CDC25A in promoting protein synthesis, independently of CDC25A's role in the cell cycle (because the R446L mutant that does not interact with the CDK had no effect). But this conclusion relies exclusively on over-expression studies. The effect is also relatively modest (~20-30%) in this experiment. Although the authors invoke a compensatory role for CDC25B to explain the lack of any effect in the converse experiment with the siRNA to lower CDC25A (Figure 3), it leaves the issue open.

- The authors identify several proteins with roles in protein synthesis that have altered phosphorylation in CDC25 perturbations. This is fine, and a good starting point to understand and solidify their previous observations. However, they show no evidence that any of these readouts could explain the purported role of CDC25 in translation. Couldn't they functionally test the role of any of these sites in the CDC25-dependent control of protein synthesis? Along the same lines, an important control that is missing here is a test that the PPase role of CDC25 is necessary. How does a catalytically dead version of CDC25A behave in these experiments?

Overall, the authors present some potentially important observations. Still, they do not go far enough to strengthen their conclusions and provide a convincing picture of how their model might emerge. At this stage of the work, as described in the manuscript, the reader is left with more questions than answers.

EMBOR-2025-62426V1 : **Novel CDK-independent function of CDC25 phosphatases in mRNA translation**
Shin et al.

We are grateful to the reviewers for their comments and suggestions that contributed to further expanding and strengthening the study. For clarity's sake, the modifications in the revised manuscript are indicated in blue.

Reviewer 1

We thank the Reviewer 1 for her/his interest and point out the novelty of our findings, in particular the unexpected link between CDC25 phosphatases and mRNA translation. Answering her/his comments strengthened the connections and the robustness of our datasets.

Major points.

Point 1, **Figure 4 demonstrates that CDC25A requires eEF1A1, but not eEF1D, for translation stimulation and shows CDC25A-eEF1A interaction. However, Table 2 reports that CDC25A affects eEF1D phosphorylation but not eEF1A1 phosphorylation. While (since) these experiments employ different approaches (doxycycline-induced CDC25A in HEK293 cells versus downregulation in leukemic cells), the conclusions appear contradictory. The use of two different cell lines with distinct experimental protocols raises questions about the generalizability of the findings. To strengthen this work, consistent analyses should be performed across both cell systems to resolve these apparent discrepancies. I understand this is a huge experimental work, but it is flawed to present different cell lines, with different experiments and make conclusions.**

We fully agree with reviewer's 1 remarks concerning the suggestion to perform this complete set of experiments in both cell lines (HEK 293 and leukemic cells). However, protein overexpression in leukemic cell models is technically challenging as it requires the generation of lentiviral expression systems, which could not be completed within the revision time. To address this discrepancy, and given the well-established interaction between EEF1A1 and EEF1D (Xu et al, Ann Transl Med, 2021) as well as the effect of EEF1A1 depletion on CDC25A-induced translation (Fig 4C,D), we performed additional experiments to further investigate the functional and physical link between EEF1D and CDC25A. By optimizing the silencing conditions, we observed that, similar to EEF1A1, loss of EEF1D abolished CDC25A-induced translational regulation (new Fig 4E,F). We also showed that CDC25A overexpression did not modify the protein level of EEF1D (Fig EV4 J). Consistently, using PLA, we found that CDC25A interacts not only with EEF1A1 but also with EEF1D (Fig 4G,H). We have updated the text to incorporate these results, which are now more consistent with our findings and with published literature on the EEF1D–EEF1A1 complex and function. We thank the reviewer for this

insightful comment, which prompted us to re-examine the role of EEF1D and thereby strengthen our conclusions regarding CDC25A-mediated translational regulation.

Regarding the apparent discrepancy in CDC25A-dependent phosphorylation changes between EEF1A1 and EEF1D, a phosphorylated EEF1A1 peptide was detected under control conditions, but its abundance was approximately 100-fold lower than that of phosphorylated EEF1D (table EV2), which may reflect either lower stoichiometry or technical limitations in peptide detection. Moreover, the increase in EEF1A1 phosphorylation upon CDC25A depletion was observed in one replicate, suggesting a trend similar to that seen for EEF1D but precluding definitive conclusions. Further analyses, such as optimized phosphopeptide enrichment or targeted mass spectrometry, will be required to determine whether CDC25A regulates EEF1A1 phosphorylation, a question that lies beyond the scope of the present study.

Point 2. **The authors propose that CDC25B significantly affects initiation based on western blotting of phosphorylated initiation factors. Two datasets are presented: CDC25B inhibition downregulates S6K/mTOR target phosphorylation and increases eIF2α phosphorylation. Both events correlate with reduced protein synthesis according to established literature (translation is negatively regulated by eIF2α phosphorylation and positively regulated by S6K/mTOR target phosphorylation). However, these effects appear inversely correlated with phosphatase activity, suggesting that CDC25B's effects on mTOR/S6K1 target dephosphorylation are indirect. The increased phospho-eIF2α levels represent an intriguing observation given GADD34's established role with this initiation factor, but this connection remains unexplored.**

We agree with the reviewer's interpretation regarding the indirect effects of CDC25B on the phosphorylation of mTOR targets, and we have modified the Discussion to include this comment. To address the question of a potential CDC25B–GADD34–EIF2α link, we aimed to overexpress CDC25B and then assess whether GADD34 depletion could reverse the effect. As shown in the figure below, as we did not observe any change in EIF2α phosphorylation following CDC25B overexpression we could not perform this experiment, thus preventing us from drawing conclusions on the role of GADD34. We have modified the Discussion to include this comment.

Figure for referee with unpublished data has been removed upon request by the authors.

The authors should attempt to clarify how CDC25A or CDC25B regulates protein synthesis rates, is the phosphatase activity required? Is it indirect?

We are grateful to the Reviewer 1 for giving us the opportunity to clarify whether CDC25A and CDC25B functions are dependent on their catalytic activities. Consistent with the results using pharmacological inhibitors (Fig EV3 F), we found that overexpression of the catalytically dead CDC25A mutant (C431S) did not affect translation as compared to the wild type CDC25A (Fig 2C). Concerning CDC25B, as mentioned earlier (point 2), overexpression of wild-type CDC25B did not affect the phosphorylation of eIF2α. Moreover, generating and functionally validating a catalytically inactive CDC25B mutant in the appropriate expression system would require substantial additional experimental work that could not be completed within the revision timeframe.
Addressing the requirement for its catalytic activity will be an important objective of future studies.

We believe that these new data shed important light on this question, and we hope this will address the concerns raised by reviewers 1 and 2 concerning this specific point

Additional issues

Point 3. **However, the experimental design may preferentially capture newly synthesized CDC25A (associated with ribosomal machinery during translation) rather than genuine associations between mature CDC25A protein and ribosomes. Example, if CDC25A was N.-tagged, the risk of association with translating ribosomes is quite high. Howewer: The methods section lacks crucial details and contains errors, including reference to a non-existent "reference 21" in the statement: "HEK293 cell lines were designed for conditional expression of HA-tagged CDC25A as described in 21." We cannot know how the experiment was done. Please correct. Last, the mass spectrometry study should be repeated using C-terminally tagged protein (if the initial experiment used N-terminal tagging) with doxycycline induction followed by a chase period or additional validations are required (see also below).**

We sincerely apologize for the omission of reference 21, which has been now reintroduced in the reference list. We agree with Reviewer 1 about the possibility of co-immunoprecipitating elongating CDC25A peptides associated with the ribosomal machinery. While this is a valid concern, as can be seen in the figure below (corresponding to the decropped Fig EV1), western blot analysis of the immunoprecipitate primarily detected the full-length CDC25A, with only very minor higher electrophoretic mobility signal, which can be either specific shorter forms of the protein, or non-specific signal. We believe that the low amounts of CDC25A elongating peptides are unlikely to significantly impact the results of our mass spectrometry interactome analysis.

Figure for referee with unpublished data has been removed upon request by the authors.

Point 4. **The CDC25A shift with puromycin treatment is minimal (the rationale for two exposures is unclear). The rPS6 shift is also modest. Improved experiments are needed; treating extracts with EDTA rather than puromycin might be more informative.**

While we agree that the shifts of CDC25A and RPS6 upon puromycin treatment are moderate, we would like to emphasize that they are reproducible and statistically significant (Fig 1E). Compared to the original version of the article we have now added a full quantification of RPS6 distribution across fractions (Fig 1E).

Regarding the use of two exposures, we agree that this may have created confusion and have kept the high exposure showing comparative signals between the H_2O and the puromycin treated conditions.

We chose puromycin treatment deliberately because it induces premature chain termination by entering the A site of actively translating ribosomes. This results in the dissociation of translating ribosomes, and the loss of CDC25A co-sedimentation observed under these conditions suggests that the association of the protein with the heavy fractions is dependent on active translation and intact ribosomes. In contrast, EDTA is a Mg2+ chelator that globally dissociates ribosomal subunits and destabilizes a broad range of RNA-protein interactions. This causes massive disruption of the polysome profile reflecting a more global and less specific effect. To address reviewer's 1 concern, we also performed polysomal profiling using EDTA (see figure below). This revealed a strong shift in CDC25A association with the translating machinery, which supports our initial observations and provides additional context. Taken together, these results indicate that while EDTA confirms the ribosome-dependent nature of CDC25A sedimentation, puromycin provides a more specific assessment of its association with actively translating polysomes.

Figure for referee with unpublished data has been removed upon request by the authors.

In addition, to complete this part of the study, we performed similar experiments to investigate the cosedimentation of CDC25B with the translational machinery. For this, HEK293 cells were transfected with CDC25B and processed for polysome fractionation. Western blot analysis of CDC25B in the polysomal fractions in the presence or the absence of puromycin demonstrates that, as CDC25A, CDC25B is present in the polysomal fractions. These data are now presented in the Fig EV3 D-E.

Point 5. **If the authors claim that CDC25A and CDC25B have differential effects on initiation and elongation, appropriate polysomal profiles should be presented. While elongation defects may not alter polysomal profiles, initiation defects should cause substantial 80S accumulation.**

We thank the reviewer for this insightful comment, which prompted us to provide additional polysome profiling experiments that further strengthen and clarify the respective roles of CDC25A and CDC25B in translation elongation and initiation.

As suggested, perturbations affecting translation elongation are not necessarily expected to alter polysome profiles. Indeed, elongation regulations could modulate the rate at which ribosomes transit on mRNAs rather than the efficiency of ribosome recruitment. Consequently, this results in preserved, or even slightly increased, polysome occupancy without substantial changes in the global 80S peak. Accordingly, the unchanged polysome profile observed upon the conditional expression of HA-tagged CDC25A in HEK293 cell line (Fig EV4 E,F), is consistent with its proposed function in elongation and argues against a primary defect in initiation.

We fully agree that hallmarks of initiation defects are a redistribution of ribosomes from heavy polysomes toward 80S monosomes visible in polysome profiles. Consistent with the proposed

role of CDC25B in regulating translation initiation, siRNA-mediated downregulation of CDC25B led to a marked reduction in polysome assembly that correlates with an accumulation of 80S particles (Fig EV4 C,D).

We have clarified this mechanistic distinction in the revised manuscript in the text and added the Fig EV4 C-F

Point 6. **The translation increase following short CDC25A induction is noteworthy. The authors appropriately use a mutant that cannot interact with cyclin downstream factors, demonstrating that CDC25A's stimulatory activity on translation is independent of cell cycle regulation. Consider additional experiments: Test a catalytically inactive mutant**

As indicated above (reviewer 1, point 2), we addressed reviewer's concerns by directly comparing translation upon expression of wild-type CDC25A and a catalytically inactive mutant of CDC25A. As shown in the new Fig 2C, wild type CDC25A slightly increases translation, whereas expression of the catalytically inactive C431S mutant does not produce this effect.

Analyze which mRNAs are preferentially translated upon CDC25A expression

We thank the reviewer for raising this important point regarding the potential translational regulation mediated by CDC25A.

As a first unbiased and exploratory approach to address this question, we performed polysome profiling upon CDC25A expression (new Fig. EV4 E–F), followed by RNA sequencing of polysomal fractions. We chose polysome sequencing because it represents a well-established and relevant strategy to identify translationally regulated mRNAs at the transcriptome-wide level. Importantly, although polysome profiling is classically associated with changes in translation initiation, it can also capture alterations in elongation. Significant modulation of elongation can modify ribosome density and distribution along transcripts, thereby affecting their representation in heavy versus light polysomal fractions (El-Hachem, Nat Cell Biol 2024; Guzikowski, RNA Biol 2022). Thus, this approach provides an appropriate first-level assessment of mRNAs translationally regulated upon CDC25A expression.

Bioinformatic analysis of polysome profiling following CDC25A overexpression revealed both transcriptional and translational regulation affecting several mRNAs, with a stronger effect observed at the translational level (see figure below). These observations are consistent with our previous results and suggest the existence of a pool of mRNAs whose translation may be regulated by CDC25A. Although most differences between total RNA and high-polysome fractions do not reach statistical significance, they may still reflect biologically relevant translational changes, particularly for rapid and transient regulatory events.

Further optimization of the experimental conditions, particularly with respect to timing, will therefore be required. Indeed, CDC25A is a phosphatase whose regulatory activity relies on reversible phosphorylation events that typically mediate rapid and transient cellular responses, which may limit the ability of polysome profiling to robustly capture these changes under the current experimental conditions. Complementary approaches such as Ribosome profiling (Ribo-seq) could provide higher-resolution insights into CDC25-dependent translational regulation and help precisely define the subset of regulated mRNAs. A more comprehensive analysis of the translatome regulated by CDC25A and CDC25B is currently underway in our laboratory and will be the subject of a dedicated article.

Figure for referee with unpublished data has been removed upon request by the authors.

Point 7. **Use multiple siRNAs to exclude off-target effects**
We performed RNA interference experiments with a second siRNA targeting CDC25A and CDC25B, that confirmed the data obtained in the initial manuscript. These data are now shown in Fig EV3 B-C for the effect of CDC25B depletion on translation in HEK293 cells, and in Fig EV5 A,B for the effect of CDC25A and CDC25B depletions on translation in leukemic (OCIAML3) cells.

Point 8. **Correct methods section for references, typographical errors (e.g., "supernatant"), and proper chemical formulas with subscripts**
Corrections were done as asked by reviewer.

Referee 2

We thank the Reviewer 2 for her/his interest, and for pointing out some key questions concerning the respective functions of CDC25A and B in translation initiation and elongation, and whether these functions are direct or not. We sincerely believe that answering her/his

comments, some of them being common with reviewer 1, strengthened the connections and the robustness of our datasets.

Point 1. **Fig 1D: Do endogenous CDC25A and CDC25B co-sediment with polysomes in HEK293T cells? This is important given the results of Fig 3. In this figure, please also indicate which fractions correspond to the 'monosome' and 'polysome' sections of the gradient. In addition, is it water or DMSO as indicated in parts E-F? Please, explain what EEA1 is in the main text and why it is a negative control.**

We thank the reviewer for this comment. We initially attempted to detect the endogenous proteins associated with polysomes; however, these attempts were unsuccessful. This is likely due to their low expression levels, the transient nature of their interactions, and their instability throughout the experimental procedure, particularly during precipitation steps. To overcome these technical limitations, we used overexpression systems, enabling increased detection sensitivity while maintaining temporal control of expression, and subsequently performed protein profiling analyses to assess their association under more robust and reproducible conditions. In the case of CDC25A, the inducible HEK293 doxycycline-dependent model was used, while we used transient overexpression experiments for CDC25B. Quantification of CDC25A or CDC25B, EEA1 (negative control), and RPS6 (positive control) in the polysomal and monosomal fractions indicates a redistribution of CDC25A (Fig 1E) or CDC25B (Fig EV3 D,E) from polysomal to monosomal fractions following puromycin treatment. For both figures, Fig 1E and Fig EV3 D,E, we have clearly indicated which fractions correspond to the 'monosome' and 'polysome' sections of the gradient.
The control treatment in Fig1 D-E is water and we thus corrected this error.
The description of EEA1 as a negative control is now included in the text.

Point 2, **Fig S2C: Is there any defect in proliferation beyond 24h? Normally one takes measurements through several days during exponential growth until cells have reached a plateau.**

We fully agree with referee 2 that CDC25A should increase the proliferation rates in certain conditions (moderate overexpression, and specific sub-confluent culture conditions). As shown in the figure below, this is indeed observed in our experimental conditions. After 24 hours of doxycycline-dependent CDC25A induction, the proliferation rate was not modified, whereas after 48 hours, cells overexpressing CDC25A are more proliferative. These results confirm that within the 24-hour time window used in our study, doxycycline-induced CDC25A overexpression does not affect cell proliferation. Thus, under our experimental conditions, cell cycle- or proliferation-related effects are unlikely to confound data interpretation. As all experiments presented in the manuscript were performed 24 hours post-induction, we did not include this figure in the revised manuscript.

Figure for referee with unpublished data has been removed upon request by the authors.

Point 3, **Fig 3: An important concern is that no differences in translation are observed upon depletion of CDC25A, not even in conditions where the compensatory CDC25B is absent. How is this explained? This would rather suggest indirect or non-specific functions of CDC25A over-expression.**

Our initial results, further confirmed and reinforced during the revision, indicate that CDC25B primarily regulates translation initiation, while CDC25A acts on elongation. Accordingly, the lack of effect of CDC25A depletion, even upon RNAi-mediated CDC25B knockdown, likely reflects the fact that initiation is more rate-limiting than elongation in these conditions, so any impact of CDC25A depletion is masked by the stronger effect of CDC25B depletion on initiation. Importantly, Fig 3G shows that the translation-enhancing effect of CDC25A overexpression is maintained in the absence of CDC25B. We have now added a sentence in the Discussion to highlight this point.

Point 4, **Figs 4C and S4B: It is noteworthy that depletion of the elongation factors used in these figures invariably results in decreased levels of HA-CDC25A, yet there is no effect on HA-CDC25A-mediated translation for most of them.**
Furthermore, there is no apparent decrease of translation by depletion of any of the elongation factors in the basal state (compare '-' lanes), which is unexpected.

To address the first issue, we quantified CDC25A expression in the figures indicated by the reviewer where elongation factor depletion reduced CDC25A levels in both basal and doxycycline-induced conditions. As shown below, the doxycycline-induced increase in CDC25A (-DOX/+DOX ratio) is not significantly affected, explaining the absence of impact on CDC25A-dependent translation.

Figure for referee with unpublished data has been removed upon request by the authors.

Regarding the second point, the absence of an effect of siRNA-mediated depletion of elongation factors on basal translation likely reflects the short time frame of silencing. Extended depletion is expected to impact translation more substantially. We tested this hypothesis and observed that prolonged depletion of elongation factors indeed decreases global translation, as expected.

Figure for referee with unpublished data has been removed upon request by the authors.

Given that these observations confirm our interpretation and do not alter the conclusions, we did not include additional figures in the revised manuscript.

Point 5, **Fig 4E: Please, show PLA of HA-CDC25A and another elongation factor as negative control.**
We now included PLA experiments between CDC25A and EEF2, which are presented in Fig EV4 H, and demonstrate no close proximity between these two proteins.

Point 6, **Fig 5A: Why is there a difference between 16h and 24h if CDC25A is equally well-depleted at both time points? The results suggest indirect effects of CDC25A on translation. Furthermore, the authors propose that in these cell lines there is no compensation by CDC25B. Can the authors perform a Western-blot to ensure that CDC25B levels do not change?**

While CDC25A depletion is efficient at both 16h and 24h, the effect on translation depends on downstream processes. According to our model, CDC25A primarily regulates translation elongation rather than initiation. As a result, the consequences of its depletion take time to manifest, leading to a smaller effect at 16h and a more pronounced reduction at 24h. This explanation is therefore consistent with the temporal dynamics of elongation-dependent regulation rather than indirect or non-specific effects.
Concerning the absence of CDC25B compensation, the new panel in Fig EV5 A shows that CDC25A depletion does not impact CDC25B level in leukemic cells.

Point 7, **Fig 5D and S5: It is intriguing that phosphorylation of eEF1A1 is not affected by CDC25A depletion, while phosphorylation of eEF1D is. This does not fit with the proposed roles of these factors in mediating CDC25A function in translation in HEK293 cells, as per the results of Figure 4. In these cells, the authors propose that eEF1A1 mediates translation regulation by CDC25A, while eEF1D does not. Perhaps these contradictions are rooted in the use of different cell lines, and therefore the authors may want to repeat their translation experiments (polysome profiles, potential role of CDC25B) in the leukemic cell lines.**

We agree with the reviewer that this discrepancy required clarification. The absence of an effect of EEF1D depletion in our initial experiments was likely due to suboptimal silencing conditions. Upon optimization, we found that EEF1D depletion, similarly to EEF1A1 depletion, abolishes CDC25A-induced translational enhancement (new Fig 4E,F). Consistently, PLA analyses showed that CDC25A is in close proximity to both EEF1A1 and EEF1D (Fig 4G,H), in agreement with their known association within the same elongation complex. We thank the reviewer for prompting us to revisit this point, which strengthens our conclusions regarding CDC25A-mediated translational regulation.

Concerning the differential phosphorylation pattern, two elements should be considered. First, although a phosphorylated EEF1A1 peptide was detected, its abundance was ~100-fold lower than that of phosphorylated EEF1D, suggesting low stoichiometry or detection limitations. Second, an increase in EEF1A1 phosphorylation upon CDC25A depletion was

observed in one replicate, indicating a possible trend but preventing firm conclusions. Further analyses (e.g., optimized phosphopeptide enrichment or targeted mass spectrometry) will be required to address this point, which lies beyond the scope of the present study.

These results are now presented in a new version of Fig 4 (E and F), in which we show the impact of EEF1D depletion after 48 hours, and Fig 4H for the PLA experiments showing CDC25A/EEF1D proximity.
As a last additional point, we also added the result of experiments showing that CDC25A overexpression does not modify the protein level of EEF1D (Fig EV4 J), as we already did for EEF1A1 in the initial version of the manuscript (new Fig EV4 K,L)

Point 8, **Given the contradictions, the discussion is highly speculative.**

We modified different points of the discussion in order to reduce too speculative conclusions

Minor points:
A scheme of how the factors evaluated in Fig 4 are involved in translation would help the reader.
A scheme of the EEF1D1 and EEF1A1 complexes acting during elongation has been added in Fig EV4 G.

Fig S1 has only one panel, not necessary to name a panel here.
Molecular weight markers should be shown in all Western blots.
Make sure to correctly show the formula of water.
These corrections were done

Referee 3

Point 1**, Their claims notwithstanding, the data in Figure 1D show very clearly that the bulk of CDC25A is not associated with translating ribosomes in the polysomal fractions. The pattern is not even remotely similar to that of RPS6, which (for RPS6) shows most of it in polysomes. While this result does not preclude a role for CDC25A in protein synthesis, it makes the connection more tenuous.**

We thank the reviewer for raising this important point. We agree that, in Fig 1D (initial version), the proportion of CDC25A detected in monosomal and polysomal fractions is lower than that of RPS6. In many polysome profiling studies, ribosomal proteins are used as positive controls. However, such proteins are structural components of the ribosome and are therefore expected to be predominantly associated with polysomes. In contrast, CDC25A is a regulatory factor, and its association with translating ribosomes is likely to involve only a

subpopulation of the total protein. Direct comparison with a structural ribosomal protein may therefore underestimate the functional relevance of its cosedimentation. Several factors may further limit CDC25A/B detection in polysomal fractions, including their relatively low expression level, the transient nature of its interaction with the translation machinery, and potential instability during polysome preparation. To address these limitations, we complemented our analysis using inducible overexpression of CDC25A. Under these conditions, we observed a clear and puromycin-sensitive association of CDC25A (Fig 1D,E) and CDC25B (Fig EV3 D,E) with the translational machinery, supporting a specific interaction with actively translating ribosomes.

<u>Point 2</u>**, The results in Figure 2 support a role for CDC25A in promoting protein synthesis, independently of CDC25A's role in the cell cycle (because the R446L mutant that does not interact with the CDK had no effect). But this conclusion relies exclusively on over-expression studies. The effect is also relatively modest (~20-30%) in this experiment. Although the authors invoke a compensatory role for CDC25B to explain the lack of any effect in the converse experiment with the siRNA to lower CDC25A (Figure 3), it leaves the issue open.**

We were indeed limited in our demonstration because of the compensation by CDC25B of CDC25A depletion effects. However, the use of the R446L mutant was not the only evidence that CDC25A function is cell cycle and CDKs independent, since we also presented in Fig EV2 D experiments with CDKs pharmacological inhibitors that do not reverse CDC25A overexpression impact on translation. We also demonstrate that in leukemic cells, siRNA-mediated CDC25A depletion decreases puromycine incorporation. In the new version of the manuscript, these results are now confirmed with a second independent siRNA, both for CDC25A and CDC25B (Fig EV3 B,C and Fig EV5 A,B).
Finally, quantifications showing the significance of CDC25A localization in polysomal fractions (Fig 1E), and the new experiments showing that CDC25A overexpression does not modify the polysome profile of HEK293 cells, in good agreement with its implication in the elongation steps of translation, constitute important additional informations further strengthening our initial conclusions.

<u>Point 3</u>**, The authors identify several proteins with roles in protein synthesis that have altered phosphorylation in CDC25 perturbations. This is fine, and a good starting point to understand and solidify their previous observations. However, they show no evidence that any of these readouts could explain the purported role of CDC25 in translation. Couldn't they functionally test the role of any of these sites in the CDC25-dependent control of protein synthesis? Along the same lines, an important control that is missing here is a test that the PPase role of CDC25 is necessary. How does a catalytically dead version of CDC25A behave in these experiments?**

Identifying the molecular targets (substrates) of CDC25A that could mediate its function in translation may need a complete study specifically dedicated to this question. However, the new results that we added concerning the implication of EEF1D downstream of CDC25A (Fig 4) open an interesting way of investigation, since EEF1D phosphorylation was found increased in the phosphoproteome in response to CDC25A depletion. Concerning the importance of CDC25A catalytic activity, new experiments with a catalytically dead mutant of CDC25A (C431S) have been performed and included into the manuscript. Overexpression of this mutant version of CDC25A in HEK293 cells does not modify the translation rate (sunset experiments), while the wild type version of the phosphatase does. These new results are presented in Fig 2 C.

1st Revision - Editorial Decision 24th Apr 2026

Dear Dr. Cammas,

Thank you for the submission of your revised manuscript. We have now received the enclosed reports from the referees, as well as referee cross-comments. As you will see, referee 3 is not satisfied with the revised ms. However, both referees 1 and 2 agree that the remaining concerns can be addressed and that your study can be accepted for publication. Can you please address all remaining concerns also in a point-by-point response to be submitted with your final ms files.

A few editorial requests will also need to be addressed before we can proceed with the official acceptance:

- Your ms currently has 5 main figures but the results and discussion sections are separate. Please either add one more main figure (to publish your ms as a full article) or combine the results and discussion sections to publish your ms as a short report. You can find more information about our article types in our guide to authors online.

- Please correct the conflict of interest subheading to "Disclosure and Competing Interests Statement" and place it after the Acknowledgments.

- The author credits need to be removed from the ms file. All credits need to be entered during online ms submission.

- The FUNDING INFO is not congruent; Ligue Contre le Cancer (laliguecancer) is in our online submission system, but it is missing from the Acknowledgments; the funders in the Comments box online need to be removed from the box and each funder should be provided as a separate entry (More Funders). There is a typo in this section in the ms: "Aknowledgements".

- Please upload all main and EV figures as individual production quality Figure files.

- There are 3 EV tables uploaded, but Table EV1 and Table EV2 are Datasets and need to be updated to Dataset EV1 and Dataset EV2 in all places (source file names, legends, titles in the system, callouts in the ms). The content of Table EV3 should be included in the Reagents & Tools table.

- Please add the URLs for the large deposited datasets provided in the Data Availability Section, PXD066262 and PXD066447.

- Materials and Methods should be just Methods.

* Figure Legends - Comments *

- Please note that the exact p values are not provided in the legends of figures 4G, H; 5B, EV4 H, please provide exact values as reasonable.
- Please indicate the statistical test used for data analysis in the legends of figures 1A, B
- Please note that for the figures 2D, E; 3F, G; 4A-H; 5B; EV2 A-C; EV3 A, C, F, G, H; EV4 B, H, I p-values and statistical tests are indicated in the legends. However, comparison for the same, ""****/***/**/*"" has not been represented in the figures. Please rectify this in the figures or legends as applicable.

I would like to suggest some minor changes to the abstract that needs to be written in present tense. Please let me know if you agree with this and if my changes are OK:

Molecular and functional networks driving coordination between cell cycle and mRNA translation remain to be explored. Here, we use mass spectrometry-based proteomics to comprehensively investigate the interactome and phosphoproteome of the cell cycle regulator CDC25A. We identify actors of mRNA regulation, such as RNA-binding proteins and translation factors, as interacting partners of CDC25A. CDC25A overexpression increases global translation, whereas catalytic inactivation or pharmacological inhibition decreases protein synthesis. A Cyclin Dependent Kinase (CDK) interaction-deficient mutant of CDC25A also enhances translation, indicating a CDK-independent role. Our results further reveal an interplay between CDC25A and CDC25B whereby down-regulation of CDC25A leads to compensatory overexpression of CDC25B. The roles of CDC25A and CDC25B in mRNA translation are independent of their roles in the cell cycle [OK?], with CDC25A possibly regulating translation elongation and CDC25B rather involved in initiation. In acute myeloid leukemia cells, CDC25A depletion also inhibits translation suggesting its potential relevance as a therapeutic target. We propose that CDC25 phosphatases might be signaling platforms coordinating cell cycle progression with protein synthesis.

EMBO press papers are accompanied online by A) a short (1-2 sentences) summary of the findings and their significance, B) 2-3 bullet points highlighting key results and C) a synopsis image that is exactly 550 pixels wide and 300 pixels high. The synopsis

image should provide a sketch of the major findings, like a graphical abstract. Please note that text needs to be readable at the final size. Please send us this information along with the final manuscript.

I look forward to seeing a final version of your manuscript as soon as possible.

Best regards,
Esther

Esther Schnapp, PhD
Senior Editor
EMBO reports

Referee #1:

The manuscript is improved and the authors have addressed all my concerns. My compliments for this brave work, as addressing the role of cdc25 on mRNA translation is certainly not trivial, given the known roles of the protein.

Referee #2:

The authors have shown new polysome analyses and elongation factor assessment that add credibility to the conclusions. In this respect, it would be convenient to include the polysome profiling analysis in the presence of EDTA requested by reviewer #1 to the manuscript. Although puromycin addition to polysome profiles is more specific to translation, the reduction in polysomes that the authors get in their experiments is small and, therefore, the expected shifts in CDC25A and RPs are also small. The EDTA experiment, even though less specific than puromycin addition, produces a stronger effect and lends further credibility to the conclusions.
Other comments:

- New data in EV3D: The authors argue that CDC25B associates to polysomes similarly to CDC25A. While this would be expected for CDC25A given its proposed role in translation elongation, this is unexpected for CDC25B given its proposed role in translation initiation through regulation of the mTOR pathway and eIF2-alpha phosphorylation. Data in EV3D needs to be quantified and, if significant, an explanation for this conundrum should be discussed.
- Summary, last sentence: 'mRNA translation' and 'protein synthesis' are the same process. Please remove one of those.
- Page 5, new blue text: The authors conclude that over-expression of the wild type phosphatase increases translation in this experiment, but the difference with the empty vector lane shows no statistical significance (Fig 2C). Please, modify the text to acknowledge that there is a significant difference between the two constructs even though the increase in translation by overexpression of wild type CDC25A was just a tendency in this experiment. Otherwise, perform more experiments to achieve such significance.
- Page 5: 'whether' missing in the first sentence of the new text in blue.

Referee #3:

The authors only partially addressed my previous points.
The purported association of CDC25A with ribosomes remains unconvincing. The picture from the new experiment with overexpression (which, of course, is not ideal because one cannot conclude much from ectopic expression studies) is not that different from what they showed in Figure 1D,E. There is another issue with that figure. They report a 25%-30% association between CDC25A and polysomes (Figure 1D). I quantified their blot, and this is not true. The way they must have calculated it is by excluding the first (F5) fraction, focusing only on the monosome and polysome fractions. This is misleading. If they include that first fraction (probably the bulk free CDC25A), then the true association of CDC25A with polysomes will be negligible. These problems, in my opinion, reduce the significance of the work.
I had suggested that they validate one (any) target among those they identified, to boost confidence in the phenomenon. EEF1D may be downstream, but the work is too preliminary, and the authors argue that more work would be beyond the scope of the study. Perhaps. I guess this is something for the editor to decide.
On the positive side, they included a new experiment using a catalytically dead CDC25A mutant (C431S) in Figure 2C, which supports the conclusion.
Overall, the picture that emerges is mixed. I think that there is something there, but the effect is either too small to be biologically significant or the authors did not pursue it further to strengthen the conclusions. Hence, I am ambivalent about the work and my enthusiasm is limited.

Cross-comments from referee 1:

I would be happy enough to have a fair discussion of the limits of this work, accept the suggestion that CDC25 phosphatase has a moonlighting function, as long as the authors provide an answer to the specific issue on the quantitation.

Cross-comments from referee 2:

It seems to me that Referee #3 is being a bit tough regarding the experiment shown in Fig 1D. I agree that the changes in the distribution of CDC25A upon puromycin treatment are small, but this experiment suffers from a poor effect of puromycin treatment in general, as judged by the small redistribution of the ribosomal protein RPS6. The quantification of monosome/polysome (as opposed of total/polysome as suggested by the reviewer) is a standard one. In that quantification, one can see that CDC25A follows a similar pattern than RPS6 and different to EEA1.
Having said this, the polysome profiling shown in Fig 1D of the revised version seems exactly the same to me than Fig1D in the new version. The only new thing added is the quantification of RPS6. The new polysome profiling added in support of the conclusions is for CDC25B (shown in Fig EV3D-E).
The EDTA experiment provided in response to reviewer #1 lends further support and the results are more dramatic, and this is why I se¡uggested to include this experiment in the paper.

EMBOR-2025-62426V3 : **Novel CDK-independent function of CDC25 phosphatases in mRNA translation**

Shin et al.

We are grateful to the editor and reviewers for their comments and suggestions that contributed to clarify some remaining concerns. For clarity's sake, the modifications in the revised manuscript are indicated in orange.

Editorial requests

Point1. **Your ms currently has 5 main figures but the results and discussion sections are separate. Please either add one more main figure (to publish your ms as a full article) or combine the results and discussion sections to publish your ms as a short report. You can find more information about our article types in our guide to authors online.**

We have now split the previous Figure 4 into two separate figures (New Fig 4 and Fig 5) and Figure EV4 into two separate figures (New Fig EV4 and EV5), highlighting the two distinct mechanisms by which CDC25A and CDC25 regulate translation elongation and initiation, respectively.

Point2. **Please correct the conflict of interest subheading to "Disclosure and Competing Interests Statement" and place it after the Acknowledgments.**

The mentioned subheading has been corrected and displaced after the acknowledgement.

Point3. **The author credits need to be removed from the ms file. All credits need to be entered during online ms submission.**

The author contributions have been removed from the manuscript and entered online.

Point4. **The FUNDING INFO is not congruent; Ligue Contre le Cancer (laliguecancer) is in our online submission system, but it is missing from the Acknowledgments; the funders in the Comments box online need to be removed from the box and each funder should be provided as a separate entry (More Funders). There is a typo in this section in the ms: "Aknowledgements".**

La Ligue Contre le Cancer is now indicated in the Acknowledgments; the funders are provided online as a separate entry and the typo error in the ms has been corrected.

Point5. **Please upload all main and EV figures as individual production quality Figure files.**

Each main and EV Figures were uploaded as individual quality Figure files

Point6. **There are 3 EV tables uploaded, but Table EV1 and Table EV2 are Datasets and need to be updated to Dataset EV1 and Dataset EV2 in all places (source file names, legends, titles in the system, callouts in the ms). The content of Table EV3 should be included in the Reagents & Tools table.**

Tables EV1 and EV2 are mentioned as Dataset EV1 and EV2 and the Table EV3 is included in the Reagents & Tools table.

Point7. **Please add the URLs for the large deposited datasets provided in the Data Availability Section, PXD066262 and PXD066447.**

The URLs are now included in the data availability section.

Point8. **Materials and Methods should be just Methods.**

This title for Methods section has been renamed.

Point9. **Please note that the exact p values and the statistical test are not provided in some legends.**
The exact p values for figures 4G, H; 5B, EV4 H and the statistical test used for data analysis in the legends of figures 1A, B have been incuded.

Point10. **Please note that for the figures 2D, E; 3F, G; 4A-H; 5B; EV2 A-C; EV3 A, C, F, G, H; EV4 B, H, I p-values and statistical tests are indicated in the legends. However, comparison for the same, ""****/***/**/*"" has not been represented in the figures. Please rectify this in the figures or legends as applicable.**

We have now included the ""****/***/**/*" next to the exact p-values in the figures

Point11. **I would like to suggest some minor changes to the abstract that needs to be written in present tense.**

We agree with the suggested changes to the abstract and have now modified it accordingly.

Point12. **EMBO press papers are accompanied online by A) a short (1-2 sentences) summary of the findings and their significance, B) 2-3 bullet points highlighting key results and C) a synopsis image that is exactly 550 pixels wide and 300 pixels high. The synopsis image should provide a sketch of the major findings, like a graphical abstract. Please note that text needs to be readable at the final size. Please send us this information along with the final manuscript.**

The following informations have been sent with the final manuscript:

A) a short (1-2 sentences) summary of the findings and their significance: CDC25A and CDC25B phosphatases regulate mRNA translation elongation and initiation, respectively. This occurs independently of their well-established functions of Cyclin-Dependent Kinases regulation during the different phases of the cell cycle, revealing a moonlighting role for CDC25 proteins in RNA expression regulation.

B) 2-3 bullet points highlighting key results:
- CDC25A protein interactome underscores its role in RNA regulation
- CDC25A and CDC25B regulate mRNA translation elongation and initiation by modulating elongation factors and signaling pathways, respectively.
- Translational regulation by CDC25A and CDC25B is intertwined, CDK-independent, and occurs across the cell cycle.

C) a synopsis image:

Uploaded

Referee #2:

Point 1. **The authors have shown new polysome analyses and elongation factor assessment that add credibility to the conclusions. In this respect, it would be convenient to include**

the polysome profiling analysis in the presence of EDTA requested by reviewer #1 to the manuscript. Although puromycin addition to polysome profiles is more specific to translation, the reduction in polysomes that the authors get in their experiments is small and, therefore, the expected shifts in CDC25A and RPs are also small. The EDTA experiment, even though less specific than puromycin addition, produces a stronger effect and lends further credibility to the conclusions.

As suggested by the referee 2, the polysome profiling experiment following EDTA treatment supporting the co-sedimentation of CDC25A with translating polyribosomes is now included in the Figure EV1 B.

Other comments:

Point 1. **New data in EV3D: The authors argue that CDC25B associates to polysomes similarly to CDC25A. While this would be expected for CDC25A given its proposed role in translation elongation, this is unexpected for CDC25B given its proposed role in translation initiation through regulation of the mTOR pathway and eIF2-alpha phosphorylation. Data in EV3D needs to be quantified and, if significant, an explanation for this conundrum should be discussed.**

Quantification asked by reviewer 2 is presented in figure EV3 E. Although we cannot measure the statistical significance for this n=2 experiment, we observed a clear tendency of CDC25B protein to shift from heavy to light polysomal fractions in response to puromycin treatment.

The observation that CDC25B impacts on 4EBP1, RPS6 and RPS6K phosphorylation is fully consistent with its co-sedimentation with polysomes, since translational control by signaling pathways may occur at the level of ribosome-associated (polysomal) mRNAs rather than being restricted to a purely soluble cytosolic compartment (Holz et al. (Cell, 2005)). eIF2 also participates to protein complexes binding to mRNA at the initiation steps of translation, implicating potential localization of this protein in light or heavy polysomal fractions (see for instance https://pubmed.ncbi.nlm.nih.gov/1361233/). In addition, as evidenced by the CDC25A interactome, additional factors may connect CDC25B to polysomes, the characterization of which in future studies will be required to fully address this question.

Point 2. **Summary, last sentence: 'mRNA translation' and 'protein synthesis' are the same process. Please remove one of those.**

The abstract has been modified following the editor’s suggestions including this last sentence.

Point 3. **Page 5, new blue text: The authors conclude that over-expression of the wild type phosphatase increases translation in this experiment, but the difference with the empty vector lane shows no statistical significance (Fig 2C). Please, modify the text to acknowledge**

that there is a significant difference between the two constructs even though the increase in translation by overexpression of wild type CDC25A was just a tendency in this experiment. Otherwise, perform more experiments to achieve such significance.

As asked by reviewer 2 we modified the text to better point to the difference between the wild type and the catalytically dead versions of CDC25A overexpression, and we concluded that the catalytic activity is essential for the new functions of CDC25A in mRNA translation

Point 4. **Page 5: 'whether' missing in the first sentence of the new text in blue.**

This correction was done

Referee #3:

Point 1. **The purported association of CDC25A with ribosomes remains unconvincing. The picture from the new experiment with overexpression (which, of course, is not ideal because one cannot conclude much from ectopic expression studies) is not that different from what they showed in Figure 1D,E. There is another issue with that figure. They report a 25%-30% association between CDC25A and polysomes (Figure 1D). I quantified their blot, and this is not true. The way they must have calculated it is by excluding the first (F5) fraction, focusing only on the monosome and polysome fractions. This is misleading. If they include that first fraction (probably the bulk free CDC25A), then the true association of CDC25A with polysomes will be negligible. These problems, in my opinion, reduce the significance of the work.**

We thank the reviewer for this important point regarding the quantification strategy. Our analysis was intentionally designed to assess the redistribution of CDC25A across ribosome-associated fractions rather than to determine its absolute abundance relative to the total cellular pool. This quantification approach was selected because, as stated by Reviewer 1, it is routinely used in the field. As indicated in Figure 1D and indicated in the text, the reported 25–30% change specifically reflects the redistribution of CDC25A within ribosome-associated fractions (monosomes and polysomes) and was calculated to evaluate its relative distribution across translating ribosomal complexes. We agree that inclusion of the free cytosolic fraction would substantially reduce the proportion of total cellular CDC25A associated with ribosomes, as expected for a regulatory protein involved in translation rather than for a constitutive component of the translational machinery. This observation is consistent with CDC25 functioning as a moonlighting protein rather than as an intrinsic component of the translational machinery, as is the case for RPS6.

To further strengthen this conclusion, and as suggested by referee 2, we have added a cosidementation experiment following EDTA treatment, which provides additional support for the association of CDC25A with polysomes.

Point 2. **I had suggested that they validate one (any) target among those they identified, to boost confidence in the phenomenon. EEF1D may be downstream, but the work is too preliminary, and the authors argue that more work would be beyond the scope of th<e study. Perhaps. I guess this is something for the editor to decide.**

On the positive side, they included a new experiment using a catalytically dead CDC25A mutant (C431S) in Figure 2C, which supports the conclusion.
Overall, the picture that emerges is mixed. I think that there is something there, but the effect is either too small to be biologically significant or the authors did not pursue it further to strengthen the conclusions. Hence, I am ambivalent about the work and my enthusiasm is limited.

We understand the reviewer's mixed impression, which is largely driven by the relatively modest magnitude of the observed effects. However, it is important to emphasize that our conclusions do not rely on a single experimental readout, but rather on a convergent set of independent observations that collectively support the main message of the study. These include the CDC25A interactome, which reveals an enrichment in RNA-binding proteins and, more specifically, factors involved in translation; the impact on nascent protein synthesis; the co-sedimentation of CDC25A and CDC25B with the translational machinery in a polysome integrity-dependent manner; the functional interplay between CDC25A and CDC25B; and the identification of both physical and functional downstream targets of these phosphatases. Importantly, key controls highlighted by the reviewer, including the catalytically inactive mutant, further strengthen the specificity and mechanistic relevance of these observation

It is worth noting that modest but significant effects are fully consistent with a regulatory role for CDC25A in translation. Previous studies have shown that translational phosphatases primarily act as context-dependent regulatory factors whose interactions with translation complexes are often transient rather than constitutive or structural. Within this framework, modest but reproducible effects are commonly observed for signaling phosphatases involved in translational control and are therefore consistent with a regulatory, rather than structural, role in translation.

Concerning the scope of the work, we appreciate the reviewer's suggestion to validate individual candidates such as EEF1D to further strengthen confidence in the observed phenomenon. However, while we agree that such validation could be informative, focusing on a single target would provide limited mechanistic resolution given the transient and dynamic nature of the regulatory events involved. A meaningful mechanistic validation would instead require a broader and systematic approach, including interrogation of the CDC25A interactome and RNA-binding protein landscape, as well as potential interplay with CDC25B. These directions constitute a substantial body of work that would extend our study, although we believe that

they are not necessary to support the main conclusion of the present work, which is the identification of a novel functional link between CDC25 phosphatases and the regulation of translation.

Cross-comments from referee 1:

Point 1. **I would be happy enough to have a fair discussion of the limits of this work, accept the suggestion that CDC25 phosphatase has a moonlighting function, as long as the authors provide an answer to the specific issue on the quantitation.**

As suggested, we added both the limits of the work and the notion of the moonlighting function of CDC25 in the discussion.

We have clarified above that our analysis was specifically designed to assess the redistribution of CDC25A upon puromycin treatment, rather than to determine its absolute abundance within the total cytosolic pool. This approach, which Reviewer 3 also noted as being standard (see below), was chosen to specifically capture changes in association with ribosome-containing fractions and therefore directly address the dynamic regulation of CDC25A within translationally active complexes.

Cross-comments from referee 2:

Point 2. **It seems to me that Referee #3 is being a bit tough regarding the experiment shown in Fig 1D. I agree that the changes in the distribution of CDC25A upon puromycin treatment are small, but this experiment suffers from a poor effect of puromycin treatment in general, as judged by the small redistribution of the ribosomal protein RPS6. The quantification of monosome/polysome (as opposed of total/polysome as suggested by the reviewer) is a standard one. In that quantification, one can see that CDC25A follows a similar pattern than RPS6 and different to EEA1.**
Having said this, the polysome profiling shown in Fig 1D of the revised version seems exactly the same to me than Fig1D in the new version. The only new thing added is the quantification of RPS6. The new polysome profiling added in support of the conclusions is for CDC25B (shown in Fig EV3D-E).
The EDTA experiment provided in response to reviewer #1 lends further support and the results are more dramatic, and this is why I se¡uggested to include this experiment in the paper.

We thank the reviewers for their comments concerning our standard approach to quantifying CDC25 co-sedimentation. We have now incorporated the EDTA dissociation experiment in the revised manuscript (Fig EV 1B). This experiment shows a more pronounced redistribution of CDC25A upon ribosome disruption, further strengthening the conclusion that CDC25A associates with polysomes.

Dr. Anne Cammas
INSERM UMR 1037
CRCT
Toulouse 31000
France

Dear Anne,

I am very pleased to accept your manuscript for publication in the next available issue of EMBO reports. Thank you for your contribution to our journal.

Your manuscript will be processed for publication by EMBO Press. It will be copy edited and you will receive page proofs prior to publication. Please note that you will be contacted by Springer Nature Author Services to complete licensing and payment information.

You may qualify for financial assistance for your publication charges - either via a Springer Nature fully open access agreement or an EMBO initiative. Check your eligibility: https://link.springer.com/journal/44319/how-to-publish-with-us

Should you be planning a Press Release on your article, please notify the production team via this form in order to coordinate publication and release dates: https://forms.cloud.microsoft/e/eH4A8JVP6g

If you have any questions, please do not hesitate to contact the Editorial Office. Thank you for your contribution to EMBO Reports.

Best regards,
Esther

Esther Schnapp, PhD
Senior Editor
EMBO reports

>>> Please note that it is EMBO Reports policy for the transcript of the editorial process (containing referee reports and your response letter) to be published as an online supplement to each paper. If you do NOT want this, you will need to inform the Editorial Office via email immediately. More information is available here: https://link.springer.com/partners/embo-press/editorial-policies#Peer%20review

EMBO Press Author Checklist

Corresponding Author Name: Anne CAMMAS; Stéphane MANENTI
Journal Submitted to: EMBO Reports
Manuscript Number: EMBOR-2025-62426V1

USEFUL LINKS FOR COMPLETING THIS FORM
The EMBO Journal - Author Guidelines
EMBO Reports - Author Guidelines
Molecular Systems Biology - Author Guidelines
EMBO Molecular Medicine - Author Guidelines

Reporting Checklist for Life Science Articles (updated January
This checklist is adapted from Materials Design Analysis Reporting (MDAR) Checklist for Authors. MDAR establishes a minimum set of requirements in transparent reporting in the life sciences (see Statement of Task: 10.31222/osf.io/9sm4x). Please follow the journal's guidelines in preparing your
Please note that a copy of this checklist will be published alongside your article.

Abridged guidelines for figures
1. Data
The data shown in figures should satisfy the following conditions:
- the data were obtained and processed according to the field's best practice and are presented to reflect the results of the experiments in an accurate and unbiased manner.
- ideally, figure panels should include only measurements that are directly comparable to each other and obtained with the same assay.
- plots include clearly labeled error bars for independent experiments and sample sizes. Unless justified, error bars should not be shown for technical
- if n<5, the individual data points from each experiment should be plotted. Any statistical test employed should be justified.
- Source Data should be included to report the data underlying figures according to the guidelines set out in the authorship guidelines on Data

2. Captions
Each figure caption should contain the following information, for each panel where they are relevant:
- a specification of the experimental system investigated (eg cell line, species name).
- the assay(s) and method(s) used to carry out the reported observations and measurements.
- an explicit mention of the biological and chemical entity(ies) that are being measured.
- an explicit mention of the biological and chemical entity(ies) that are altered/varied/perturbed in a controlled manner.
- the exact sample size (n) for each experimental group/condition, given as a number, not a range;
- a description of the sample collection allowing the reader to understand whether the samples represent technical or biological replicates (including how many animals, litters, cultures, etc.).
- a statement of how many times the experiment shown was independently replicated in the laboratory.
- definitions of statistical methods and measures:
  - common tests, such as t-test (please specify whether paired vs. unpaired), simple χ2 tests, Wilcoxon and Mann-Whitney tests, can be unambiguously identified by name only, but more complex techniques should be described in the methods section;
  - are tests one-sided or two-sided?
  - are there adjustments for multiple comparisons?
  - exact statistical test results, e.g., P values = x but not P values < x;
  - definition of 'center values' as median or average;
  - definition of error bars as s.d. or s.e.m.

Please complete ALL of the questions below.
Select "Not Applicable" only when the requested information is not relevant for your study.

Materials

Newly Created Materials	Information included in the manuscript?	In which section is the information available? (Reagents and Tools Table, Materials and Methods, Figures, Data Availability Section)
New materials and reagents need to be available; do any restrictions apply?	Not Applicable	

Antibodies	Information included in the manuscript?	In which section is the information available? (Reagents and Tools Table, Materials and Methods, Figures, Data Availability Section)
For **antibodies** provide the following information:<br>- Commercial antibodies: RRID (if possible) or supplier name, catalogue number and or/clone number<br>- Non-commercial: RRID or citation	Yes	Reagents and tools table and material and methods section

DNA and RNA sequences	Information included in the manuscript?	In which section is the information available? (Reagents and Tools Table, Materials and Methods, Figures, Data Availability Section)
**Short novel DNA or RNA including primers, probes**: provide the sequences.	Yes	Reagents and tools table, materials and methods, Table EV3

Cell materials	Information included in the manuscript?	In which section is the information available? (Reagents and Tools Table, Materials and Methods, Figures, Data Availability Section)
**Cell lines:** Provide species information, strain. Provide accession number in repository **OR** supplier name, catalog number, clone number, and/**OR** RRID.	Yes	Reagents and tools table
**Primary cultures:** Provide species, strain, sex of origin, genetic modification status.	Not Applicable	
Report if the cell lines were recently **authenticated** (e.g., by STR profiling) and tested for mycoplasma contamination.	Not Applicable	

Experimental animals	Information included in the manuscript?	In which section is the information available? (Reagents and Tools Table, Materials and Methods, Figures, Data Availability Section)
**Laboratory animals or Model organisms:** Provide species, strain, sex, age, genetic modification status. Provide accession number in repository **OR** supplier name, catalog number, clone number, **OR** RRID.	Not Applicable	
**Animal observed in or captured from the field:** Provide species, sex, and age where possible.	Not Applicable	
Please detail **housing and husbandry conditions**.	Not Applicable	

Plants and microbes	Information included in the manuscript?	In which section is the information available? (Reagents and Tools Table, Materials and Methods, Figures, Data Availability Section)
**Plants:** provide species and strain, ecotype and cultivar where relevant, unique accession number if available, and source (including location for collected wild specimens).	Not Applicable	
**Microbes:** provide species and strain, unique accession number if available, and source.	Not Applicable	

Human research participants	Information included in the manuscript?	In which section is the information available? (Reagents and Tools Table, Materials and Methods, Figures, Data Availability Section)
If collected and within the bounds of privacy constraints report on age, sex and gender or ethnicity for all study participants.	Not Applicable	

Core facilities	Information included in the manuscript?	In which section is the information available? (Reagents and Tools Table, Materials and Methods, Figures, Data Availability Section)
If your work benefited from core facilities, was their service mentioned in the acknowledgments section?	Yes	They are co-authors

Design

Study protocol	Information included in the manuscript?	In which section is the information available? (Reagents and Tools Table, Materials and Methods, Figures, Data Availability Section)
If study protocol has been **pre-registered, provide DOI in the manuscript**. For clinical trials, provide the trial registration number **OR** cite DOI.	Not Applicable	
Report the **clinical trial registration number** (at ClinicalTrials.gov or equivalent), where applicable.	Not Applicable	

Laboratory protocol	Information included in the manuscript?	In which section is the information available? (Reagents and Tools Table, Materials and Methods, Figures, Data Availability Section)
Provide DOI OR other citation details if **external detailed step-by-step protocols** are available.	Not Applicable	

Experimental study design and statistics	Information included in the manuscript?	In which section is the information available? (Reagents and Tools Table, Materials and Methods, Figures, Data Availability Section)
Include a statement about **sample size** estimate even if no statistical methods were used.	Yes	Materials and methods
Were any steps taken to minimize the effects of subjective bias when allocating animals/samples to treatment (e.g. **randomization procedure**)? If yes, have they been described?	Not Applicable	
Include a statement about **blinding** even if no blinding was done.	Not Applicable	
Describe **inclusion/exclusion criteria** if samples or animals were excluded from the analysis. Were the criteria pre-established?<br><br>If sample or data points were omitted from analysis, report if this was due to attrition or intentional exclusion and provide justification.	Not Applicable	
For every figure, are **statistical tests** justified as appropriate? Do the data meet the assumptions of the tests (e.g., normal distribution)? Describe any methods used to assess it. Is there an estimate of variation within each group of data? Is the variance similar between the groups that are being statistically compared?	Yes	Materials and methods

Sample definition and in-laboratory replication	Information included in the manuscript?	In which section is the information available? (Reagents and Tools Table, Materials and Methods, Figures, Data Availability Section)
In the figure legends: state number of times the experiment was **replicated** in laboratory.	Yes	In figure caption
In the figure legends: define whether data describe **technical or biological replicates**.	Yes	In figure caption

Ethics

Ethics	Information included in the manuscript?	In which section is the information available? (Reagents and Tools Table, Materials and Methods, Figures, Data Availability Section)
Studies involving **human participants**: State details of **authority granting ethics approval** (IRB or equivalent committee(s), provide reference number for approval.	Not Applicable	
Studies involving **human participants**: Include a statement confirming that **informed consent** was obtained from all subjects and that the experiments conformed to the principles set out in the WMA Declaration of Helsinki and the Department of Health and Human Services Belmont Report.	Not Applicable	
Studies involving **human participants:** For publication of **patient photos**, include a statement confirming that consent to publish was obtained.	Not Applicable	
Studies involving experimental **animals**: State details of **authority granting ethics approval** (IRB or equivalent committee(s), provide reference number for approval. Include a statement of compliance with ethical regulations.	Not Applicable	
Studies involving **specimen and field samples:** State if relevant **permits** obtained, provide details of authority approving study; if none were required, explain why.	Not Applicable	

Dual Use Research of Concern (DURC)	Information included in the manuscript?	In which section is the information available? (Reagents and Tools Table, Materials and Methods, Figures, Data Availability Section)
Could your study fall under dual use research restrictions? Please check biosecurity documents and list of **select agents and toxins** (CDC): https://www.selectagents.gov/sat/list.htm	Not Applicable	
If you used a select agent, is the security level of the lab appropriate and reported in the manuscript?	Not Applicable	
If a study is subject to dual use research of concern regulations, is the name of the **authority granting approval and reference number** for the regulatory approval provided in the manuscript?	Not Applicable	

Reporting

The MDAR framework recommends adoption of discipline-specific guidelines, established and endorsed through community initiatives. Journals have their own policy about requiring specific guidelines and recommendations to complement MDAR.

Adherence to community standards	Information included in the manuscript?	In which section is the information available? (Reagents and Tools Table, Materials and Methods, Figures, Data Availability Section)
State if relevant guidelines or checklists (e.g., **ICMJE, MIBBI, ARRIVE, PRISMA**) have been followed or provided.	Not Applicable	
For **tumor marker prognostic studies,** we recommend that you follow the **REMARK** reporting guidelines (see link list at top right). See author guidelines, under 'Reporting Guidelines'. Please confirm you have followed these guidelines.	Not Applicable	
For **phase II and III randomized controlled trials**, please refer to the **CONSORT** flow diagram (see link list at top right) and submit the CONSORT checklist (see link list at top right) with your submission. See author guidelines, under 'Reporting Guidelines'. Please confirm you have submitted this list.	Not Applicable	

Data Availability

Data availability	Information included in the manuscript?	In which section is the information available? (Reagents and Tools Table, Materials and Methods, Figures, Data Availability Section)
Have **primary datasets** been deposited according to the journal's guidelines (see 'Data Deposition' section) and the respective accession numbers provided in the Data Availability Section?	Yes	Data availability section
Were **human clinical and genomic datasets** deposited in a public access-controlled repository in accordance to ethical obligations to the patients and to the applicable consent agreement?	Not Applicable	
Are **computational models** that are central and integral to a study available without restrictions in a machine-readable form? Were the relevant accession numbers or links provided?	Not Applicable	
If publicly available data were reused, provide the respective **data citations in the reference list**.	Not Applicable	
